# Systematic Investigation of the Effects of Seven Plant Extracts on the Physiological Parameters, Yield, and Nutritional Quality of Radish (*Raphanus sativus* var. *sativus*)

**DOI:** 10.3389/fpls.2021.651152

**Published:** 2021-06-17

**Authors:** Katarzyna Godlewska, Paweł Pacyga, Izabela Michalak, Anita Biesiada, Antoni Szumny, Natalia Pachura, Urszula Piszcz

**Affiliations:** ^1^Department of Horticulture, Faculty of Life Sciences and Technology, Wrocław University of Environmental and Life Sciences, Wrocław, Poland; ^2^Department of Energy Technologies, Turbines, and Modeling of Heat-Flow Processes, Faculty of Mechanical and Power Engineering, Wrocław University of Science and Technology, Wrocław, Poland; ^3^Department of Advanced Material Technologies, Faculty of Chemistry, Wrocław University of Science and Technology, Wrocław, Poland; ^4^Department of Chemistry, Faculty of Biotechnology and Food Science, Wrocław University of Environmental and Life Sciences, Wrocław, Poland; ^5^Department of Plant Nutrition, The Faculty of Life Sciences and Technology, Wrocław University of Environmental and Life Sciences, Wrocław, Poland

**Keywords:** higher plants, extraction, bioactive compounds, radish, yield, nutritional quality, sustainable food production

## Abstract

The modern agricultural sector faces the challenge of addressing the needs of the fast-growing global population. This process should be both high-yielding and sustainable, without creating risks for the environment and human health. Therefore, natural products are gaining attention in the production of safe and nutritious food. In a systematic effort to develop affordable and effective biostimulants, we examined the impact of botanical extracts on the growth and physiological parameters of radish plants under field conditions. Ultrasound-assisted extraction, mechanical homogenization, and water were used for the production of potential plant-based biostimulants. Foliar applications of the bio-products, developed and used in our study, have led to an increase in the examined parameters (total yield, dry weight, photosynthetic pigments, vitamin C, nitrates, and micro- and macroelements). A decrease in the total phenolic compounds content was also noted, as well as a varied impact on the steam volatile compounds, fatty acids, sterol, and glucosinolates composition. The most beneficial effects on radish, in terms of physiological and biochemical properties, were found in groups treated with extracts based on the common dandelion, valerian, and giant goldenrod. This innovative approach presented in our study could provide a valuable tool for sustainable horticultural production.

## Introduction

Globally, agriculture and horticulture constitute a multitrillion dollar industry. This sector provides a wide variety of crops for food, feed, and ornamental purposes (Zulfiqar et al., 2019). Due to the exponentially growing population, limited available farmland, genetic potential of crops, depletion of natural resources, and climate change, agribusiness is facing the challenge of devising more effective and sustainable solutions to facilitate the reduction of malnutrition, poverty, starvation, and energy and water usage while concurrently increasing the yield and the quality of crops (Campos et al., 2019; Pereira et al., 2019; Szparaga et al., 2019; Zulfiqar et al., 2019; Kamble et al., 2020). The Green Revolution implemented in the 1960s was characterized by large increases in crop yields due to the extensive use of pesticides and fertilizers (Pereira et al., 2019; Rose et al., 2019). The long-standing excess usage of these products has led to serious threats to human health and the environment worldwide (e.g., ground water and air pollution, water eutrophication, and soil quality degradation) (Ertani et al., 2014; Vejan et al., 2016; Campos et al., 2019; Costa et al., 2019; Ekin, 2019; Pereira et al., 2019; Shang et al., 2019; Shukla et al., 2019; Zulfiqar et al., 2019). On the other hand, these products have been necessary to satisfy the growing human demand for food. Furthermore, the overuse of fertilizers has increased the cost of production and reduced the profit margins for farmers (Zulfiqar et al., 2019). Climate change and unfavorable growing conditions can increase plant susceptibility to pathogens (Shukla et al., 2019). Consequently, the establishment of sustainable principles, strategies, technological advancements, and innovations (Pereira et al., 2019; Shang et al., 2019) is crucial to enhance the effectiveness of fertilizers, to meet the requirements of environmentally friendly crop management practices, and to cope with high productivity demands (Bulgari et al., 2015; Paradiković et al., 2018; Ekin, 2019; Shang et al., 2019; Shukla et al., 2019). Crop resistance to diseases, soil salinity, drought, heavy metals, as well as their increased nutritional value are getting highly desirable (Vejan et al., 2016; Bulgari et al., 2019).

Bio-based products (including crop residues, plants, and seaweed), applied at low doses, may be a promising solution to diminish fertilizer rates and simultaneously exert beneficial effects on plant growth (Ertani et al., 2014). Biostimulants have become more popular in sustainable agriculture in recent years because of their beneficial properties. They stimulate various physiological processes that promote nutrient acquisition and utilization by plants; enhance the root development (the length and number of root hairs), shoot development, yield, and nutritional quality of plants; counteract the effects of biotic and abiotic stresses; improve the activity of soil microbiota; and reduce the use of fertilizers and the content of undesirable compounds (e.g., nitrates and heavy metals) in cultivated plants (Ertani et al., 2014; Bulgari et al., 2015; Paradiković et al., 2018; Rouphael et al., 2018; Szparaga et al., 2018; Carillo et al., 2019a,b; Ekin, 2019; Shukla et al., 2019; Cozzolino et al., 2020). The effects of biostimulants may differ, depending on the plant species (e.g., different leaf permeability levels), cultivar, physiological stages, type of product, dose, concentration, time, and application method (e.g., foliar, soil drench, or seed treatment), as well as environmental conditions (Ertani et al., 2014; Bulgari et al., 2015; Paradiković et al., 2018; Szparaga et al., 2018). In the European Union, the countries of France, Italy, and Spain are the primary producers of biostimulants. As reported by Grand View Research, Inc., the market size of these products is projected to be worth approximately USD 4.14 billion by 2025 (Bulgari et al., 2019). According to the new European Union Regulation, 2019/1009, plant biostimulants are defined on the basis of their agricultural impacts on crops. They may contain protein hydrolysates, humic substances, algal and botanical extracts, inorganic compounds (e.g., Si), growth-promoting bacteria, mycorrhizal fungi (D'Addabbo et al., 2019; Cozzolino et al., 2020), amino acids, chitin, chitosan, vitamins, and poly- and oligosaccharides (Bulgari et al., 2015). The beneficial effects of biostimulants are not associated with their macro– and micronutrient composition but rather with their content of activating compounds, like endogenous hormones, small peptides, free amino acids, phenolics, and triacontanol (Ertani et al., 2014; Yakhin et al., 2017), which may affect plant metabolisms by triggering glycolysis enzyme activities, the Krebs cycle, and nitrate assimilation (Ertani et al., 2009; Colla et al., 2015, 2017; Yakhin et al., 2017; Palumbo et al., 2018; Sandhu et al., 2018; Alfosea-Simón et al., 2020; Cozzolino et al., 2020; Francesca et al., 2020). On the other hand, if their biological activity depends on the presence of natural plant hormones, they ought to be classified as plant growth regulators (Bulgari et al., 2015). The hormonal activity of plants can alter the electrochemical gradient of protons formed across the cell membrane through proton pump modulation (Paradiković et al., 2018). By virtue of the complexity of biostimulants' composition, it is difficult to assign their beneficial effects on plants to a particular compound, especially when that compound may interact with other molecules in a synergistic way (Bulgari et al., 2015, 2019). Hence, their mechanism of action is still not well-understood, and these types of products should be categorized based on the physiological responses of plants (Bulgari et al., 2015).

In recent years, numerous reports have highlighted the beneficial effects of biostimulants applications in the worldwide cultivation of various crops, especially for achieving higher yields. New research should focus not only on short-term studies of young seedlings but also on real field conditions with mature plants (Ertani et al., 2014). Nevertheless, the impact of the application of bio-products on the nutritional value and health-promoting potential of plants is not well-documented but has been observed in several crops (Kocira, 2019; Cocetta and Ferrante, 2020). Many of these compounds exhibit a favorable impact on the health-related characteristics of vegetables and fruits (Cocetta and Ferrante, 2020)—for example, the presence of antioxidants imparts nutraceutical properties to plant products (Kocira, 2019). Presently, fruits, vegetables, and edible flowers rich in phytonutrients (plant-derived substances; neither vitamins nor minerals) are gaining more attention both among scientists and consumers. These are usually plant secondary metabolites (synthesized from primary metabolites) and are involved in various mechanisms, e.g., in plant interaction with the environment, defense responses to stresses (by serving as phytoalexins, antioxidants, and signal molecules), as well as to deter animals or, conversely, to attract them to spread seeds or pollinate flowers (due to their anthocyanin content). These compounds often function as antioxidants; they increase the antioxidant potential of fruits, vegetables, and flowers, and they obstruct oxidative reactions under stress conditions (Bulgari et al., 2015). A diet low in fruits and vegetables is associated with an increased risk of various debilitating nutritional diseases. As reported by the Global Burden of Disease Study, a low intake of fruits can be attributed to 3.4 million deaths, while low vegetable consumption was estimated to cause 1.8 million demises globally (Bulgari et al., 2019). Presently, healthy lifestyles and high-quality foods are piquing a growing interest among consumers. Therefore, there is a need to develop new, cheaper products for use in organic agriculture to enable greater access to more affordable and eco-friendly food (Kowalski and Kaniszewski, 2017).

Radish (*Raphanus sativus* var. *sativus*) was chosen as a model plant in the present study. There are numerous radish cultivars differing in shape (round, oval, icicle, half long, long, conical, cylindrical, spindle), color (white, pink, red, purple, black), flavor, and growing conditions (Paul et al., 2016; Dhaliwal, 2017). Radish is widely cultivated due to its taste and low content of calories but high nutrients (e.g., K, Fe, Ca, Na, Zn, Mn, P, vitamins C and B, folic acid, fiber) (Paul et al., 2016; Banihani, 2017; Dhaliwal, 2017; Kowalski and Kaniszewski, 2017). This root vegetable is valued for the presence of phytochemicals, especially glucosinolates, which are hydrolyzed into bioactive compounds (e.g., isothiocyanates, nitriles, thiocyanates, epithionitriles, and oxazolidinethiones) that are of use to plants (e.g., for defense) and human health. The consumption of radish has been shown to lower the risk of different types of cancers (e.g., breast, colon, lung, stomach, prostate, pancreas, and rectal cancer), as well as supports the prevention of constipation, stone formation, and jaundice (Paul et al., 2016; Banihani, 2017; Dhaliwal, 2017; Manivannan et al., 2019). Radish also exhibits antimicrobial, anticancer, antioxidant, and anxiety-reducing properties (Manivannan et al., 2019). In Poland, the cultivation area of radish is estimated at 700 ha and field cultivation accounts for one-third of the whole area (Chohura and Kołota, 2011). Due to the low light in the autumn–spring period, the overaccumulation of nitrates in plant tissues may sometimes be a problem (Kowalski and Kaniszewski, 2017).

Hence, our current study aimed to determine the possibility of transforming higher plants that are widely found in Europe (herbs of St. John's wort, leaves of giant goldenrod, flowers and leaves of common dandelion, flowers of red clover, leaves of nettle, and roots of valerian) into low-cost products that are rich in bioactive compounds for use in modern horticulture to achieve higher yield, quality, and profitability. Ultrasound-assisted extraction (UAE) and mechanical homogenization (MH) were used for the production of potential water-based biostimulants. The selected raw materials were not previously used for these purposes.

## Materials and Methods

All analyses were carried out according to the methodology provided by Godlewska et al. (2020b). Brief descriptions of our methodology are presented below.

### Raw Materials for the Botanical Extract Production

This study assessed the potential of using higher plants for the production of plant-based extracts. The raw materials collection date depended on the plant part used for the extraction and the plant developmental stage. They were collected/purchased once in 2017 in the amount needed to carry out all planned research. The extraction of biologically active compounds from St. John's wort (*Hypericum perforatum* L.; herb) (marked as: Hp H), giant goldenrod (*Solidago gigantean* Ait.; leaf) (Sg L), common dandelion (*Taraxacum officinale* (L.) Weber ex F.H. Wigg; flower, leaf) (To F, To L), red clover (*Trifolium pretense* L.; flower) (Tp F), nettle (*Urtica dioica* L.; leaf) (Ur L), and valerian (*Valeriana officinalis* L.; root) (Vo R) was performed using ultrasound-assisted extraction (UAE) and mechanical shearing combined with sonic energy (MH). For the first method, the dried and ground biomass was mixed with deionized water (1:20 *w*/*v*), soaked (30 min), subsequently sonicated (30 min), and then centrifuged (10 min, 4,500 rpm). For the second method (MH), the mixture was homogenized (1 min, 28,000 rpm) and centrifuged (10 min, 4,500 rpm). The final bioactive formulations were composed of an active ingredient (extract, 0.5% *w*/*v*), an antioxidant agent (L-ascorbic acid, 0.15% *w*/*v*), an adjuvant (Protector, 0.02% *w*/*v*), a preservative (potassium sorbate, 0.1% *w*/*v*), and water (up to 100%).

### The Field Trials

The radish (*Raphanus sativus* var. *sativus*) was grown in the field under a temperate climate in Poland (Supplementary Figure 1). Hydrocomplex Yara Mila (250 kg·ha^−1^) and ammonium saltpetre (330 kg·ha^−1^) were used for the fertilization of the fine clay soil (pH 7.05, EC 144.3 μS·cm^−1^, 1.8% humus, 35.1 mg P, 89.5 mg K, and 44 mg Mg in 1 dm^3^). The experiments were performed in randomized complete blocks. Seeds (cultivar “Carmen,” PlantiCo) were sown on August 20, 2018, with spacing of 20 cm × 4 cm (plot size: 1 m^2^; 125 plants per plot; 3 plots per treatment). The spraying, at a dose of 300 L·ha^−1^, was performed three times in the morning on sunny and windless days (September 5, September 12, and September 19, 2018). Plants were harvested on September 24, 2018. During the growing season, regular mechanical weeding and irrigation were conducted. Moreover, the insecticide (Karate Zeon 050 CS) was applied in accordance with the manufacturer's recommendations. Plant samples were collected twice: 7 days after the second spraying (the first term of leaves of rosette collection) and after harvesting (the second term of leaves of rosette and root collection) to perform chemical analyses. As the control groups, we used plants sprayed with water (C), a formulation with water without an active ingredient (CF), and a commercial biostimulant (CB).

### Chemicals

Acetone, calcium carbonate, ethanol, potassium persulphate, sodium acetate, and sodium carbonate were purchased from IDALIA (Radom, Poland); azino-bis-3-ethylbenzthiazoline-6-sulphonic acid (ABTS), diphenyl-2-picrylhydrazyl (DPPH), ferric-reducing antioxidant power (FRAP), Folin–Ciocalteu's phenol reagent, gallic acid, tripyridyl-S-triazine (TPTZ), and Trolox were purchased from Archem (Łany, Poland); acetic acid, activated carbon, ammonium metavanadate, ammonium molybdate tetrahydrate, ascorbic acid, barium chloride dihydrate, cyclohexane, magnesium nitrate, 65% nitric acid, oxalic acid, sodium bicarbonate, and sodium sulfate were purchased from CHEMPUR (Piekary Śląskie, Poland); 2,6-dichlorophenolindophenol sodium salt hydrate was purchased from ACROS ORGANICS (ARGENTA; Poznań, Poland); chloroform, hydrochloric acid (38%), and methanol were purchased from STANLAB (Lublin, Poland); standard solutions and Tween TM 80 were purchased from Merck (Darmnstadt, Germany); 2-undecanone and BF_3_/MeOH were purchased from Sigma-Aldrich (Saint Louis, MO, USA); hexane and sodium bicarbonate were purchased from UQF (Wrocław, Poland); n-hexane (99%) was purchased from POCH Basic (Gliwice, Poland); helium was purchased from Air Products (Warsaw, Poland); and potassium hydroxide was purchased from Avantor (Gliwice, Poland).

### The Photosynthetic Pigments, Greenness Index of the Leaves, and Leaf Color

The contents of chlorophyll *a* + *b* and carotenoids were determined in fresh leaves. Samples (0.4 g) were disintegrated, using a mortar and a pestle with the addition of a few drops of acetone (80%), a pinch of sand, and calcium carbonate. The obtained mixture was filtered, transferred to a volumetric flask (50 ml) and filled with acetone. The absorbances were measured in four replicates at 663, 645, and 470 nm with the use of a spectrophotometer (HACH DR1900, Berlin, Germany). The greenness indexes of the leaves were evaluated, using an SPAD 502 Plus Chlorophyll Meter (Konica Minolta, Osaka, Japan), and the colors of the leaves were measured, using MiniScan (Hunter Lab EZ, Reston, Virginia, USA) (in 10 replicates).

### Vitamin C

For determination of the vitamin C content, the fresh leaves (~10 g) and roots (~15 g) were homogenized in oxalic acid (200 ml, 2%) and filtrated. Solutions (10 ml) were titrated (in four replicates) with Tillmans' reagent as long as a light pinkish color appeared and was maintained for at least 30 s.

### Total Phenolic Compounds

The total phenolic compound (TPC) content was evaluated in fresh, comminuted shoots and roots (~2 g). The biomasses were placed in tubes, mixed with aqueous methanol (20 ml, 80%), sonicated (15 min), and centrifuged (10 min, 4,500 rpm). To the obtained supernatants (0.1 ml), the Folin–Ciocalteu's phenol reagent (0.2 ml) and distilled water (2 ml) were added, and the mixtures were kept in the dark (3 min). Next, sodium carbonate (1 ml, 20%) was added to the reaction mixtures and left in the dark for 1 h. The absorbance (765 nm) was then assessed (in four replicates).

### The Antioxidant Activity (DPPH, ABTS, and FRAP Assays)

The antioxidant activities were assessed in ten-fold diluted supernatants prepared for TPC analyses. The absorbance measurements were made in four replicates.

For the DPPH assay, supernatants (0.5 ml), ethanol (1.5 ml), and DPPH solution (0.5 ml) were mixed and incubated at room temperature without access to light (10 min). The radical stock solution of DPPH was freshly prepared by dissolving in ethanol.

For the ABTS assay, a diluted ABTS solution (3 ml) was added to the supernatants (30 μl) and kept in the dark (6 min). The ABTS^•+^solution was prepared by the reaction of aqueous ABTS solution (5 ml, 7 mM) with potassium persulfate solution (88 μl, 140 mM). The mixture was incubated in the dark at 29°C for more than 14 h.

For the FRAP assay, supernatants (1 ml) and FRAP reagent (3 ml) were left to react (10 min). The FRAP reagent was prepared by mixing the acetate buffer (300 mM), TPTZ (10 ml in 40 mM HCl) and FeCl_3_·6H_2_O (20 mM) in a ratio of 10:1:1.

For the DPPH assay, the absorbance was measured at 517 nm; for the ABTS assay, the absorbance was measured at 734 nm; and for the FRAP assay, the absorbance was measured at 593 nm.

### Nitrates

For the assessment of nitrates, the dried and ground samples (0.4 g) were mixed with acetic acid (100 ml, 2%) and activated carbon (0.5–1 g), shaken (30 min, 150 rpm), and filtrated (the first drops were not collected). An ionometer (Thermo 5 Star Orion, Beverly, MA, USA) with an ion-selective electrode was used for the measurements.

### Macroelements, Microelements, and Heavy Metals

For the macroelement (Ca, K, Mg, P), microelement (Cu, Fe, Mn, Zn), and heavy metal (Ni, Cd, Pb) content evaluations, the dried and ground samples were mineralized (450°C, 8 h) in the oven (CZYLOK, Jastrzębie-Zdrój, Poland); then, the ash was digested (65% HNO_3_) and evaporated on a heating plate (110°C, 6 h), dissolved (1 M HNO_3_), and transferred to a flask. The phosphorus content of P was determined colorimetrically at 400 nm after a reaction with molybdate and ammonium metavanadate (Cecil CE 2011 photometer; Cambridge, UK). Atomic absorption spectrophotometry (ASA) (Varian Spectra AA 220/FS, Mulgrave, Australia) was used for the determination of the K, Ca, Mg, Mn, Fe, Cu, Zn, Cd, Pb, and Ni content. The S content was examined according to the oxidation of sulfur-based on the turbidity of the solution of sulfate content precipitating as barium sulfate (Cecil CE 2011 photometer; Cambridge, UK). The N content was examined by the Kjeldahl method. The samples were mineralized in concentrated H_2_SO_4_ with the addition of Se and H_2_O_2_. The cooled solution was mixed with a strong base solution, and the emitted ammonia was distilled into a saturated boric acids solution with the addition of a mixed indicator (methyl red and bromocresol green). The solution of ammonia in boric acid was determined by titration with a standard hydrochloric acid solution (0.01 M) until an initial color was obtained.

### Steam Volatile Compounds

For the steam volatile compounds analyses (the amount of a single component calculated as a percentage (%) of the whole GC-MS chromatogram area), the frozen leaves (30 g) were placed in flasks and boiled with distilled water (100 ml) in a heating mantle. The cyclohexane (1 ml), containing 1 mg of 2-undecanone, was used as an internal standard. A distillation process (50 min) was performed, using a Deryng apparatus (Szczepanik et al., 2018). The chromatographic analyses (GCMS QP 2020, Shimadzu, Kyoto, Japan) were carried out in three replicates.

### Fatty Acids

For the preparation of the lipid fraction (the amount of a single component calculated as a percentage (%) of the whole GC-MS chromatogram area), the dried roots (350 mg) were macerated with chloroform (5 ml), filtered, and evaporated. The extracted non-polar lipid fraction (25 mg) was saponified (5 min at 65°C) with 0.5 M KOH/MeOH solution (2 ml), and subjected to methylation (10 min at 65°C) by adding 14% (*v*/*v*) BF_3_/MeOH (2 ml). In the next step, distilled water (5 ml) was added, and the methyl esters of fatty acids were extracted with hexane (10 ml). The mixture was then washed with 10% sodium bicarbonate (10 ml) and dried over anhydrous sodium sulfate. The organic phase was evaporated under reduced pressure and dissolved in hexane (200 μl). The gas chromatograph coupled with a mass spectrometer (Shimadzu GCMS QP 2020) was used to analyze the profiles of fatty acid methyl esters (in three replicates).

### Sterols

A dried radish root (300 mg) was macerated with chloroform (5 ml). The sterol profile was evaluated, using the method of derivatization with N, O-Bis(trimethylsilyl)trifluoroacetamide (BSTFA) silylation via GC-MS (Shimadzu QP 2020, Shimadzu, Kyoto, Japan). The solution was filtered and then evaporated on a vacuum evaporator under reduced pressure. Next, pyridine (500 μl) and BSTFA (50 μl) were added to the sample. The mixture was then transferred to a vial and heated for 15 min at 70°C. Separation was achieved using a Zebron ZB-5 capillary column (30 m, 0.25 mm, 0.25 μm; Phenomenex, Torrance, CA, USA). The parameters of the GC-MS analysis were as follows: a scan mode with a mass range from 40 to 1,050 *m*/*z* in the electronic impact (EI) mode at 70 eV with the 10 scan s^−1^ mode. Helium was used as a carrier gas at a flow rate of 1 ml·min^−1^ in a split ratio of 1:20, along with the following program: (a) 100°C for 1 min; (b) a rate of 2°C min^−1^ from 100 to 190°C; and (c) a rate of 5°C min^−1^ from 190 to 300°C. The injector was maintained at 280°C. Identification of the compounds was performed, using two different analytical methods to compare the retention times with those of authentic chemicals (Supelco C7–C40 Saturated Alkanes Standard), and the mass spectra were obtained with the available library data (Willey NIST 17, match index > 90%).

### Glucosinolates

Freeze-dried samples of radish roots (0.5 g) were extracted with 90% methanol (10 ml, 30 min, 70°C, shaken every 5 min). After this process, the samples were filtered and centrifuged (10 min, 15,000 rpm). The supernatants were separated; methanol was evaporated from the mixtures. Then, the samples were dissolved in water (1 ml), and the LC-MS analyses were carried out by means of reversed-phase high-performance liquid chromatography (HPLC Shimadzu Prominence-i LC-2030C, Kyoto, Japan), equipped with a PDA detector coupled to a triple quadrupole mass spectrometer (Shimadzu LCMS-8045). Glucosinolates present in the radish roots were semi-quantified, using the LC-MS-tq apparatus in the MRMs mode (Table 1). The separation of the desired compounds was performed, using the following mobile phase: water with 0.1% TFA (eluent A) and acetonitrile with 0.1% TFA (eluent B). The flow rate was set at 0.25 ml·min^−1^, and the gradient was as follows: starting at 1% solvent B for 3 min, then reaching 20% up to 20 min, 30% up to 23 min, and 0.1% B at 35 min. The Kinetex C18 100A column (100 × 3 mm, 2.6 μm particle size, Phenomenex, Germany) was used. Singrin was used as an external standard (Ciska et al., 2000; Ediage et al., 2011; Maldini et al., 2017).

**Table 1:** A list of multiple reaction monitoring (MRM) transitions and mass spectrometric conditions used for the identification of glucosinolates in radish roots.

	**RT, min**	**MRM transition *m*/*z* (Q1 → Q3)**	**Q1, V**	**CE, V**	**Q3, V**
Glucoerucin	3.84	420.3 → 357.1	20.0	12.0	25.0
		420.3 → 330.0	20.0	17.0	15.0
		420.3 → 258.0	20.0	25.0	26.0
Gluconapin	4.27	372.3 → 354.1	18.0	14.0	24.0
		372.3 → 130.1	18.0	25.0	25.0
		372.3 → 127.9	18.0	22.0	24.0
Progoitrin	10.73	388.3 → 342.3	19.0	12.0	22.0
		388.3 → 128.5	19.0	22.0	25.0
		388.3 → 62.0	18.0	14.0	14.0
Glucoraphenin	11.76	434.3 → 388.2	15.0	11.0	26.0
		434.3 → 371.1	20.0	13.0	27.0
		434.3 → 344.0	21.0	18.0	24.0
4-metoxyglucobrassicin	15.56	476.5 → 386.2	23.0	18.0	26.0
		476.5 → 325.1	16.0	14.0	11.0
		476.5 → 313.9	23.0	25.0	21.0
Glucobrassicin	17.16	446.5 → 400.2	12.0	12.0	18.0
		446.5 → 428.2	21.0	13.0	15.0
		446.5 → 358.0	12.0	20.0	24.0
Glucoraphanin	18.25	435.5 → 399.2	21.0	13.0	27.0
		435.5 → 372.1	15.0	12.0	18.0
		435.5 → 273.2	15.0	24.0	17.0
Gluconasturtiin	18.41	421.6 → 358.1	20.0	12.0	24.0
		421.6 → 277.5	11.0	14.0	29.0
		421.6 → 146.1	19.0	30.0	28.0
Glucobrassicanapin	28.25	385.6 → 349.1	14.0	15.0	23.0
		385.6 → 233.1	18.0	21.0	22.0
		385.6 → 327.2	10.0	19.0	21.0

*RT, retention time; Q1, parent ion; Q3, fragment ion; CE, collision energy*.

### Statistical Analyses

The STATISTICA program ver. 13.3 (TIBCO Software Inc., Tulsa, OK, USA) was used for the statistical analyses of the results. The normality of the distribution was assessed, using the Shapiro–Wilk test and homogeneity of variances by the Brown–Forsythe test. For the normal distribution and homogeneity of variances, differences were evaluated, using the Tukey's Honest Significant Difference (HSD) test. The data were significantly different for *p* < 0.05. For distributions other than normal, the Kruskal–Wallis test was used. An “a” was used to indicate statistically significant differences between the botanical extracts and water (C), “b” indicates a significant difference between the botanical extracts and the formulation (CF), and “c” indicates a significant difference between the botanical extracts and the commercial biostimulant (CB).

## Results

### Total Yield, Fresh, and Dry Weight of Leaves of Rosette and Roots

In our experimental design, the first control group contained plants that were sprayed with water (C), the second one included plants sprayed with the formulation in water (without an active ingredient—extract) (CF), and the third one included plants sprayed with the commercial biostimulant (CB).

The application of all botanical extracts significantly increased the total yield of radish (Table 2). The highest root yield was achieved in the groups treated with To L MH and Sg L MH (81.2 and 80.6% more than in C and 24.9 and 24.5% more than in CB, respectively), while the lowest yield was found with Ur L MH and Ur L UAE (44 and 44.2% more than in C; 0.7 and 0.6% less than in CB). The largest yield of leaves of rosette was observed after the application of Sg L MH and Hp H MH (31.3 and 27.6% more than in C; 15.4 and 12.2% more than in CB), and the smallest was found for To F UAE and Ur L MH (2.7 and 7.2% more than in C; 9.7 and 5.4% less than in CB). Bio-products also promoted the growth of radish roots with diameters >4 cm (e.g., Tp F UAE: 10.2% more than in CB), as well as the aboveground parts (e.g., Hp H MH and Sg L UAE: 32.8 and 34% more than in CB). There were no plants of the aforementioned size in the control group (C). The least stimulating activity in this root range was noted after the use of Hp H UAE for roots (35.7% less than in CB) and Vo R UAE for leaves of rosette (8.2% less than in CB). The heaviest average root weight with a diameter between 2 and 4 cm was found in the groups treated with To L MH (50.7 and 24.4% more than in C and CB) and Sg L MH (42.7 and 17.9% more than in C and CB), and the heaviest leaves were found for Hp H MH (35 and 14.9% more than in C and CB), Sg L MH (34.6 and 14.5% more than in C and CB), and Vo R MH (31.4 and 11.8% more than in C and CB). Decreased fresh weight was observed only in the leaves of rosette after the application of To F UAE (4.3 and 18.5% less than in C and CB). The highest average weight of the root with a diameter smaller than 2 cm was observed after spraying radish with Vo R MH (4.8% less than in C and 67% more than in CB) and Sg L MH (8.8% less than in C and 60% more than in CB), while the lowest was found with Ur L UAE (40.5% less than in C and 4.4% more than in CB). The heaviest weight of the aboveground parts within this root diameter was achieved after radish treatment with To F UAE (2.4% less than in C and 20.4% more than in CB) and Vo R MH (3.9% less than in C and 18.5% more than in CB), and the lowest was found with To L MH (30.4 and 14.1% less than in C and CB) and Ur L UAE (29.4 and 13% less than in C and CB). The application of Ur L UAE increased the dry weight of leaves of rosette (Table 2) in both the first and the second terms of the plant collection (7.5 and 8.3% more than in C; 17.2 and 10.9% more than in CB), while To L MH decreased the examined parameter (12.7 and 13.9% less than in C; 4.8 and 11.9% less than in CB). In the case of roots (Table 2), Ur L UAE increased the dry weight the most (37.9 and 51.8% more than in C and CB), and Sg L MH increased dry weight the least (7.3% less than in C and 2.1% more than in CB).

**Table 2:** Effect of the foliar application of the botanical extracts on the fresh weight of leaves of rosette and root (after harvest), the total yield of radish (*N* = 3^*^, mean ± SD), and dry weight of radish leaves of rosette (after second spraying and after harvest) and root (after harvest) (*N* = 3, mean ± SD).

**Group**	**Root diameter <2 cm**		**Root diameter 2–4 cm**		**Root diameter** **>4 cm**		**Total yield**		**Total yield minus non-marketable yield**	**Dry weight**		
	**Leaves of rosette**	**Root**	**Leaves of rosette**	**Root**	**Leaves of rosette**	**Root**	**Leaves of rosette**	**Root**		**Leaves of rosette after 2nd spraying**	**Leaves of rosette**	**Roots**
	**g**						**t·ha**^****−1****^		**%**	**%**		
C	8.46 ± 0.88b,c	7.93 ± 1.52b,c	9.14 ± 1.12c	17.67 ± 1.70b,c	0b,c	0b,c	11.04 ± 0.99c	15.76 ± 2.59b,c	97.64	8.55 ± 0.34	8.54 ± 0.41	3.72 ± 0.37
CF	6.52 ± 0.54a	5.59 ± 0.47a,c	10.57 ± 1.14	22.91 ± 3.10a	29.5 ± 03.27a,c	53.00 ± 5.72a,c	12.24 ± 1.34	24.35 ± 3.22a	98.51	8.37 ± 0.31	8.04 ± 0.25	3.87 ± 0.30
CB	6.86 ± 0.54a	4.52 ± 0.52a,b	10.74 ± 0.49a	21.40 ± 1.52a	17.39 ± 2.56a,b	61.54 ± 8.83a,b	12.56 ± 1.49a	22.86 ± 3.22a	94.93	7.84 ± 0.19	8.34 ± 0.39	3.38 ± 0.40
Hp H UAE	6.30 ± 0.85a	7.09 ± 1.60b,c	11.42 ± 0.51a	24.87 ± 1.00a,c	19.15 ± 2.00a,b	39.60 ± 6.52a,b,c	12.41 ± 1.27a	24.24 ± 3.05a	95.10	8.25 ± 0.29	8.72 ± 0.40	4.06 ± 0.07
Hp H MH	7.18 ± 1.53a	5.68 ± 0.36a,c	12.34 ± 2.41a,b,c	24.16 ± 3.35a,c	23.10 ± 2.20a,b,c	55.40 ± 2.78a,c	14.09 ± 1.93a,b,c	25.28 ± 2.80a	98.26	8.59 ± 0.18	8.06 ± 0.46	4.10 ± 0.38
Sg L UAE	6.76 ± 0.98a	5.53 ± 0.68a,c	10.96 ± 1.63a	22.19 ± 1.72a	23.30 ± 3.92a,b,c	60.45 ± 8.61a,b	12.74 ± 1.87a	23.96 ± 3.03a	98.06	7.72 ± 0.10	8.86 ± 0.43	3.64 ± 0.14
Sg L MH	7.85 ± 1.69b,c	7.23 ± 1.70b,c	12.30 ± 2.97a,b,c	25.22 ± 2.68a,c	18.94 ± 3.74a,b	59.35 ± 2.20a,b	14.49 ± 2.75a,b,c	28.46 ± 4.15a,b,c	97.70	7.88 ± 0.18	8.30 ± 0.33	3.45 ± 0.32
To F UAE	8.26 ± 1.14b,c	6.28 ± 1.29a,c	8.75 ± 1.43b,c	21.58 ± 3.37a	16.26 ± 0.71a,b	47.43 ± 5.55a,b,c	11.34 ± 1.63	23.58 ± 3.93a	98.38	8.27 ± 0.22	7.68 ± 0.49	4.25 ± 0.35
To F MH	7.83 ± 0.19b,c	6.72 ± 1.48a,b,c	11.76 ± 0.54a	23.39 ± 2.81a	20.65 ± 4.67a,b,c	47.63 ± 8.95a,b,c	13.84 ± 1.70a,b	25.31 ± 4.90a	94.89	8.36 ± 0.28	8.26 ± 0.26	3.83 ± 0.05
To L UAE	7.20 ± 0.79a	5.38 ± 0.30a	11.72 ± 0.68a	23.49 ± 2.98a	20.75 ± 2.25a,b,c	58.25 ± 5.19a,b	13.65 ± 1.25a,b	25.77 ± 2.93a,c	95.58	8.07 ± 0.22	8.85 ± 0.28	3.75 ± 0.23
To L MH	5.89 ± 0.69a,c	4.97 ± 0.49a	11.35 ± 2.37a	26.63 ± 5.39a,b,c	19.10 ± 2.78a,b	53.57 ± 5.25a,c	13.09 ± 1.83a	28.55 ± 4.11a,b,c	94.59	7.46 ± 0.38a	7.35 ± 0.32	3.62 ± 0.13
Tp F UAE	7.01 ± 1.33a	5.35 ± 0.87a	11.84 ± 1.46a	23.48 ± 2.73a	22.05 ± 2.90a,b,c	67.80 ± 4.74a,b,c	13.22 ± 1.80a	23.65 ± 3.60a	97.80	7.88 ± 0.40	8.56 ± 0.43	4.13 ± 0.33
Tp F MH	6.66 ± 1.23a	6.10 ± 0.16a,c	11.42 ± 1.31a	23.31 ± 1.96a	19.29 ± 4.91a,b	51.10 ± 0.99a,c	13.30 ± 2.21a	25.45 ± 1.58a,c	98.17	8.31 ± 0.25	8.38 ± 0.23	3.90 ± 0.19
Ur L UAE	5.97 ± 1.03a	4.72 ± 1.09a	10.55 ± 1.60	20.36 ± 1.42a,b	19.72 ± 3.57a,b	54.17 ± 2.43a,c	12.09 ± 1.91	22.73 ± 2.98a	94.47	9.19 ± 0.16 c	9.25 ± 0.36	5.13 ± 0.33a,b,c
Ur L MH	6.29 ± 0.78a	5.19 ± 0.62a	10.04 ± 0.07	20.60 ± 1.77a	20.26 ± 3.79a,b,c	54.06 ± 7.59a,c	11.84 ± 1.22	22.69 ± 2.59a	96.52	8.58 ± 0.27	8.68 ± 0.44	3.57 ± 0.40
Vo R UAE	6.36 ± 0.94a	5.31 ± 0.53a	11.35 ± 1.37a	24.61 ± 2.95a,c	15.97 ± 2.22a,b	57.63 ± 5.41a	12.69 ± 1.39a	25.48 ± 3.31a,c	97.44	8.43 ± 0.13	7.99 ± 0.39	4.02 ± 0.22
Vo R MH	8.13 ± 1.46b,c	7.55 ± 1.43b,c	12.01 ± 2.29a,b	23.83 ± 2.51a	17.23 ± 3.37a,b	48.57 ± 3.13a,c	13.97 ± 2.38a,b,c	26.35 ± 3.42a,c	95.52	8.23 ± 0.19	8.04 ± 0.40	4.42 ± 0.30

*(a) Statistically significant differences (p < 0.05) between the control group (C) and the botanical extracts. (b) Statistically significant differences (p < 0.05) between the formulation (CF) and the botanical extracts. (c) Statistically significant differences (p < 0.05) between commercial biostimulant (CB) and the botanical extracts. UAE, ultrasound-assisted extraction; MH, mechanical homogenization; Hp H, Hypericum perforatum L. (St. John's wort, herb); Sg L, Solidago gigantea Ait. (giant goldenrod, leaf); To F, To L, Taraxacum officinale (L.) Weber ex F.H. Wigg (common dandelion, flower, leaf); Tp F, Trifolium pratense L. (red clover, flower); Ur L, Urtica dioica L. (nettle, leaf); Vo R, Valeriana officinalis L. (valerian, root)*.

* *Three replications (plots)—each consisted of 125 plants*.

### Photosynthetic Pigments, Greenness Index of the Leaves, and Leaf Color

An increase in the content of photosynthetic pigments in the cultivated plants treated with botanical extracts was observed in the first term of the sample collection but showed varying impacts in the second term. In the first term, the bio-product Vo R UAE particularly increased the chlorophyll content (Table 3) (31.5 and 26.3% more than in C and CB), whereas To L MH decreased its content (16.4 and 19.7% less than in C and CB). In the second term (Table 3), the application of Vo R UAE, Sg L MH, and Ur L MH resulted in a slightly higher amount of a green pigment in leaves (4.5, 4.5, and 3% more than in C; 16.7, 16.7, and 15% more than in CB). The use of To L UAE, To F MH, and Sg L UAE caused a significant reduction in its content (19.4, 19.4, and 17.9% less than in C; 10, 10, and 8.3% less than in CB). In the first term, the highest content of carotenoids (Table 3) was observed after the application of Vo R UAE (31.3 and 19.9% more than in C and CB), like in the case of chlorophyll content. In the second term, this extract showed the most stimulating activity, but the difference was nonsignificant in comparison to the control group (1.2 and 14.4% more than in C and CB). The lowest carotenoid content was observed for To L MH (in the first term: 28.7 and 34.9% less than in C and CB) and for To F MH (in the second term: 19.7 and 9.3% less than in C and CB).

**Table 3:** Effect of the foliar application of the botanical extracts on the chlorophyll *a* + *b* content (*N* = 4, mean ± SD), the carotenoids content (*N* = 4, mean ± SD), the *L, a, b* values (*N* = 10, mean ± SD), and the SPAD values (*N* = 10, mean ± SD) of radish leaves of rosette (after second spraying and after harvest).

**Group**	**Chlorophyll** ***a*** **+** ***b***		**Carotenoids**		**Color**						**Greenness indexes**	
	**After 2nd spraying**	**After harvest**	**After 2nd spraying**	**After harvest**	**After 2nd spraying**			**After harvest**			**After 2nd spraying**	**After harvest**
	**mg·g**^****–1****^ **FW**		**μg·g**^****–1****^ **FW**		***L***	***a***	***b***	***L***	***a***	***b***	**SPAD values**	
C	0.73 ± 0.06	1.34 ± 0.03	16.94 ± 1.15	28.47 ± 1.19	42.05 ± 2.71	−9.36 ± 0.65	21.04 ± 2.01	42.23 ± 0.49	−8.66 ± 0.25	18.25 ± 0.76	36.53 ± 1.28	34.72 ± 1.23
CF	0.82 ± 0.03	1.23 ± 0.04	20.01 ± 1.31	25.33 ± 0.69	43.77 ± 0.79	−8.40 ± 0.34	19.12 ± 0.50	41.18 ± 1.10	−8.78 ± 0.23	18.25 ± 0.87	35.95 ± 1.80	35.72 ± 1.53
CB	0.76 ± 0.02	1.20 ± 0.05	18.55 ± 0.92	25.20 ± 0.71	43.53 ± 0.77	−9.18 ± 0.44	21.72 ± 0.91	43.17 ± 0.19	−8.01 ± 0.43	15.87 ± 1.45	35.60 ± 1.08	36.63 ± 0.56
Hp H UAE	0.83 ± 0.06	1.22 ± 0.04	19.04 ± 0.88	25.09 ± 0.59	41.74 ± 0.10	−7.99 ± 0.18a,c	17.82 ± 0.43a,c	41.58 ± 0.61	−8.27 ± 0.17	16.85 ± 0.23	40.82 ± 1.42a,b,c	41.25 ± 2.22a,b,c
Hp H MH	0.75 ± 0.04	1.21 ± 0.05	18.89 ± 0.93	26.74 ± 1.10	41.38 ± 0.73	−8.15 ± 0.04a,c	15.26 ± 0.13a,b,c	40.88 ± 0.26 c	−8.64 ± 0.01	17.38 ± 0.18	45.48 ± 2.02a,b,c	39.65 ± 1.44a,b
Sg L UAE	0.83 ± 0.06	1.10 ± 0.03a	18.05 ± 0.67	24.02 ± 0.98a	40.51 ± 0.38	−8.35 ± 0.03a	17.06 ± 0.27a,c	40.16 ± 0.56 c	−8.52 ± 0.05	18.25 ± 0.35	40.73 ± 2.11a,b,c	40.45 ± 0.63a,b
Sg L MH	0.84 ± 0.08	1.40 ± 0.07	16.58 ± 0.88b	27.95 ± 0.63	41.77 ± 0.34	−9.33 ± 0.05	19.45 ± 0.28	43.01 ± 0.22	−8.91 ± 0.15	17.38 ± 0.43	40.13 ± 1.30a,b,c	39.43 ± 0.79a
To F UAE	0.74 ± 0.04	1.24 ± 0.05	19.67 ± 0.56	26.75 ± 0.85	39.95 ± 1.68	−7.80 ± 0.22a,c	18.80 ± 0.47 c	42.24 ± 0.25	−8.65 ± 0.11	18.85 ± 0.59	38.72 ± 1.12	39.58 ± 1.45a,b
To F MH	0.81 ± 0.04	1.08 ± 0.02a	20.72 ± 0.85a	22.85 ± 1.13a	42.65 ± 0.10	−8.47 ± 0.24	17.34 ± 0.38a,c	40.48 ± 0.51 c	−8.27 ± 0.49	15.43 ± 0.68	41.12 ± 1.21a,b,c	43.72 ± 2.33a,b,c
To L UAE	0.90 ± 0.03	1.08 ± 0.06	19.71 ± 0.75	23.39 ± 0.64a	42.89 ± 0.95	−8.89 ± 0.17	18.62 ± 0.19 c	43.91 ± 0.92b	−8.77 ± 0.54	19.42 ± 1.18 c	39.33 ± 1.88 c	38.85 ± 1.66a
To L MH	0.61 ± 0.05b	1.25 ± 0.07	12.07 ± 0.34a,b,c	24.96 ± 0.90a	40.50 ± 0.84	−9.39 ± 0.14b	19.42 ± 0.34	42.58 ± 0.30	−8.03 ± 0.13	17.54 ± 0.09	40.28 ± 1.15a,b,c	40.18 ± 0.95a,b
Tp F UAE	0.78 ± 0.04	1.36 ± 0.08	17.36 ± 0.75	28.40 ± 1.18	41.24 ± 0.41	−9.14 ± 0.28	19.95 ± 0.56	43.30 ± 0.57	−7.87 ± 0.53	17.03 ± 0.77	38.67 ± 1.03	44.12 ± 2.01a,b,c
Tp F MH	0.82 ± 0.06	1.28 ± 0.05	19.62 ± 1.15	26.42 ± 0.68	40.57 ± 0.53	−9.51 ± 0.10b	19.67 ± 0.52	42.29 ± 0.74	−7.05 ± 0.21a,b	15.94 ± 0.20	38.35 ± 1.69	41.82 ± 2.24a,b,c
Ur L UAE	0.80 ± 0.03	1.16 ± 0.07	19.35 ± 0.72	23.36 ± 0.88a	39.76 ± 0.13b	−7.50 ± 0.07a,c	15.72 ± 0.29a,b,c	40.47 ± 1.13 c	−8.05 ± 0.36	17.31 ± 1.12	41.13 ± 2.22a,b,c	43.60 ± 2.57a,b,c
Ur L MH	0.71 ± 0.03	1.38 ± 0.08	16.98 ± 1.00	28.74 ± 1.15c	40.95 ± 0.46	−8.30 ± 0.20a	19.63 ± 1.26	40.10 ± 0.09 c	−8.77 ± 0.12	17.61 ± 0.09	38.70 ± 0.93	39.35 ± 1.94a
Vo R UAE	0.96 ± 0.04a,c	1.40 ± 0.09	22.25 ± 0.96a,c	28.82 ± 1.11c	42.67 ± 2.06	−8.83 ± 0.28	18.84 ± 1.10 c	39.52 ± 0.17a,c	−6.56 ± 0.16a,b,c	13.94 ± 0.25a,b	39.23 ± 0.62 c	44.47 ± 2.09a,b,c
Vo R MH	0.67 ± 0.04	1.32 ± 0.06	14.10 ± 0.85b,c	26.86 ± 1.16	42.10 ± 0.29	−9.55 ± 0.05b	20.05 ± 0.17	41.26 ± 0.25	−7.86 ± 0.75	16.80 ± 1.70	38.88 ± 1.53	41.08 ± 1.44b,c

(a) Statistically significant differences (p < 0.05) between the control group (C) and the botanical extracts. (b) Statistically significant differences (p < 0.05) between the formulation (CF) and the botanical extracts. (c) Statistically significant differences (p < 0.05) between commercial biostimulant (CB) and the botanical extracts. UAE, ultrasound-assisted extraction; MH, mechanical homogenization; Hp H, Hypericum perforatum L. (St. John's wort, herb); Sg L, Solidago gigantea Ait. (giant goldenrod, leaf); To F, To L, Taraxacum officinale (L.) Weber ex F.H. Wigg (common dandelion, flower, leaf); Tp F, Trifolium pratense L. (red clover, flower); Ur L, Urtica dioica L. (nettle, leaf); Vo R, Valeriana officinalis L. (valerian, root). ^*^ Three replications (plots)—each consisted of 125 plants.

Most bio-products did not significantly increase the leaf color, expressed as the *L, a, b* values presented in Table 3. The level of light or dark is described by the *L* value, redness or greenness is indicated by the *a* value, and yellowness or blueness is represented by the *b* value. These values are necessary to depict the leaf color.

The SPAD measurements demonstrated that all obtained bio-products increased the greenness index of the leaves (Table 3). In the first term, the greatest enhancement of the SPAD index was noted in the group treated with Hp H MH (24.5 and 27.8% more than in C and CB), while the smallest was found for Tp F MH (5 and 7.7% more than in C and CB). In the second term, the highest stimulating activity was observed for Vo R UAE (28.1 and 21.4% more than in C and CB), which corresponds to the results obtained for chlorophyll, with the lowest observed for To L UAE (11.9 and 6.1% more than in C and CB).

### Vitamin C

In the majority of cases, the novel extracts raised the level of vitamin C in the leaves of rosette and in the roots (Table 4). For instance, in both terms of the samples collection, Vo R MH increased its content (in the first term: 29.9 and 10.5% more than in C and CB; in the second term: 116 and 44.4% more than in C and CB), while To L MH decreased its content (in the first term: 6.9 and 20.7% less than in C and CB; in the second term: 25.8% more than in C and 15.7% less than in CB). However, for the roots, Tp F MH increased the content of vitamin C (46.3 and 33.8% more than in C and CB), while Vo R MH reduced it (10.5 and 18.2% less than in C and CB).

**Table 4:** The effect of the foliar application of the botanical extracts on the vitamin C content (*N* = 4, mean ± SD) and the total phenolic compounds content (*N* = 4, mean ± SD) of radish leaves (after second spraying and after harvest) and roots (after harvest).

**Group**	**Vitamin C**			**Total phenolic compounds**		
	**Leaves of rosette after 2nd spraying**	**Leaves of rosette after harvest**	**Roots after harvest**	**Leaves of rosette after 2nd spraying**	**Leaves of rosette after harvest**	**Roots after harvest**
	**mg·100 g**^****−1****^ **FW**			**mg GAE·100 g**^****−1****^ **FW**		
C	105.33 ± 3.76b,c	52.23 ± 1.31b,c	26.33 ± 1.25b	105.24 ± 4.28c	131.89 ± 3.86	36.73 ± 1.11c
CF	124.70 ± 4.94a	78.53 ± 1.72a	35.53 ± 1.88a,c	108.85 ± 4.60c	129.92 ± 4.31	35.37 ± 1.93c
CB	123.77 ± 5.36a	77.97 ± 2.67a	28.80 ± 1.02b	84.07 ± 3.14a,b	141.91 ± 4.68	27.63 ± 0.87a,b
Hp H UAE	126.77 ± 2.86a	94.47 ± 2.68aa,b,c	29.83 ± 1.08b	76.54 ± 4.67a,b	109.01 ± 3.11a,b,c	23.99 ± 1.18a,b
Hp H MH	99.00 ± 2.16b,c	75.53 ± 2.62a	35.23 ± 0.84a,c	90.03 ± 3.56a,b	125.60 ± 3.69c	24.12 ± 0.86a,b
Sg L UAE	125.77 ± 4.34a	90.53 ± 2.70a	30.37 ± 1.21b	82.02 ± 3.27a,b	117.92 ± 3.77c	38.34 ± 1.46c
Sg L MH	133.27 ± 3.68a	110.73 ± 4.45a,b,c	26.13 ± 1.18b	104.07 ± 3.77c	145.99 ± 3.02b	29.67 ± 0.93a,b
To F UAE	123.93 ± 3.91a	79.80 ± 2.53a	30.37 ± 1.62b	105.33 ± 3.08c	141.37 ± 5.91	28.73 ± 1.39a,b
To F MH	112.83 ± 2.45	81.70 ± 3.85a	31.97 ± 0.66a	92.06 ± 3.19b	129.02 ± 4.18	41.57 ± 1.52b,c
To L UAE	109.60 ± 4.91	92.30 ± 4.66a,b,c	27.73 ± 1.11b	83.99 ± 3.77a,b	128.29 ± 4.49	33.70 ± 2.00c
To L MH	98.10 ± 2.50b,c	65.73 ± 2.93a	27.33 ± 0.91b	95.43 ± 4.91	136.28 ± 3.30	23.76 ± 1.13a,b
Tp F UAE	99.67 ± 4.13b,c	93.73 ± 3.62a,b,c	37.10 ± 1.70a,c	91.80 ± 4.13b	115.56 ± 4.27a,c	23.08 ± 0.55a,b
Tp F MH	130.30 ± 4.21a	98.03 ± 4.11a,b,c	38.53 ± 1.75a,c	81.32 ± 2.76a,b	133.77 ± 3.15	31.18 ± 0.95a
Ur L UAE	115.70 ± 4.65	86.40 ± 3.51a	34.13 ± 1.36a,c	91.76 ± 4.69b	124.78 ± 4.53c	27.78 ± 0.92a,b
Ur L MH	121.10 ± 3.31a	81.27 ± 1.81a	33.53 ± 1.63a	86.63 ± 2.52a,b	131.07 ± 3.17	33.50 ± 1.71c
Vo R UAE	126.60 ± 4.56a	112.20 ± 5.23a,b,c	37.50 ± 1.06a,c	95.63 ± 2.45	111.30 ± 3.15a,b,c	26.90 ± 1.31a,b
Vo R MH	136.80 ± 6.78a	112.57 ± 5.33a,b,c	23.57 ± 1.07b,c	88.81 ± 3.83a,b	114.26 ± 4.84a,b,c	34.19 ± 1.79c

*(a) Statistically significant differences (p < 0.05) between the control group (C) and the botanical extracts. (b) Statistically significant differences (p < 0.05) between the formulation (CF) and the botanical extracts. (c) Statistically significant differences (p < 0.05) between commercial biostimulant (CB) and the botanical extracts. UAE, ultrasound-assisted extraction; MH, mechanical homogenization; Hp H, Hypericum perforatum L. (St. John's wort, herb); Sg L, Solidago gigantea Ait. (giant goldenrod, leaf); To F, To L, Taraxacum officinale (L.) Weber ex F.H. Wigg (common dandelion, flower, leaf); Tp F, Trifolium pratense L. (red clover, flower); Ur L, Urtica dioica L. (nettle, leaf); Vo R, Valeriana officinalis L. (valerian, root)*.

### Total Phenolic Compounds and Antioxidant Activity (DPPH, ABTS, and FRAP Assays)

In general, the content of total phenolic compounds (TPC) in radish (Table 4) was not intensified by the application of botanical extracts in relation to the control groups. The largest amount of TPC in the leaves of rosette, in both terms, was observed in the groups treated with Sg L MH (in the first term: 1.1% less than in C but 23.8% more than in CB; in the second term: 10.7 and 2.9% more than in C and CB) and To F UAE (in the first term: 0.1 and 25.3% more than in C and CB; in the second term: 7.2% more than in C and 0.4% less than in CB). In the roots (Table 4), the most elevated amount of TPC was noted after the application of To F MH (13.2 and 50.5% more than in C and CB), and the lowest was observed for Tp F UAE (37.2 and 16.5% less than in C and CB).

The DPPH assay revealed that the bio-products mainly lowered the antioxidant activity in the leaves but increased it in the roots (Table 5). The natural preparations based on To F (UAE) and Vo R (MH) increased the activity by 76.5 and 79.8% in comparison to C and by 147 and 152% in comparison to CB in the leaves collected during the first term. The lowest activity was caused by the application of To L MH and Tp F MH (35.3 and 33.6% less than in C and 9.4 and 7.1% less than in CB). In the leaves from the second term of the plant collection, the extract Sg L UAE enhanced the activity by 28.6 and 51.8% in comparison to C and CB, while To F MH and Ur L MH diminished this activity by 40.8 and 39.8% when compared with C and by 30.1 and 28.9% when compared with CB. The antioxidant activity assessed in the roots was mostly increased after the foliar spraying with To F MH (158 and 170% more than in C and CB) but decreased with Sg L UAE (26.7 and 23.3% less than in C and CB) and To L UAE (33.3 and 30.2% less than in C and CB).

**Table 5:** The effect of the foliar application of the botanical extracts on the antioxidant activities (DPPH assay, ABTS assay, FRAP assay) (*N* = 4, mean ± SD) of radish leaves (after second spraying and after harvest) and roots (after harvest).

**Group**	**DPPH assay**			**ABTS assay**			**FRAP assay**		
	**Leaves of rosette after 2nd spraying**	**Leaves of rosette after harvest**	**Roots after harvest**	**Leaves of rosette after 2nd spraying**	**Leaves of rosette after harvest**	**Roots after harvest**	**Leaves of rosette after 2nd spraying**	**Leaves of rosette after harvest**	**Roots after harvest**
	**μM Trolox·g**^****–1****^ **FW**								
C	1.19 ± 0.04c	0.98 ± 0.08b	0.45 ± 0.04	10.44 ± 0.49b,c	17.90 ± 0.74b,c	2.32 ± 0.21b,c	2.64 ± 0.12b	3.55 ± 0.18	3.30 ± 0.32
CF	1.09 ± 0.10c	0.61 ± 0.07a,c	0.46 ± 0.07	8.02 ± 0.40a,c	4.97 ± 0.34a	4.81 ± 0.48a	4.23 ± 0.46a,c	3.18 ± 0.19	2.94 ± 0.16
CB	0.85 ± 0.07a,b	0.83 ± 0.05 b	0.43 ± 0.10	3.83 ± 0.27a,b	4.27 ± 0.19a	4.56 ± 0.29a	3.37 ± 0.22 b	3.49 ± 0.19	2.96 ± 0.21
Hp H UAE	0.95 ± 0.02a	0.63 ± 0.06a	0.69 ± 0.04a,b,c	4.59 ± 0.27a,b	9.99 ± 0.48a,b,c	4.05 ± 0.38a	2.49 ± 0.22 b,c	3.72 ± 0.20	2.82 ± 0.24
Hp H MH	0.93 ± 0.04a	0.61 ± 0.05a,c	0.76 ± 0.05a,b,c	4.56 ± 0.32a,b	9.78 ± 0.54a,b,c	3.08 ± 0.31 b	3.13 ± 0.22 b	3.93 ± 0.37 b	1.86 ± 0.16a,b,c
Sg L UAE	0.91 ± 0.04a	1.26 ± 0.05a,b,c	0.33 ± 0.02	3.58 ± 0.22a,b	12.92 ± 0.80a,b,c	4.68 ± 0.50a	2.64 ± 0.26 b	6.17 ± 0.38a,b,c	3.44 ± 0.28
Sg L MH	1.68 ± 0.10a,b,c	0.93 ± 0.10 b	0.80 ± 0.04a,b,c	3.80 ± 0.43a,b	7.25 ± 0.31a,b,c	3.48 ± 0.49	4.05 ± 0.35a	2.88 ± 0.18	2.80 ± 0.19
To F UAE	2.10 ± 0.07a,b,c	0.85 ± 0.04 b	0.80 ± 0.09a,b,c	4.43 ± 0.31a,b	8.02 ± 0.35a,b,c	2.90 ± 0.20a,c	3.58 ± 0.25a	2.96 ± 0.20	3.04 ± 0.31
To F MH	0.98 ± 0.06	0.58 ± 0.06a,c	1.16 ± 0.08a,b,c	3.56 ± 0.34a,b	6.14 ± 0.40a	4.46 ± 0.45a	3.53 ± 0.20a	3.03 ± 0.23	3.07 ± 0.15
To L UAE	1.06 ± 0.10	1.07 ± 0.09 b,c	0.30 ± 0.09	4.53 ± 0.48a,b	8.91 ± 0.45a,b,c	5.05 ± 0.48a	2.66 ± 0.16 b	3.74 ± 0.21	2.78 ± 0.22
To L MH	0.77 ± 0.09a,b	0.98 ± 0.08 b	0.90 ± 0.07a,b,c	5.67 ± 0.46a,b,c	11.30 ± 0.48a,b,c	2.98 ± 0.42 b,c	2.84 ± 0.14 b	4.23 ± 0.23 b,c	2.40 ± 0.13a
Tp F UAE	1.01 ± 0.09	1.11 ± 0.09 b,c	0.56 ± 0.07	6.46 ± 0.51a,b,c	14.00 ± 0.86a,b,c	3.10 ± 0.43 b	2.98 ± 0.28 b	3.19 ± 0.15	2.85 ± 0.30
Tp F MH	0.79 ± 0.06a,b	0.70 ± 0.05a	1.04 ± 0.06a,b,c	6.83 ± 0.40a,c	5.85 ± 0.46a	2.88 ± 0.42 b,c	2.63 ± 0.19 b	3.81 ± 0.27	2.65 ± 0.23
Ur L UAE	1.78 ± 0.04a,b,c	0.64 ± 0.07a	0.53 ± 0.06	5.37 ± 0.46a,b,c	4.76 ± 0.42a	4.66 ± 0.29a	3.19 ± 0.26 b	3.39 ± 0.19	2.39 ± 0.16a
Ur L MH	0.83 ± 0.08a,b	0.59 ± 0.07a,c	1.00 ± 0.09a,b,c	5.07 ± 0.67a,b	5.51 ± 0.49a	4.21 ± 0.32a	3.13 ± 0.19 b	4.45 ± 0.23a,b,c	2.33 ± 0.14a
Vo R UAE	1.07 ± 0.07c	0.94 ± 0.08 b	0.64 ± 0.04c	6.08 ± 0.31a,b,c	13.16 ± 0.71a,b,c	3.55 ± 0.49	2.48 ± 0.19 b,c	3.80 ± 0.23	2.48 ± 0.21
Vo R MH	2.14 ± 0.09a,b,c	0.76 ± 0.08a	0.37 ± 0.06	7.83 ± 0.44a,c	5.96 ± 0.36a	3.89 ± 0.37a	3.40 ± 0.31a,c	4.56 ± 0.24a,b,c	3.22 ± 0.32

*(a) Statistically significant differences (p < 0.05) between the control group (C) and the botanical extracts. (b) Statistically significant differences (p < 0.05) between the formulation (CF) and the botanical extracts. (c) Statistically significant differences (p < 0.05) between commercial biostimulant (CB) and the botanical extracts. UAE, ultrasound-assisted extraction; MH, mechanical homogenization; Hp H, Hypericum perforatum L. (St. John's wort, herb); Sg L, Solidago gigantea Ait. (giant goldenrod, leaf); To F, To L, Taraxacum officinale (L.) Weber ex F.H. Wigg (common dandelion, flower, leaf); Tp F, Trifolium pratense L. (red clover, flower); Ur L, Urtica dioica L. (nettle, leaf); Vo R, Valeriana officinalis L. (valerian, root)*.

The antioxidant activity measured with the ABTS assay in the aboveground parts (in both terms) was not increased after the application of all examined bio-products, whereas, in the roots, in all cases, the activity was significantly higher with reference to the control group (Table 5). In the leaves collected in the first term, the most elevated activity was found after the use of Vo R MH (25% less than in C and 104% more than in CB), and the least was found for To F MH (65.9 and 7% less than in C and CB) and Sg L UAE (65.7 and 6.5% less than in C and CB). In the second term of the samples collection, the highest level was noted for Tp F UAE (21.8% less than in C and 228% more than in CB), and the lowest was found for Ur L UAE (73.4% less than in C and 11.5% more than in CB). The foliar spraying with To L UAE increased the antioxidant activity the most (178 and 10.7% more than in C and CB), while Tp F MH showed the least impact (24.1% more than in C and 36.8% less than in CB).

The opposite trend was observed in the case of antioxidant activity measured with the FRAP assay. In general, this activity was increased in leaves of rosette but decreased in the roots (Table 5). For example, the highest activity was noted in the group treated with Sg L MH (53.4 and 20.2% more than in C and CB), and the lowest was observed for Hp H UAE (5.7 and 26.1% less than in C and CB) and Vo R UAE (6.1 and 26.4% less than in C and CB) in the leaves from the first collection, whereas for the aboveground parts from the second term, Sg L UAE enhanced the activity (73.8 and 76.8% more than in C and CB), while Sg L MH reduced it (18.9 and 17.5% less than in C and CB). In the roots, the formulation based on Sg L (UAE) increased the activity (4.2 and 16.2% more than in C and CB), while the one based on Hp H (MH) decreased it (43.6 and 37.2% less than in C and CB).

### Nitrates

In most instances, the application of the obtained bio-products resulted in a higher content of nitrates in all parts of the model plant (Table 6). The foliar spraying with Vo R MH increased the amount of examined ions in the leaves of rosette collected in the first term (57.5 and 39% more than in C and CB), while Vo R UAE and Ur L UAE (21.9 and 31.1% less than in C and CB for both extracts) decreased its content. In the harvested leaves of rosette and roots, the highest amount was observed in the groups treated with Ur L UAE (in leaves: 52.7 and 51.1% more than in C and CB; in the roots: 73.7 and 125% more than in C and CB), and the lowest was found for Vo R UAE and To F UAE in both leaves (20.3 and 21.1% less than in C and CB for both extracts) and roots (20.2% less than in C and 3.2% more than in CB; 17.5% less than in C and 6.8% more than in CB). Notably, the amount of nitrates in radish was lower than the maximum allowable levels established for vegetables, e.g., fresh spinach (3,500 mg·kg^−1^), lettuce (3,000–5,000 mg·kg^−1^), iceberg lettuce (2,000–2,500 mg·kg^−1^), and wild rocket (6,000–7,000 mg·kg^−1^) (European Communities, 2006).

**Table 6:** The effect of the foliar application of the botanical extracts on the nitrates content (*N* = 3, mean ± SD) of leaves of rosette (after second spraying and after harvest) and roots (after harvest).

**Group**	**Leaves of rosette after 2nd spraying**	**Leaves of rosette after harvest**	**Roots after harvest**
	**mg·kg**^****–1****^ **FW**		
C	1,177.15 ± 190.05	1,746.36 ± 139.59	414.11 ± 44.66
CF	664.27 ± 85.93c	1,181.64 ± 180.38	315.39 ± 23.00
CB	1,334.60 ± 106.97b	1,763.92 ± 197.94	319.99 ± 30.41
Hp H UAE	1,510.87 ± 186.91b	2,236.21 ± 165.57b	424.66 ± 32.47
Hp H MH	1,507.07 ± 233.45b	2,132.94 ± 270.27b	533.84 ± 54.92b,c
Sg L UAE	1,255.07 ± 111.46	1,594.48 ± 173.79	413.09 ± 46.42
Sg L MH	1,704.77 ± 168.77b	1,562.54 ± 239.96	366.48 ± 38.78
To F UAE	1,510.21 ± 119.35b	1,391.79 ± 175.11	341.82 ± 43.19
To F MH	1,471.21 ± 167.65b	2,046.52 ± 245.82b	505.01 ± 66.71b,c
To L UAE	1,268.90 ± 166.77	2,096.39 ± 307.57b	502.94 ± 43.35b,c
To L MH	1,744.36 ± 166.43b	1,572.90 ± 160.38	434.47 ± 43.31
Tp F UAE	1,625.58 ± 199.79b	2,653.45 ± 193.48a,b,c	410.24 ± 30.35
Tp F MH	1,674.76 ± 243.29b	2,202.20 ± 180.58b	455.91 ± 43.67
Ur L UAE	919.44 ± 85.57	2,666.06 ± 336.20a,b,c	719.16 ± 75.47a,b,c
Ur L MH	1,502.30 ± 134.73b	2,043.16 ± 243.12b	386.01 ± 44.79
Vo R UAE	919.02 ± 86.86	1,391.77 ± 108.09	330.25 ± 26.94
Vo R MH	1,854.43 ± 289.14a,b	1,455.10 ± 120.74	429.47 ± 40.77

*(a) Statistically significant differences (p < 0.05) between the control group (C) and the botanical extracts. (b) Statistically significant differences (p < 0.05) between the formulation (CF) and the botanical extracts. (c) Statistically significant differences (p < 0.05) between commercial biostimulant (CB) and the botanical extracts. UAE, ultrasound-assisted extraction; MH, mechanical homogenization; Hp H, Hypericum perforatum L. (St. John's wort, herb); Sg L, Solidago gigantea Ait. (giant goldenrod, leaf); To F, To L, Taraxacum officinale (L.) Weber ex F.H. Wigg (common dandelion, flower, leaf); Tp F, Trifolium pratense L. (red clover, flower); Ur L, Urtica dioica L. (nettle, leaf); Vo R, Valeriana officinalis L. (valerian, root)*.

### Macroelements, Microelements, and Heavy Metals

In the case of radish leaves, generally, P, K, Mg, and S were increased in the biomass, and N and Ca decreased. The highest content of P was observed in the group treated with To F MH and Vo R MH (higher by 25.8% than in the control group–C), and the highest content of K was observed in the groups treated with Ur L UAE (higher by 29.7% than in C), Vo R MH (higher by 27.2% than in C), and Hp H UAE (higher by 24.5% than in C). The greatest amount of Mg was found in the group treated with Sg L UAE (higher by only 3.1% than in C), and Vo R MH (higher by only 1.5% than in C), and the highest content of S was found in the groups treated with To L MH (higher by 18.4% than in C), Vo R UAE (higher by 13.2% than in C), and Ur L UAE (higher by 11.4% than in C). The extracts produced from nettle (*Urtica dioica* L.) and valerian (*Valeriana officinalis* L.) ensured an increase in the content of macronutrients. The amount of nitrogen was comparable in all tested groups, but, for botanical extracts, it was slightly lower than in the control group (C), from 2.7% for Ur L UAE to 8.1% for Sg L UAE. The same was observed for the content of Ca in the leaves. In all experimental groups, this content was lower than in the control group (from 18.5% for Tp F UAE to 2.5% for To L MH) (Table 7A).

**Table 7A:** The effect of the foliar application of the botanical extracts on the content of macroelements (*N* = 3, mean ± SD) in radish leaves of rosette and roots (after harvest).

**Group**	**N**		**P**		**K**		**Ca**		**Mg**		**S**	
	**Leaves**	**Roots**	**Leaves**	**Roots**	**Leaves**	**Roots**	**Leaves**	**Roots**	**Leaves**	**Roots**	**Leaves**	**Roots**
	**g·kg**^****−1****^ **DW**											
C	37.00 ± 1.85	32.75 ± 1.64	1.94 ± 0.10	4.41 ± 0.22	32.22 ± 1.61b,c	80.44 ± 4.02	11.13 ± 0.56	9.63 ± 0.48b,c	2.59 ± 0.13	2.13 ± 0.11	7.19 ± 0.36	7.05 ± 0.35
CF	34.50 ± 1.73	31.75 ± 1.59	2.19 ± 0.11	4.47 ± 0.22c	41.47 ± 2.07a	71.58 ± 3.58	10.08 ± 0.50	7.68 ± 0.38a	2.56 ± 0.13	1.86 ± 0.09	8.30 ± 0.42	6.15 ± 0.31
CB	35.00 ± 1.75	33.25 ± 1.66	2.16 ± 0.11	3.72 ± 0.19b	38.39 ± 1.92a	77.05 ± 3.85	10.49 ± 0.52	7.71 ± 0.39a	2.71 ± 0.14	1.90 ± 0.10	7.98 ± 0.40	6.20 ± 0.31
Hp H UAE	35.75 ± 1.79	34.63 ± 1.73	2.13 ± 0.11	5.16 ± 0.26a,c	40.10 ± 2.01a	81.83 ± 4.09	10.44 ± 0.52	7.55 ± 0.38a	2.61 ± 0.13	1.84 ± 0.09	8.00 ± 0.40	7.08 ± 0.35
Hp H MH	35.25 ± 1.76	34.00 ± 1.70	1.88 ± 0.09	5.16 ± 0.26a,c	36.72 ± 1.84	79.36 ± 3.97	10.60 ± 0.53	8.29 ± 0.41a	2.58 ± 0.13	2.09 ± 0.10	7.29 ± 0.36	6.58 ± 0.33
Sg L UAE	34.00 ± 1.70	32.13 ± 1.61	2.06 ± 0.10	5.28 ± 0.26a,b,c	37.76 ± 1.89	83.34 ± 4.17b	9.88 ± 0.49	7.87 ± 0.39a	2.67 ± 0.13	2.02 ± 0.10	7.53 ± 0.38	7.04 ± 0.35
Sg L MH	34.75 ± 1.74	33.50 ± 1.68	1.97 ± 0.10	4.59 ± 0.23c	35.06 ± 1.75b	74.92 ± 3.75	10.77 ± 0.54	7.59 ± 0.38a	2.58 ± 0.13	1.82 ± 0.09	7.61 ± 0.38	6.33 ± 0.32
To F UAE	35.25 ± 1.76	33.75 ± 1.69	2.16 ± 0.11	5.25 ± 0.26a,b,c	38.26 ± 1.91a	80.14 ± 4.01	9.70 ± 0.48	8.30 ± 0.42a	2.45 ± 0.12	2.16 ± 0.11	7.06 ± 0.35b	6.50 ± 0.33
To F MH	34.75 ± 1.74	33.75 ± 1.69	2.44 ± 0.12a	4.72 ± 0.24c	33.69 ± 1.68b	78.30 ± 3.91	10.22 ± 0.51	7.91 ± 0.40a	2.48 ± 0.12	1.95 ± 0.10	7.13 ± 0.36b	6.50 ± 0.33
To L UAE	35.00 ± 1.75	32.88 ± 1.64	2.09 ± 0.10	4.75 ± 0.24c	36.69 ± 1.83	84.30 ± 4.21b	10.22 ± 0.51	7.63 ± 0.38a	2.65 ± 0.13	1.85 ± 0.09	7.24 ± 0.36	7.03 ± 0.35
To L MH	35.25 ± 1.76	33.50 ± 1.68	1.28 ± 0.06a,b,c	4.75 ± 0.24c	37.78 ± 1.89	81.63 ± 4.08	10.85 ± 0.54	8.40 ± 0.42	2.52 ± 0.13	2.02 ± 0.10	8.51 ± 0.43a	6.68 ± 0.33
Tp F UAE	35.75 ± 1.79	34.00 ± 1.70	2.13 ± 0.11	5.09 ± 0.25c	39.16 ± 1.96a	82.03 ± 4.10	9.02 ± 0.45a	8.04 ± 0.40a	2.58 ± 0.13	2.01 ± 0.10	7.50 ± 0.38	6.86 ± 0.34
Tp F MH	35.50 ± 1.78	34.00 ± 1.70	2.00 ± 0.10	2.13 ± 0.11a,b,c	39.46 ± 1.97a	37.24 ± 1.86a,b,c	10.70 ± 0.54	10.09 ± 0.50b,c	2.56 ± 0.13	2.48 ± 0.12a,b,c	7.40 ± 0.37	6.71 ± 0.34
Ur L UAE	36.00 ± 1.80	33.50 ± 1.68	2.03 ± 0.10	5.31 ± 0.27a,b,c	41.78 ± 2.09a	82.45 ± 4.12	10.01 ± 0.50	7.96 ± 0.40a	2.52 ± 0.13	2.01 ± 0.10	8.01 ± 0.40	6.70 ± 0.34
Ur L MH	35.00 ± 1.75	33.75 ± 1.69	2.03 ± 0.10	2.06 ± 0.10a,b,c	37.43 ± 1.87	34.40 ± 1.72a,b,c	10.32 ± 0.52	10.40 ± 0.52b,c	2.53 ± 0.13	2.48 ± 0.12a,b,c	7.41 ± 0.37	6.74 ± 0.34
Vo R UAE	34.50 ± 1.73	32.75 ± 1.64	2.16 ± 0.11	5.06 ± 0.25c	37.65 ± 1.88	83.81 ± 4.19b	10.15 ± 0.51	8.26 ± 0.41a	2.34 ± 0.12	1.98 ± 0.10	8.14 ± 0.41	6.90 ± 0.35
Vo R MH	34.75 ± 1.74	34.25 ± 1.71	2.44 ± 0.12a	5.16 ± 0.26a,c	40.98 ± 2.05a	85.91 ± 4.30b	10.49 ± 0.52	12.08 ± 0.60a,b,c	2.63 ± 0.13	3.73 ± 0.19a,b,c	7.09 ± 0.35b	6.81 ± 0.34

*(a)Statistically significant differences (p < 0.05) between the control group (C) and the botanical extracts. (b) Statistically significant differences (p < 0.05) between the formulation (CF) and the botanical extracts. (c) Statistically significant differences (p < 0.05) between commercial biostimulant (CB) and the botanical extracts. UAE, ultrasound-assisted extraction; MH, mechanical homogenization; Hp H, Hypericum perforatum L. (St. John's wort, herb); Sg L, Solidago gigantea Ait. (giant goldenrod, leaf); To F, To L, Taraxacum officinale (L.) Weber ex F.H. Wigg (common dandelion, flower, leaf); Tp F, Trifolium pratense L. (red clover, flower); Ur L, Urtica dioica L. (nettle, leaf); Vo R, Valeriana officinalis L. (valerian, root)*.

The results showed that the tested macroelements were better accumulated in the roots, as more statistically significant differences were demonstrated (Table 7A). The content of N was highest in the groups treated with Hp H UAE (by 5.7% higher than in C), P for Ur L UAE (by 20.4% higher), K for Vo R MH (by 6.8% higher), Ca for Vo R MH (by 25.4% higher), and Mg for Vo R MH (by 75.1% higher). The content of S was comparable in all experimental groups but was slightly lower than that in the control group. This analysis showed that the extract from valerian (*Valeriana officinalis* L.) was the most effective in increasing the macronutrient content in the roots. In most cases, the production method did not affect the macronutrient content in the tested biomass.

The examined botanical extracts, applied foliarly, increased the level of some microelements (Table 7B), especially Zn in the leaves (in all experimental groups). In other cases, the micronutrient composition of the leaves was comparable to that in the control group. Higher contents of the examined elements were reported for Fe (for To L UAE, higher by 24.3% than in the control group) and Zn (for Sg L UAE, higher by 24.8% than in the control group). As with macronutrients, the micronutrients (Fe, Cu, Zn, Mn) were more strongly accumulated in the roots (Table 7B). Again, the most significant results were obtained for the extract from valerian, whose Fe, Cu, and Mn contents were, respectively, 27.9, 45.5, and 29.3% higher than those in the control group.

**Table 7B:** The effect of the foliar application of the botanical extracts on the content of microelements and toxic elements (*N* = 3, mean ± SD) in radish leaves of rosette and roots (after harvest).

**Group**	**Fe**		**Cu**		**Zn**		**Mn**		**Ni**		**Cd**		**Pb**	
	**Leaves**	**Roots**	**Leaves**	**Roots**	**Leaves**	**Roots**	**Leaves**	**Roots**	**Leaves**	**Roots**	**Leaves**	**Roots**	**Leaves**	**Roots**
	**mg·kg**^****−1****^ **DW**													
C	1,503.75 ± 75.19b	426.75 ± 21.34b	5.68 ± 0.28	1.54 ± 0.08b	37.49 ± 1.87	45.16 ± 2.26b	85.13 ± 4.26b	25.63 ± 1.28c	10.44 ± 0.52c	5.33 ± 0.27b	1.66 ± 0.08	0.66 ± 0.03b,c	10.05 ± 0.50	5.49 ± 0.27b,c
CF	1,100.00 ± 55.00a,c	337.88 ± 16.89a	6.08 ± 0.30	1.95 ± 0.10a,c	41.71 ± 2.09	30.19 ± 1.51a,c	72.00 ± 3.60a	28.00 ± 1.40	10.05 ± 0.50	3.94 ± 0.20a,c	1.58 ± 0.08	0.51 ± 0.03a	9.17 ± 0.46	3.21 ± 0.16a
CB	1,412.50 ± 70.63b	380.88 ± 19.04	6.26 ± 0.31	1.41 ± 0.07b	41.03 ± 2.05	43.34 ± 2.17b	83.00 ± 4.15	30.50 ± 1.53a	8.76 ± 0.44a	5.63 ± 0.28b	1.61 ± 0.08	0.50 ± 0.03a	9.55 ± 0.48	3.61 ± 0.18a
Hp H UAE	1,631.25 ± 81.56b,c	446.63 ± 22.33b	4.46 ± 0.22a,b,c	1.53 ± 0.08b	43.95 ± 2.20a	43.93 ± 2.20b	83.75 ± 4.19	23.25 ± 1.16b,c	8.40 ± 0.42a,b	6.41 ± 0.32a,b	1.60 ± 0.08	0.65 ± 0.03b,c	10.66 ± 0.53b	5.04 ± 0.25b,c
Hp H MH	1,298.75 ± 64.94	519.00 ± 25.95a,b,c	4.50 ± 0.23a,b,c	1.61 ± 0.08b	43.20 ± 2.16	43.54 ± 2.18b	79.13 ± 3.96	33.50 ± 1.68a,b	8.69 ± 0.43a	7.33 ± 0.37a,b,c	1.68 ± 0.08	0.58 ± 0.03	9.28 ± 0.46	4.39 ± 0.22a,b,c
Sg L UAE	1,567.50 ± 78.38b	420.38 ± 21.02b	5.50 ± 0.28	1.69 ± 0.08b,c	46.80 ± 2.34a	45.98 ± 2.30b	86.50 ± 4.33b	22.25 ± 1.11b,c	11.11 ± 0.56c	5.03 ± 0.25b	1.54 ± 0.08	0.66 ± 0.03b,c	9.93 ± 0.50	5.03 ± 0.25b,c
Sg L MH	1,107.50 ± 55.38a,c	313.88 ± 15.69a	5.26 ± 0.26b,c	1.81 ± 0.09a,c	40.55 ± 2.03	39.84 ± 1.99b	77.25 ± 3.86	26.75 ± 1.34	8.59 ± 0.43a,b	5.00 ± 0.25b	1.68 ± 0.08	0.53 ± 0.03a	9.34 ± 0.47	3.79 ± 0.19a
To F UAE	1380.00 ± 69.00b	494.38 ± 24.72b,c	4.95 ± 0.25b,c	1.89 ± 0.09a,c	40.01 ± 2.00	46.60 ± 2.33b	86.50 ± 4.33b	32.25 ± 1.61a	10.24 ± 0.51c	5.78 ± 0.29b	1.55 ± 0.08	0.68 ± 0.03b,c	9.51 ± 0.48	5.14 ± 0.26b,c
To F MH	1,496.25 ± 74.81b	462.38 ± 23.12b,c	5.29 ± 0.26c	1.70 ± 0.09c	37.74 ± 1.89	41.86 ± 2.09b	81.88 ± 4.09	31.25 ± 1.56a	10.40 ± 0.52c	11.04 ± 0.55a,b,c	1.59 ± 0.08	0.58 ± 0.03	9.41 ± 0.47	3.68 ± 0.18a
To L UAE	1,868.75 ± 93.44a,b,c	543.25 ± 27.16a,b,c	5.93 ± 0.30	1.45 ± 0.07b	41.98 ± 2.10	43.61 ± 2.18b	86.75 ± 4.34b	23.88 ± 1.19c	9.19 ± 0.46	6.00 ± 0.30b	1.54 ± 0.08	0.65 ± 0.03b,c	10.42 ± 0.52	5.10 ± 0.26b,c
To L MH	1,162.50 ± 58.13a,c	379.38 ± 18.97	4.88 ± 0.24a,b,c	1.54 ± 0.08b	43.00 ± 2.15	42.10 ± 2.11b	88.38 ± 4.42b	31.25 ± 1.56a	8.51 ± 0.43a,b	6.84 ± 0.34a,b,c	1.65 ± 0.08	0.59 ± 0.03	9.93 ± 0.50	4.06 ± 0.20a,b
Tp F UAE	1,318.75 ± 65.94b	433.00 ± 21.65b	4.66 ± 0.23a,b,c	1.38 ± 0.07b	39.18 ± 1.96	42.35 ± 2.12b	82.00 ± 4.10	24.00 ± 1.20c	8.49 ± 0.42a,b	5.54 ± 0.28b	1.63 ± 0.08	0.68 ± 0.03b,c	9.85 ± 0.49	4.84 ± 0.24b,c
Tp F MH	1,360.00 ± 68.00b	365.88 ± 18.29	4.51 ± 0.23a,b,c	1.65 ± 0.08b	40.55 ± 2.03	40.68 ± 2.03b	84.38 ± 4.22	29.13 ± 1.46	8.03 ± 0.40a,b	5.73 ± 0.29b	1.68 ± 0.08	0.55 ± 0.03a	9.58 ± 0.48	3.95 ± 0.20a,b
Ur L UAE	1,185.00 ± 59.25a,c	517.13 ± 25.86a,b,c	4.85 ± 0.24a,b,c	1.50 ± 0.08b	38.38 ± 1.92	44.41 ± 2.22b	74.50 ± 3.73	30.63 ± 1.53a	8.81 ± 0.44a	6.44 ± 0.32a,b	1.58 ± 0.08	0.63 ± 0.03b,c	9.46 ± 0.47	5.11 ± 0.26b,c
Ur L MH	1,156.25 ± 57.81a,c	390.38 ± 19.52	4.69 ± 0.23a,b,c	1.54 ± 0.08b	37.71 ± 1.89	41.95 ± 2.10b	72.63 ± 3.63a	29.75 ± 1.49	8.63 ± 0.43a,b	9.34 ± 0.47a,b,c	1.61 ± 0.08	0.54 ± 0.03a	8.61 ± 0.43	4.39 ± 0.22a,b,c
Vo R UAE	1,582.50 ± 79.13b	541.75 ± 27.09a,b,c	5.73 ± 0.29	1.81 ± 0.09a,c	41.88 ± 2.09	45.41 ± 2.27b	81.75 ± 4.09	27.75 ± 1.39	9.76 ± 0.49	5.65 ± 0.28b	1.50 ± 0.08	0.71 ± 0.04b,c	9.84 ± 0.49	5.60 ± 0.28b,c
Vo R MH	1,201.25 ± 60.06a	545.88 ± 27.29a,b,c	4.35 ± 0.22a,b,c	2.24 ± 0.11a,b,c	41.55 ± 2.08	46.11 ± 2.31b	82.63 ± 4.13	33.13 ± 1.66a,b	8.09 ± 0.40a,b	7.24 ± 0.36a,b,c	1.61 ± 0.08	0.64 ± 0.03b,c	9.31 ± 0.47	4.11 ± 0.21a,b

*(a) Statistically significant differences (p < 0.05) between the control group (C) and the botanical extracts. (b) Statistically significant differences (p < 0.05) between the formulation (CF) and the botanical extracts. (c) Statistically significant differences (p < 0.05) between commercial biostimulant (CB) and the botanical extracts. UAE, ultrasound-assisted extraction; MH, mechanical homogenization; Hp H, Hypericum perforatum L. (St. John's wort, herb); Sg L, Solidago gigantea Ait. (giant goldenrod, leaf); To F, To L, Taraxacum officinale (L.) Weber ex F.H. Wigg (common dandelion, flower, leaf); Tp F, Trifolium pratense L. (red clover, flower); Ur L, Urtica dioica L. (nettle, leaf); Vo R, Valeriana officinalis L. (valerian, root)*.

The content of heavy metals (Ni, Cd, and Pb) was generally lower in the leaves and roots of radish in the experimental groups compared with the control, with the exception of the Ni content in the roots, which was higher than in the control group (Table 7B). The obtained levels of Cd and Pb were comparable with the results presented in other studies, where the content of Cd was at a level between 1 and 2.8 mg·kg^−1^, and Pb was found at 6.4–10.98 mg·kg^−1^ (Varalakshmi and Ganeshamurthy, 2008; Veissi et al., 2015; Rolli et al., 2016). The tolerable levels in agricultural crops are 3 mg·kg^−1^ for Cd and 10 mg·kg^−1^ for Pb (Rolli et al., 2016), whereas, according to the EC Commission Regulations, the threshold content is 0.05–0.2 and 0.1–0.3 mg·kg^−1^, respectively [(EC) Commission Regulation No. 2006.364.5 of 19 December 2006, setting maximum levels for certain contaminants in foodstuffs].

### Steam Volatile Compounds

GC–MS analyses of the distilled essential oil from the radish leaves revealed 38 peaks, which were found to represent steam volatile compounds (SVCs) (Supplementary Table 1). The following components constituted the main parts of the SVCs: limonene, myrcene, phytol, and cis-β-ocimene. However, p-Cymene, β-pinene, ionone epoxide, nonanal, hexahydrofarnesyl acetone, and hex-(2*E*)-enal were present in smaller quantities. In most cases, the examined extracts did not increase the content of limonene, except for Vo R UAE (11.3 and 9.5% more than in C and CB), To L UAE (4.8 and 3.1% more than in C and CB), and Hp H UAE (3.2 and 1.5% more than in C and CB). The lowest increment was observed after treatment with Tp F MH (25.4 and 26.6% less than in C and CB). The amount of myrcene was mostly lower than that in the control group (C), but an increase was noted after the application of the aforementioned bio-products, Vo R UAE (7.4 and 25.9% more than in C and CB), To L UAE (2.3 and 19.9% more than in C and CB), and Hp H UAE (1.1 and 18.5% more than in C and CB). The foliar spraying with Tp F MH decreased the content of myrcene (33.4 and 21.9% less than in C and CB). A great diversity was observed among the examined groups in terms of phytol content. Extracts based on e.g., Tp F MH significantly increased the phytol amount (329 and 468% more than in C and CB), along with Sg L UAE (175 and 234% more than in C and CB), To L MH (132 and 207% more than in C and CB), Sg L MH (120 and 192% more than in C and CB), and To F UAE (110 and 178% more than in C and CB). On the other hand, extracts obtained from Vo R UAE (64.4 and 52.9% less than in C and CB) and To L UAE (56.9 and 42.9% less than in C and CB) significantly reduced the phytol amount. In most cases, the content of cis-β-ocimene was also decreased compared with the control group (C). The highest increment was noted in the groups sprayed with Hp H UAE (7.3 and 21% more than in C and CB) and Vo R UAE (3.4 and 16.6% more than in C and CB), and the lowest was found with Tp F MH (31.4 and 22.6% less than in C and CB).

### Fatty Acids

The largest percentage share of fatty acids (Supplementary Table 2) accounted for linolenic acid (methyl ester) and hexadecanoic acid (methyl ester), followed by 9,12-hexadecadienoic acid (methyl ester), 9*Z*-9-octadecenoic acid (ethyl ester), 11-octadecenoic acid (methyl ester), and octadecanoic acid (methyl ester). The content of linolenic acid was the highest in the groups treated with Ur L UAE (14.7 and 19.3% more than in C and CB) and Sg L MH (14.3 and 18.8% more than in C and CB) and the lowest after the application of Ur L MH (27.2 and 24.3% less than in C and CB) and Tp F MH (24.8 and 21.8% less than in C and CB). The tested botanical extracts did not increase the amount of the second acid in comparison with the control group. The highest value was achieved for Sg L MH (13.2% less than in C and 9% more than in CB), and the lowest was achieved for To F MH (34.7 and 18% less than in C and CB). The application of Sg L MH resulted in the highest amount of 9,12-hexadecadienoic acid (6.4 and 23.7% more than in C and CB) and 11-octadecenoic acid (1% less than in C and 19.3% more than in CB) in comparison to the other examined extracts, while the lowest was observed in groups treated with Ur L MH: 38.7 and 28.7% less than in C and CB for 9,12-hexadecadienoic acid and 35.1 and 21.8% less than in C and CB for 11-octadecenoic acid, respectively. On the other hand, the largest percentage share of 9*Z*-9-octadecenoic acid was noted after the foliar spraying with Ur L UAE (29.5 and 16% less than in C and CB). The greatest stimulation of octadecanoic acid content was observed in the group treated with To F UAE (39.3% less than in C and 10% more than in CB). The application of To F MH caused the smallest increase in 9*Z*-9-octadecenoic acid (53 and 43.9% less than in C and CB) and octadecanoic acid (53.3 and 15.4% less than in C and CB) content. It can be seen that, of all used extracts, To F UAE increased to the greatest extent the content of tetradecanoic acid (ethyl ester) (no change in the control group and 225% more than in CB), tetradecanoic acid (12-methyl-, methyl ester) (8.4 and 177% more than in C and CB), and pentadecanoic acid (14-methyl-, methyl ester) (4.1 and 181% more than in C and CB), while Tp F UAE had the greatest content of *Z*-9-Hexadecenoic acid (methyl ester) (3.4 and 29.5% more than in C and CB). The treatment with Tp F MH decreased the amount of tetradecanoic acid (ethyl ester) (65% less than in C and 13.7% more than in CB) and pentadecanoic acid (14-methyl-, methyl ester) (63.2 and 0.8% less than in C and CB). The lowest content of tetradecanoic acid (12-methyl-, methyl ester) was observed for Hp H MH (59% less than in C and 4.6% more than in CB), while the lowest content of *Z*-9-hexadecenoic acid (methyl ester) was observed for To L UAE (62.1 and 52.6% less than in C and CB).

### Sterols

Only two sterols were observed in the dried radish roots (Table 8). It can be seen that the botanical extracts mostly lowered the content of β-sitosterol (TMS derivative), for instance, after treatment with To L MH (8.9 and 8.4% less than in C and CB). Only the foliar application of Tp F UAE (1.9 and 2.5% more than in C and CB) and Sg L UAE (0.1 and 0.7% more than in C and CB) led to an increase of the previously mentioned content. The opposite trend was observed in the amount of campesterol (TMS derivative); in most of the cases, this content was enhanced, e.g., in the group sprayed with To L MH (15.7 and 14.5% more than in C and CB), while only two bio-products diminished it: Tp F UAE (3.3 and 4.3% less than in C and CB) and Sg L UAE (0.2 and 1.3% less than in C and CB).

**Table 8:** The effect of the foliar application of the botanical extracts on the sterols composition [the amount of a single component calculated as a percentage (%) of the whole GC-MS chromatogram area] (*N* = 3, mean ± SD) of radish roots (after harvest).

	**Campesterol, TMS derivative**	**β-Sitosterol, TMS derivative**
RT, min	26.592	27.975
RI_exp	2,683	2,784
RI_lit	2,689	2,789
C	36.17 ± 0.23	63.83 ± 0.23
CF	36.37 ± 0.07	63.63 ± 0.07
CB	36.56 ± 0.09	63.44 ± 0.09
Hp H UAE	36.93 ± 0.06a,b	63.07 ± 0.06a,b
Hp H MH	37.96 ± 0.08a,b,c	62.04 ± 0.08a,b,c
Sg L UAE	36.09 ± 0.10c	63.91 ± 0.10c
Sg L MH	36.26 ± 0.08	63.74 ± 0.08
To F UAE	36.34 ± 0.07	63.66 ± 0.07
To F MH	36.82 ± 0.10a	63.18 ± 0.10a
To L UAE	39.00 ± 0.04a,b,c	61.00 ± 0.04a,b,c
To L MH	41.86 ± 0.10a,b,c	58.14 ± 0.10a,b,c
Tp F UAE	34.97 ± 0.05a,b,c	65.03 ± 0.05a,b,c
Tp F MH	36.42 ± 0.15	63.58 ± 0.15
Ur L UAE	39.87 ± 0.11a,b,c	60.13 ± 0.11a,b,c
Ur L MH	37.82 ± 0.15a,b,c	62.18 ± 0.15a,b,c
Vo R UAE	37.93 ± 0.15a,b,c	62.07 ± 0.15a,b,c
Vo R MH	37.55 ± 0.23a,b,c	62.45 ± 0.23a,b,c

*(a) Statistically significant differences (p < 0.05) between the control group (C) and the botanical extracts. (b) Statistically significant differences (p < 0.05) between the formulation (CF) and the botanical extracts. (c) Statistically significant differences (p < 0.05) between commercial biostimulant (CB) and the botanical extracts. RT, retention time; RI, retention indices; RI_lit, retention indices according to NIST (The NIST Mass Spectral Search Program for the NIST/EPA/NIH EI and NIST Tandem Spectral Library, 2017); RI_exp, retention indices based on experiments; UAE, ultrasound-assisted extraction; MH, mechanical homogenization; Hp H, Hypericum perforatum L. (St. John's wort, herb); Sg L, Solidago gigantea Ait. (giant goldenrod, leaf); To F, To L, Taraxacum officinale (L.) Weber ex F.H. Wigg (common dandelion, flower, leaf); Tp F, Trifolium pratense L. (red clover, flower); Ur L, Urtica dioica L. (nettle, leaf); Vo R, Valeriana officinalis L. (valerian, root)*.

### Glucosinolates

The detected glucosinolates in the radish roots are presented in Table 9. It can be noted that the foliar treatment with the botanical extracts had a statistically significant impact on the glucosinolates composition. The chromatographic analyses revealed that the roots consisted mostly of glucoerucin and glucobrassicanapin, followed by gluconasturtiin, and then gluconapin. The content of glucoerucin was the most increased in the groups treated with Sg L UAE (9.8 and 8.5% more than in C and CB), Hp H UAE (7.2 and 5.9% more than in C and CB), Hp H MH (6.6 and 5.3% more than in C and CB), and the least with Vo R MH (8.6 and 9.8% less than in C and CB), To F UAE (8.3 and 9.4% less than in C and CB), and Ur L UAE (8.2 and 9.3% less than in C and CB). In the case of glucobrassicanapin, the highest amounts were observed in the groups treated with To L UAE (7 and 8.5% more than in C and CB) and Ur L MH (5.9 and 7.4% more than in C and CB), while the lowest with Hp H MH (1% less than in C and 0.3% more than in CB), Sg L MH (0.5% less than in C and 0.8% more than in CB), and Hp H UAE (0.5% less than in C and 0.9% more than in CB). The concentration of gluconasturtiin was the most increased after the treatment with Sg L MH (2.9 and 10.6% more than in C and CB), Vo R MH (2.2 and 9.8% more than in C and CB), and Tp F UAE (1.1 and 8.7% more than in C and CB), while the most decreased with To L UAE (10.6 and 4% less than in C and CB), Ur L MH (10.1 and 3.4% less than in C and CB), and Sg L UAE (9.8 and 3.1% less than in C and CB). The content of gluconapin was the most elevated in the groups sprayed with Sg L MH (39.2 and 18.7% more than in C and CB) and Vo R MH (38.4 and 18% more than in C and CB), whereas the utilization of To F MH (0.4 and 15.1% less than in C and CB) and Tp F MH (2.3% more than in C and 12.8% less than in CB) led to the lowest values this parameter.

**Table 9:** The effect of the foliar application of the botanical extracts on the glucosinolates content (μg·g^−1^) (*N* = 3, mean ± SD) of radish roots (after harvest).

**Group**	**Glucoerucin**	**Gluconapin**	**Progoitrin**	**Glucoraphenin**	**4-metoxyglucobrassicin**	**Glucobrassicin**	**Glucoraphanin**	**Gluconasturtiin**	**Glucobrassicanapin**
C	241.29 ± 15.27	48.69 ± 5.59	7.73 ± 0.90	21.38 ± 1.53	26.45 ± 0.79	17.39 ± 0.90	8.82 ± 0.49	135.73 ± 3.49c	218.58 ± 4.17b
CF	245.59 ± 0.81	43.28 ± 2.41c	9.00 ± 0.16	21.51 ± 0.42	25.06 ± 0.58c	17.53 ± 0.20	8.68 ± 0.33	130.81 ± 0.38	227.91 ± 0.80a,c
CB	244.30 ± 7.34	57.12 ± 2.55b	7.47 ± 0.26	22.47 ± 0.30	28.17 ± 0.38b	16.94 ± 0.40	8.47 ± 0.17	126.27 ± 0.90a	215.61 ± 0.88b
Hp H UAE	258.70 ± 4.76	60.51 ± 0.86a,c	7.61 ± 0.50	25.20 ± 0.57a,b	29.46 ± 0.49a,b	17.65 ± 0.65	8.69 ± 0.23	135.75 ± 1.80c	217.55 ± 1.08b
Hp H MH	257.29 ± 7.61	55.02 ± 2.54b	8.53 ± 0.61	20.82 ± 0.61	27.85 ± 0.17b	17.98 ± 0.29	10.80 ± 0.32a,b,c	124.66 ± 0.22a,b	216.35 ± 0.49b
Sg L UAE	265.00 ± 8.20	62.58 ± 2.81a,b	7.96 ± 0.33	22.69 ± 0.85	25.39 ± 0.41c	17.05 ± 0.47	10.29 ± 0.35a,b,c	122.40 ± 3.25a,b	227.69 ± 1.81a,c
Sg L MH	238.56 ± 3.11	67.78 ± 1.29a,b	7.37 ± 0.42	23.30 ± 0.53	25.45 ± 0.78c	17.87 ± 0.53	9.40 ± 0.50	139.62 ± 0.61b,c	217.40 ± 1.19b
To F UAE	221.23 ± 1.34	64.79 ± 1.59a,b	7.75 ± 0.39	23.13 ± 1.43	28.97 ± 0.67a,b	18.24 ± 0.85	8.95 ± 0.65	132.77 ± 0.61c	228.17 ± 1.47a,c
To F MH	234.64 ± 3.79	48.47 ± 4.62	8.43 ± 0.24	19.36 ± 0.35	26.27 ± 0.75	17.75 ± 0.85	9.74 ± 0.41	130.73 ± 0.07	218.49 ± 0.88b
To L UAE	235.09 ± 5.09	63.24 ± 3.72a,b	7.59 ± 0.31	24.12 ± 1.98	28.67 ± 0.74b	17.11 ± 0.31	9.02 ± 0.32	121.28 ± 1.41a,b	233.98 ± 3.08a,c
To L MH	249.64 ± 2.97	55.02 ± 4.85b	7.09 ± 0.63b	21.60 ± 0.20	24.37 ± 0.48c	16.66 ± 0.25	8.82 ± 0.11	129.65 ± 1.59a	224.23 ± 0.44c
Tp F UAE	239.82 ± 4.13	61.56 ± 0.88a,b	7.86 ± 0.45	22.45 ± 0.46	28.91 ± 0.64a,b	17.93 ± 0.69	9.10 ± 0.26	137.21 ± 1.05b,c	220.62 ± 1.09b
Tp F MH	231.04 ± 2.72	49.79 ± 2.01	6.89 ± 0.51b	20.66 ± 1.48	25.61 ± 0.27c	18.75 ± 0.38	9.69 ± 0.24	128.14 ± 0.38a	218.46 ± 0.26b
Ur L UAE	221.61 ± 1.23	61.99 ± 1.45a,b	8.02 ± 0.28	23.27 ± 0.48	28.44 ± 1.03b	17.60 ± 0.47	8.90 ± 0.33	132.36 ± 1.78c	224.03 ± 1.33c
Ur L MH	243.87 ± 9.45	56.67 ± 2.47b	8.03 ± 0.50	21.77 ± 0.99	25.91 ± 0.78	16.75 ± 0.31	11.06 ± 0.19a,b,c	121.96 ± 0.82a,b	231.58 ± 0.32a,c
Vo R UAE	247.13 ± 18.65	62.21 ± 2.54a,b	7.87 ± 0.48	21.46 ± 1.01	25.91 ± 0.67	17.08 ± 0.90	9.27 ± 0.24	132.03 ± 2.25	223.87 ± 2.24c
Vo R MH	220.44 ± 1.74	67.40 ± 0.92a,b	7.51 ± 0.40	22.95 ± 0.67	27.52 ± 0.34b	17.91 ± 0.47	9.67 ± 0.44	138.66 ± 0.95b,c	224.97 ± 1.35a,c

*(a) Statistically significant differences (p < 0.05) between the control group (C) and the botanical extracts. (b) Statistically significant differences (p < 0.05) between the formulation (CF) and the botanical extracts. (c) Statistically significant differences (p < 0.05) between commercial biostimulant (CB) and the botanical extracts. UAE, ultrasound-assisted extraction; MH, mechanical homogenization; Hp H, Hypericum perforatum L. (St. John's wort, herb); Sg L, Solidago gigantea Ait. (giant goldenrod, leaf); To F, To L, Taraxacum officinale (L.) Weber ex F.H. Wigg (common dandelion, flower, leaf); Tp F, Trifolium pratense L. (red clover, flower); Ur L, Urtica dioica L. (nettle, leaf); Vo R, Valeriana officinalis L. (valerian, root)*.

## Discussion

About 33% of global organic farming is located in Europe, and 6.4% of this farming is situated in Poland, where the interest in sustainable agricultural techniques and technologies is constantly growing and has been especially strong in the recent years of 1999–2013. During this period, the number of Polish producers of organic food increased from 27 to 26,499, and the crop area increased from 300 to 674,694 ha (Pylak et al., 2019). Thus, there is clearly a need to develop new, sustainable products to provide high-quality yields and support the future of organic farming (Röös et al., 2018). The lower yield (by 5–32%) in organic horticulture, in relation to conventional farming, is mostly caused by nutrient availability (mainly N and P) but is also linked to fungal or bacteria pathogens (Fess and Benedito, 2018; Pylak et al., 2019). Therefore, the application of biostimulants to increase crop productivity, soil nutrient availability, water use efficiency, plant nutrient uptake, and assimilation has emerged as a promising eco-friendly approach. These factors could contribute to a decrease in the use of fertilizers (in particular, rich in nitrogen) and an increase in crop tolerance to abiotic and biotic stresses (Bulgari et al., 2015, 2019; Paradiković et al., 2018; Drobek et al., 2019; Pylak et al., 2019; Rouphael and Colla, 2020). The broad modes of action of biostimulants range from triggering N metabolism or P release from soil to stimulating root growth and improving plant establishment (Madende and Hayes, 2020), which are advantageous activities from both an economic and environmental perspective (Rouphael and Colla, 2020). Furthermore, these products have not shown negative or harmful impacts on people and animals. The positive effects resulting from their use are influenced by many factors, including the type of bio-product, the dose, the application method, and the plant cultivar (Drobek et al., 2019).

The types of raw materials, solvents, and extraction methods have a significant influence on the final composition of biostimulants of plant growth (Pylak et al., 2019; Madende and Hayes, 2020). For biostimulant production, fresh (when unstable compounds are present) or dry materials, grounded into small particles, can be used. Water is the most popular solvent, but the use of organic ones (e.g., ethanol, methanol) allows to extract more substances (aromatic or saturated organic compounds), exhibiting better activity (e.g., antimicrobial) (Pylak et al., 2019). Biostimulants consist of various bioactive compounds, such as amino acids, peptides, proteins, nucleotides, nucleosides, lipids, phenols, phenolic acids, quinones, flavones, flavonoids, flavonols, tannins and coumarins, terpenoids/isoprenoids, alkaloids, glucosinolates, plant hormones (abscisic acid, auxins, cytokinins, gibberellins, ethylene), plant growth regulators (brassinosteroids), betaines, sugars (carbohydrates, oligo-, and polysaccharides), aminopolysaccharides, vitamins, humic substances, beneficial elements, furostanol glycosides, and sterols (Baghel et al., 2019; Pylak et al., 2019; Madende and Hayes, 2020). Because these chemical substances exhibit diverse effects, the modes of their action remain mostly unidentified (Paradiković et al., 2018; Bulgari et al., 2019; Caradonia et al., 2019; Drobek et al., 2019; Kim et al., 2019; Madende and Hayes, 2020). Furthermore, the composition of various biostimulants has still not been fully characterized (Pylak et al., 2019; Madende and Hayes, 2020), and biostimulants usually consist of complex multicomponents whose activities depend on the synergistic action of different bioactive molecules rather than individual compounds (Bulgari et al., 2019; Caradonia et al., 2019). Transcriptome analyses will allow us to scrutinize gene expression and thus provide greater knowledge of the biostimulant targets in plants, the affected physiological pathways, and the activated receptors. This information will improve our understanding of the effects and functions of their components (identified and unidentified) and may be used in the classification of new products and assessment of their effectiveness (Bulgari et al., 2015).

The current state of knowledge shows that different mechanisms may participate in the stimulating properties of biostimulants. Their positive effects on the yield and the quality of crops may result from the use of a single bio-product, as well as a combination of several products, allowing them to provide even better results. It is still necessary to undertake detailed wide-scale research on the impact of specific biostimulants on various species, treatments (e.g., doses and application methods), growth stages, etc. (Paradiković et al., 2018). We have conducted a series of experiments on various plant species, including radish (present study, field conditions), celeriac (field conditions) (Godlewska et al., 2020b), cabbage seedlings (laboratory tests) (Godlewska et al., 2019, 2020a), and white head cabbage (field conditions) (Godlewska et al., 2021), using botanical extracts (produced from St. John's wort, giant goldenrod, common dandelion, red clover, nettle, and valerian). Significant differences were observed in the plant responses to these bioproducts. The plant-based extracts that exerted the highest (↑) and lowest (↓) biostimulating activity of the examined parameters are summarized in Tables 10A,B. This comparison indicates the importance of precise planning of research, as well as studying the impact of the obtained products on various plant species. In general, extracts based on the common dandelion, valerian, nettle, and giant goldenrod could be considered in the cultivation of cabbage; those based on St. John's wort, common dandelion, and giant goldenrod could be used to cultivate celeriac; and those based on the common dandelion, valerian, giant goldenrod could be used for growing radish.

**Table 10A:** Effect of the foliar application of the examined botanical extracts on the growth and development of different model plants under controlled conditions (laboratory tests) and real environmental conditions (field trials).

**Examined parameter**		**Laboratory tests – cabbage seedlings** **(Godlewska et al., 2019, 2020a)**	**Field tests – cabbage** **(Godlewska et al., 2021)**	**Field tests – celeriac** **(Godlewska et al., 2020b)**	**Field tests – radish** **(current study)**
Fresh weight	↑	Shoots –Ur L UAE (98.8 and 106.1% more than in C and CB)– To F UAE (91.8 and 98.8% more than in C and CB) Roots –Vo R UAE (215.4 and 115.8% more than in C and CB) – Sg L UAE (184.6 and 94.7% more than in C and CB) – To F UAE (161.5 and 78.9% more than in C and CB) – To L UAE (176.9 and 89.5% more than in C and CB) – Hp H UAE (153.8 and 73.7% more than in C and CB) – Ur L UAE (146.2 and 68.4% more than in C and CB)	Head weight < 1.2 kg –Tp F UAE (50 and 106% more than in C and CB) – Vo R UAE (37.9 and 89.6% more than in C and CB) – Hp H MH (36.4 and 87.5% more than in C and CB) – To L UAE (36.4 and 87.5% more than in C and CB) – Ur L UAE (33.3 and 83.3% more than in C and CB) Outer leaves of head weight < 1.2 kg –Vo R UAE (37.5 and 49.4% more than in C and CB) – Ur L UAE (35.2 and 46.9% more than in C and CB) – Hp H MH (23.9 and 34.6% more than in C and CB) Head weight > 1.2 kg –Hp H UAE (8.8 and 3.6% more than in C and CB) – Ur L MH (2.3 more than in C and 2.6% less than in CB) – To F MH (1.5 more than in C and 3.3% less than in CB) Outer leaves of head weight > 1.2 kg –Sg L UAE (17.9 and 11.3% more than in C and CB) – Tp F MH (10.6 and 4.4% more than in C and CB) – Sg L MH (9.3 and 3.1% more than in C and CB) – Hp H UAE (7.9 and 1.9% more than in C and CB)	Root diameter < 5 cm Leaves of rosette –To F UAE (152.4 and 104.9% more than in C and CB) Roots –Tp F UAE (29.9 and 86.6% more than in C and CB) Root diameter 5–9 cm Leaves of rosette –To L MH (12.2% less than in C and 11.8% more than in CB) Roots –To L MH (5.3 less than in C and 11.9% more than in CB) Root diameter 9–13 cm Leaves of rosette –Hp H MH (no plants in C and 104.7% more than in CB) Roots – Hp H MH (no plants in C and 77.1% more than in CB)	Root diameter < 2 cm Leaves of rosette –To F UAE (2.4% less than in C and 20.4% more than in CB) – Vo R MH (3.9% less than in C and 18.5% more than in CB) Roots –Vo R MH (4.8% less than in C and 67.0% more than in CB) – Sg L MH (8.8% less than in C and 60.0% more than in CB) Root diameter 2–4 cm Leaves of rosette –Hp H MH (35.0 and 14.9% more than in C and CB) – Sg L MH (34.6 and 14.5% more than in C and CB) – Vo R MH (31.4 and 11.8% more than in C and CB) Roots –To L MH (50.7 and 24.4% more than in C and CB) – Sg L MH (42.7 and 17.9% more than in C and CB) Root diameter > 4 cm• Leaves of rosette –Hp H MH (no plants in C; 32.8% more than in CB) – Sg L UAE (no plants in C; 34.0% more than in CB) Roots –Tp F UAE (no plants in C; 10.2% more than in CB)
	↓	Shoots –Hp H UAE (0 and 3.7% more than in C and CB) Roots – Tp F UAE (84.6 and 26.3% more than in C and CB)	Head weight < 1.2 kg –Vo R MH (33.3 and 8.3% less than in C and CB) Outer leaves of head < 1.2 kg –To L UAE (26.1 and 19.8% less than in C and CB) –To F MH (23.9 and 17.3% less than in C and CB) Head weight > 1.2 kg –Sg L MH (14.6 and 18.6% less than in C and CB) –To F UAE (11.5 and 15.7% less than in C and CB) –Ur L UAE (10.0 and 14.2% less than in C and CB) Outer leaves of head weight > 1.2 kg –Ur L UAE (13.2 and 18.1% less than in C and CB) –To F UAE (8.6 and 13.8% less than in C and CB)	Root diameter < 5 cm Leaves of rosette –Sg L MH (2.8 and 21.2% less than in C and CB) Roots –Sg L MH (48.4 and 25.9% less than in C and CB) Root diameter 5–9 cm• Leaves of rosette –Tp F UAE (39.6 and 23.1% less than in C and CB) Roots –Vo R MH (31.4 and 18.9% less than in C and CB) Root diameter 9–13 cm• Leaves of rosette –To F MH (no plants in C; 3.0% less than in CB) Roots –Tp F MH (no plants in C; 7.5% less than in CB)	Root diameter < 2 cm Leaves of rosette –To L MH (30.4 and 14.1% less than in C and CB) – Ur L UAE (29.4 and 13.0% less than in C and CB) Roots –Ur L UAE (40.5% less than in C and 4.4% more than in CB) Root diameter 2–4 cm Leaves of rosette –To F UAE (4.3 and 18.5% less than in C and CB) Roots –Ur L UAE (15.2% more than in C and 4.9% less than in CB) – Ur L MH (16.6% more than in C and 3.7% less than in CB) Root diameter > 4 cm Leaves of rosette –Vo R UAE (no plants in C; 8.2% less than in CB) Roots –Hp H UAE (no plants in C; 35.7% less than in CB)
Dry weight	↑	Shoots –To F UAE (60.7 and 61.6% more than in C and CB) – Ur L UAE (60.2 and 61.0% more than in C and CB) Roots –Vo R UAE (53.3 and 33.3% more than in C and CB) – Sg L UAE (48.3 and 29.0% more than in C and CB) – To L UAE (48.3 and 29.0% more than in C and CB)	Leaves after the second spraying –To F MH (10.2 and 7.8% more than in C and CB) – Ur L UAE (7.5 and 5.2% more than in C and CB) Heads after harvest –Hp H UAE (3.7 and 7.1% more than in C and CB) – Vo R UAE (4.3 and 7.7% more than in C and CB)	The first term of leaves of rosette collection –Sg L UAE (0.4 and 1.3% more than in C and CB) The second term of leaves of rosette collection –Tp F MH (20.8 and 12.8% more than in C and CB) Roots –To F MH (28.7% more than in C and 2.2% less than in CB) – To L MH (28.7% more than in C and 2.2% less than in CB) – Tp F UAE (30.2% more than in C and 1.0% less than in CB) – Vo R MH (29.3% more than in C and 1.7% less than in CB)	The first term of leaves of rosette collection –Ur L UAE (7.5 and 17.2% more than in C and CB) The second term of leaves of rosette collection –Ur L UAE (8.3 and 10.9% more than in C and CB) Roots –Ur L UAE (37.9 and 51.8% more than in C and CB)
	↓	Shoots –Hp H UAE (1.3 and 1.8% more than in C and CB) Roots –Tp F UAE (27.0 and 10.4% more than in C and CB)	Leaves after the second spraying –Hp H UAE (7.6 and 9.6% less than in C and CB) Heads after harvest –Ur L MH (9.7 and 6.8% less than in C and CB)	The first term of leaves of rosette collection –Sg L MH (6.4 and 5.5% less than in C and CB) The second term of leaves of rosette collection –Vo R UAE (0.4% more than in C and 6.2% less than in CB) Roots –Ur L UAE (9.6% more than in C and 16.7% less than in CB)	The first trm of leaves of rosette collection –To L MH (12.7 and 4.8% less than in C and CB) The second term of leaves of rosette collection –To L MH (13.9 and 11.9% less than in C and CB) Roots –Sg L MH (7.3% less than in C and 2.1% more than in CB)
Chlorophyll *a* + *b*	↑	Leaves –To L UAE (109.6 and 1.0% more than in C and CB) – Hp H UAE (106.2% more than in C and 1.7% less than in CB) Roots n/a	Leaves after the second spraying –To L MH (46.0 and 24.0% more than in C and CB) – To F MH (31.5 and 11.6% more than in C and CB) – Hp H MH (30.6 and 11.0% more than in C and CB) – To L UAE (28.2 and 8.9% more than in C and CB) – Sg L UAE (25.8 and 6.8% more than in C and CB) – Sg L MH (25.0 and 6.2% more than in C and CB)	The first term of leaves of rosette collection –Ur L MH (17.6 and 2.2% more than in C and CB) – Sg L MH (16.7 and 1.2% more than in C and CB) – Hp H MH (16.7 and 1.2% more than in C and CB) The second term of leaves of rosette collection –To F UAE (11.4 and 34.7% more than in C and CB) – To F MH (5.9 and 28.1% more than in C and CB) Roots n/a	The first term of leaves of rosette collection –Vo R UAE (31.5 and 26.3% more than in C and CB) The second term of leaves of rosette collection –Vo R UAE (4.5 and 16.7% more than in C and CB) – Sg L MH (4.5 and 16.7% more than in C and CB) – Ur L MH (3.0 and 15.0% more than in C and CB) Roots n/a
	↓	Leaves –Vo R UAE (1.9% more than in C and 51.4% less than in CB) – To F UAE (7.7% more than in C and 48.6% less than in CB) Roots n/a	Leaves after the second spraying –To F UAE (6.5% more than in C and 9.6% less than in CB) – Vo R MH (9.7% more than in C and 6.8% less than in CB)	The first term of leaves of rosette collection –To L UAE (2.9 and 15.7% less than in C and CB) – Tp F UAE (0.5 and 13.6% less than in C and CB) The second term of leaves of rosette collection –Ur L UAE (9.3% less than in C and 9.7% more than in CB) – Vo R MH (8.9% less than in C and 10.2% more than in CB) Roots n/a	The first term of leaves of rosette collection –To L MH (16.4 and 19.7% less than in C and CB) The second term of leaves of rosette collection –To L UAE (19.4 and 10.0% less than in C and CB) – To F MH (19.4 and 10.0% less than in C and CB) – Sg L UAE (17.9 and 8.3% less than in C and CB) Roots n/a
SPAD	↑	Leaves –To L UAE (31.1 and 1.6% more than in C and CB) – Hp H UAE (31.7 and 2.1% more than in C and CB) Roots n/a	Leaves after the second spraying –To F MH (12.4 and 21.8% more than in C and CB) – Sg L MH (10.0 and 19.2% more than in C and CB) – Ur L MH (7.7 and 16.7% more than in C and CB) – Vo R MH (7.6 and 16.6% more than in C and CB) – To L UAE (7.4 and 16.4% more than in C and CB)	The first term of leaves of rosette collection –Hp H MH (10.7 and 10.9% more than in C and CB) The second term of leaves of rosette collection –To L MH (15.3 and 9.8% more than in C and CB) Roots n/a	The first term of leaves of rosette collection –Hp H MH (24.5 and 27.8% more than in C and CB) The second term of leaves of rosette collection –Vo R UAE (28.1 and 21.4% more than in C and CB) Roots n/a
	↓	Leaves –To F UAE (3.1% more than in C and 20.1% less than in CB) – Vo R UAE (6.2% more than in C and 17.6% less than in CB) Roots n/a	Leaves after the second spraying –Sg L UAE (0.8% less than in C and 7.6% more than in CB) – To F UAE (0.5 and 8.9% more than in C and CB)	The first term of leaves of rosette collection –Tp F MH (2.0 and 1.8% less than in C and CB) – Vo R UAE (0.7 and 0.5% less than in C and CB) The second term of leaves of rosette collection –Hp H MH (1.6 and 6.4% less than in C and CB) Roots n/a	The first term of leaves of rosette collection –Tp F MH (5.0 and 7.7% more than in C and CB) The second term of leaves of rosette collection –To L UAE (11.9 and 6.1% more than in C and CB) Roots n/a
Carotenoids	↑	Leaves –Hp H UAE (67.3% more than in C and 8.1% less than in CB) – Tp F UAE (67.2% more than in C and 8.1% less than in CB) – Vo R UAE (67.9% more than in C and 7.7% less than in CB) Roots n/a	Leaves after the second spraying –To L MH (21.8 and 19.2% more than in C and CB) – To L UAE (13.5 and 11.1% more than in C and CB)	The first term of leaves of rosette collection –To L MH (8.9 and 1.4% more than in C and CB) – Vo R UAE (9.4 and 1.9% more than in C and CB) The second term of leaves of rosette collection –To F UAE (7.1 and 30.8% more than in C and CB) Roots n/a	The first term of leaves of rosette collection –Vo R UAE (31.3 and 19.9% more than in C and CB) The second term of leaves of rosette collection –Vo R UAE (1.2 and 14.4% more than in C and CB) – Ur L MH (0.9 and 14.0% more than in C and CB) Roots n/a
	↓	Leaves –To F UAE (0.9% more than in C and 44.6% less than in CB) Roots n/a	Leaves after the second spraying –Vo R MH (4.4 and 6.4% less than in C and CB) – Ur L MH (2.5 and 4.5% less than in C and CB) – Tp F MH (2.4 and 4.5% less than in C and CB) – To F UAE (2.3 and 4.4% less than in C and CB)	The first term of leaves of rosette collection –Sg L UAE (14.0 and 19.9% less than in C and CB) The second term of leaves of rosette collection –Ur L UAE (14.2% less than in C and 4.7% more than in CB) Roots n/a	The first term of leaves of rosette collection –To L MH (28.7 and 34.9% less than in C and CB) The second term of leaves of rosette collection –and for To F MH (19.7 and 9.3% less than in C and CB) Roots n/a
Total phenolic compounds	↑	Shoots –Ur L UAE (23.3 and 7.5% more than in C and CB) Roots n/a	Leaves after the second spraying –Vo R UAE (39.7 and 136% more than in C and CB) – Sg L UAE (6.1 and 79.7% more than in C and CB) Heads after harvest –Vo R UAE (34.6 and 60.3% more than in C and CB) – Sg L UAE (6.9 and 27.4% more than in C and CB)	The first term of leaves of rosette collection –Hp H MH (30.8 and 50.2% more than in C and CB) The second term of leaves of rosette collection –Tp F MH (23.1% more than in C and 14.5% less than in CB) Roots –Hp H UAE (6.7 and 5.3 times more than in C and CB)	The first term of leaves of rosette collection –Sg L MH (1.1% less than in C but 23.8% more than in CB) – To F UAE (0.1 and 25.3% more than in C and CB) The second term of leaves of rosette collection –Sg L MH (10.7 and 2.9% more than in C and CB) – To F UAE (7.2% more than in C and 0.4% less than in CB) Roots –To F MH (13.2 and 50.5% more than in C and CB) – Sg L UAE (4.4 and 38.8% more than in C and CB)
	↓	Shoots –Vo R UAE (41.4 and 48.9% less than in C and CB) – Tp F UAE (32.8 and 41.4% less than in C and CB) Roots n/a	Leaves after the second spraying –Ur L MH (47.7 and 11.5% less than in C and CB) Heads after harvest –Ur L MH (29.7 and 16.3% less than in C and CB) – Hp H UAE (30.2 and 17.3% less than in C and CB)	The first term of leaves of rosette collection –Tp F UAE (43.7 and 35.4% less than in C and CB) The second term of leaves of rosette collection –Sg L UAE (28.6 and 50.4% less than in C and CB) Roots –Vo R UAE (14.0 and 31.1% less than in C and CB)	The first term of leaves of rosette collection –Tp F MH (22.7 and 3.3% less than in C and CB) – Sg L UAE (22.1 and 2.4% less than in C and CB) The second term of leaves of rosette collection –Hp H UAE (17.3 and 23.2% less than in C and CB) Roots –Tp F UAE (37.2 and 16.5% less than in C and CB) – To L MH (35.3 and 14.0% less than in C and CB) – Hp H UAE (34.7 and 13.2% less than in C and CB)
Antioxidant activity – DPPH	↑	Shoots –Ur L UAE (2.5 and 12.4% less than in C and CB) Roots n/a	Leaves after the second spraying –To L UAE (21.2 and 12.9% more than in C and CB) – Vo R UAE (16.8 and 8.8% more than in C and CB) – Sg L UAE (10.2 and 2.7% more than in C and CB) – Hp H UAE (5.1% more than in C and 2.0% less than in CB) – Vo R MH (2.9% more than in C and 4.1% less than in CB) Heads after harvest –Vo R MH (48.9 and 379% more than in C and CB) – Sg L UAE (40 and 350% more than in C and CB) – Hp H UAE (15.6 and 271% more than in C and CB)	The first term of leaves of rosette collection –Hp H MH (2.6 and 2.0 times more than in C and CB) – Sg L MH (2.6 and 2.0 times more than in C and CB) The second term of leaves of rosette collection –Sg L MH (21.3 and 62.2% more than in C and CB) Roots –Tp F UAE (5.2 and 3.2 times more than in C and CB)	The first term of leaves of rosette collection –To F UAE (76.5 and 147.1% more than in C and CB) – Vo R MH (79.8 and 151.8% more than in C and CB) The second term of leaves of rosette collection –Sg L UAE (28.6 and 51.8% more than in C and CB) Roots –To F MH (157.8 and 169.8% more than in C and CB)
	↓	Shoots –Sg L UAE (56.6 and 61.0% less than in C and CB) Roots n/a	Leaves after the second spraying –Tp F UAE (30.7 and 35.4% less than in C and CB) – Ur L UAE (31.4 and 36.1% less than in C and CB) – Tp F MH (33.6 and 38.1% less than in C and CB) Heads after harvest –Tp F UAE (37.8% less than in C and 100% more than in CB) – Tp F MH (51.1% less than in C and 57.1% more than in CB) – Vo R UAE (57.8% less than in C and 35.7% more than in CB)	The first term of leaves of rosette collection –Vo R UAE (31.8 and 47.6% less than in C and CB) The second term of leaves of rosette collection –Ur L MH (45.2 and 26.7% less than in C and CB) Roots –Ur L MH (27.8 and 55.2% less than in C and CB)	The first term of leaves of rosette collection –To L MH (35.3 and 9.4% less than in C and CB) – Tp F MH (33.6 and 7.1% less than in C and CB) The second term of leaves of rosette collection –To F MH (40.8 and 30.1% less than in C and CB) – Ur L MH (39.8 and 28.9% less than in C and CB) Roots –Sg L UAE (26.7 and 23.3% less than in C and CB) – To L UAE (33.3 and 30.2% less than in C and CB)
Antioxidant activity – ABTS	↑	Shoots –To F UAE (4.4 times and 40.3% more than in C and CB) – Ur L UAE (5.4 times and 73.9% more than in C and CB) Roots n/a	Leaves after the second spraying –Sg L MH (67.4 and 39.2% more than in C and CB) – Ur L UAE (56.6 and 30.2% more than in C and CB) – Hp H MH (48.5 and 23.5% more than in C and CB) – Hp H UAE (45.6 and 21.2% more than in C and CB) Heads after harvest –Hp H MH (47.7 and 121% more than in C and CB)	The first term of leaves of rosette collection –Vo R MH (73.7 and 44.9% more than in C and CB) – Hp H MH (50.8 and 25.8% more than in C and CB) The second term of leaves of rosette collection –Tp F UAE (10.7% less than in C and 7.7% more than in CB) Roots –To L UAE (127.9 and 132.3% more than in C and CB)	The first term of leaves of rosette collection –Vo R MH (25.0% less than in C and 104.4% more than in CB) The second term of leaves of rosette collection –Tp F UAE (21.8% less than in C and 227.9% more than in CB) – Vo R UAE (26.5% less than in C and 208.2% more than in CB) – Sg L UAE (27.8% less than in C and 202.6% more than in CB) Roots –To L UAE (177.7 and 10.7% more than in C and CB) – Sg L UAE (101.7 and 2.6% more than in C and CB) – Ur L UAE (100.9 and 2.2% more than in C and CB)
	↓	Shoots – Tp F UAE (138% more than in C and 23.6% less than in CB) – Vo R UAE (198% more than in C and 4.5% less than in CB) Roots n/a	Leaves after the second spraying –To F UAE (15.8% more than in C and 3.7% less than in CB) – Vo R UAE (18.7% more than in C and 1.3% less than in CB) Heads after harvest –To F UAE (72.1 and 58.2% less than in C and CB)	The first term of leaves of rosette collection –Vo R UAE (29.0 and 40.8% less than in C and CB) The second term of leaves of rosette collection –To L UAE (55.2 and 45.9% less than in C and CB) Roots –Sg L UAE (1.1 and 2.9% more than in C and CB)	The first term of leaves of rosette collection –To F MH (65.9 and 7.0% less than in C and CB) – Sg L UAE (65.7 and 6.5% less than in C and CB) The second term of leaves of rosette collection –Ur L UAE (73.4% less than in C and 11.5% more than in CB) Roots –Tp F MH (24.1% more than in C and 36.8% less than in CB) – To F UAE (25.0% more than in C and 36.4% less than in CB) – To L MH (28.4% more than in C and 34.6% less than in CB)
Antioxidant activity – FRAP	↑	Shoots –Ur L UAE (50.7 and 31.5% more than in C and CB) Roots n/a	Leaves after the second spraying –Hp H UAE (33.9 and 62.0% more than in C and CB) – Tp F UAE (28.3 and 55.3% more than in C and CB) – Sg L UAE (27.3 and 54.0% more than in C and CB) – Sg L MH (26.0 and 52.2% more than in C and CB) Heads after harvest –Tp F UAE (37.4 and 10.5% more than in C and CB) – Vo R MH (36.6 and 9.8% more than in C and CB)	The first term of leaves of rosette collection –Sg L MH (60.6 and 53.0% more than in C and CB) The second term of leaves of rosette collection –Sg L MH (0% more than in C and 13.3% less than in CB) Roots –To F MH (42.6 and 3.6% more than in C and CB)	The first term of leaves of rosette collection –Sg L MH (53.4 and 20.2% more than in C and CB) The second term of leaves of rosette collection –Sg L UAE (73.8 and 76.8% more than in C and CB) Roots – Sg L UAE (4.2 and 16.2% more than in C and CB) – Vo R MH (2.4% less than in C and 8.8% more than in CB)
	↓	Shoots –To L UAE (32.9 and 41.4% less than in C and CB) Roots n/a	Leaves after the second spraying –Ur L MH (16.1% less than in C and 1.5% more than in CB) – Hp H MH (14.5% less than in C and 3.5% more than in CB) – To L MH (13.8% less than in C and 4.3% more than in CB) Heads after harvest –To L MH (26.0 and 40.5% less than in C and CB)	The first term of leaves of rosette collection –To L MH (18.5 and 22.3% less than in C and CB) The second term of leaves of rosette collection –To L MH (29.3 and 38.7% less than in C and CB) Roots –Vo R UAE (3.0% more than in C and 25.2% less than in CB) – Tp F UAE (4.0% more than in C and 24.5% less than in CB) – Sg L UAE (5.9% more than in C and 23.0% less than in CB)	The first term of leaves of rosette collection –Hp H UAE (5.7 and 26.1% less than in C and CB) – Vo R UAE (6.1 and 26.4% less than in C and CB) The second term of leaves of rosette collection –Sg L MH (18.9 and 17.5% less than in C and CB) – To F UAE (16.6 and 15.2% less than in C and CB) Roots –Hp H MH (43.6 and 37.2% less than in C and CB)

*UAE, ultrasound-assisted extraction; MH, mechanical homogenisation; 1st term—samples of leaves of rosette taken for analyses 7 days after the second spraying; 2nd term—samples of leaves of rosette taken for analyses after harvesting; n/a, not analysed; C, control; CB, commercial biostimulant; Hp H, Hypericum perforatum L. (St. John's wort, herb); Sg L, Solidago gigantea Ait. (giant goldenrod, leaf); To F, To L, Taraxacum officinale (L.); Weber ex F. H. Wigg (common dandelion, flower, leaf); Tp F, Trifolium pratense L. (red clover, flower); Ur L, Urtica dioica L. (nettle, leaf); Vo R, Valeriana officinalis L. (valerian, root)*.

**Table 10B:** Effect of the foliar application of the examined botanical extracts on the growth and development of different model plants during real environment conditions (field trials).

**Examined parameter**			**Field tests** **– cabbage (Godlewska et al., 2021)**	**Field tests** **– celeriac (Godlewska et al., 2020b)**	**Field tests** **– radish (current study)**
Vitamin C	↑		Leaves after the second spraying –Tp F MH (52.1 and 25.9% more than in C and CB) – Sg L MH (31.0 and 8.4% more than in C and CB) – To F MH (29.2 and 6.9% more than in C and CB) – To F UAE (30.0 and 7.3% more than in C and CB) Heads after harvest –To L UAE (28.2 and 69.7% more than in C and CB)	The first term of leaves of rosette collection –Vo R UAE (57.6 and 41.6% more than in C and CB) The second term of leaves of rosette collection –Hp H MH (22.3 and 36.5% more than in C and CB) Roots –Ur L UAE (22.9 and 21.2% more than in C and CB)	The first term of leaves of rosette collection –Vo R MH (29.9 and 10.5% more than in C and CB) The second term of leaves of rosette collection –Vo R MH (115.5 and 44.4% more than in C and CB) Roots –Tp F MH (46.3 and 33.8% more than in C and CB) – Vo R UAE (42.2 and 30.2% more than in C and CB)
	↓		Leaves after the second spraying –Vo R UAE (0.8 and 17.9% less than in C and CB) – Sg L UAE (0.3 and 17.5% less than in C and CB) Heads after harvest –Ur L UAE (23.3% less than in C and 1.5% more than in CB) – To F MH (20.7% less than in C and 5.0% more than in CB)	The first term of leaves of rosette collection –To L MH (2.8 and 12.2% less than in C and CB) The second term of leaves of rosette collection –To F MH (24.7 and 15.8% less than in C and CB) Roots –Vo R UAE (13.9 and 15.1% less than in C and CB)	The first term of leaves of rosette collection –To L MH (6.9 and 20.7% less than in C and CB) The second term of leaves of rosette collection –To L MH (25.8% more than in C and 15.7% less than in CB) Roots –Vo R MH (10.5 and 18.2% less than in C and CB)
Nitrates	↑		Leaves after the second spraying –To L UAE (0.3% less than in C and 76.5% more than in CB) – Sg L UAE (16.7% less than in C and 47.4% more than in CB) – Ur L MH (19.5% less than in C and 42.5% more than in CB) – Tp F UAE (20.6% less than in C and 40.5% more than in CB) Heads after harvest –Tp F MH (186 and 115% more than in C and CB) – To F UAE (89.9 and 42.7% more than in C and CB) – Sg L MH (84.9 and 38.9% more than in C and CB) – To L MH (75.1 and 31.6% more than in C and CB) – To L UAE (73.0 and 30.0% more than in C and CB)	The first term of leaves of rosette collection –Ur L UAE (44.5 and 34.4% more than in C and CB) The second term of leaves of rosette collection –To L MH (140 and 52.8% more than in C and CB) Roots –Tp F UAE (15.8% less than in C and 22.2% more than in CB) – Ur L UAE (16.2% less than in C and 21.6% more than in CB)	The first term of leaves of rosette collection –Vo R MH (57.5 and 39.0% more than in C and CB) – To L MH (48.2 and 30.7% more than in C and CB) The second term of leaves of rosette collection –Ur L UAE (in leaves: 52.7 and 51.1% more than in C and CB) – Tp F UAE (51.9 and 50.4% more than in C and CB) Roots –Ur L UAE (73.7 and 124.7% more than in C and CB)
	↓		Leaves after the second spraying –Sg L MH (58.0 and 25.6% less than in C and CB) – To L MH (53.4 and 17.6% less than in C and CB) – Vo R MH (50.0 and 11.4% less than in C and CB) Heads after harvest –Vo R UAE (2.4% more than in C and 23.0% less than in CB) – Ur L MH (22.0% more than in C and 8.4% less than in CB) – Sg L UAE (26.4% more than in C and 5.1% less than in CB)	The first term of leaves of rosette collection –Tp F UAE (8.9 and 1.4% more than in C and CB) – Sg L MH (9.4 and 1.8% more than in C and CB) The second term of leaves of rosette collection –Vo R MH (27.5% more than in C and 18.8% less than in CB) Roots –Ur L MH (49.4 and 26.5% less than in C and CB) – Tp F MH (45.9 and 21.5% less than in C and CB)	The first term of leaves of rosette collection –Vo R UAE (21.9 and 31.1% less than in C and CB) – Ur L UAE (21.9 and 31.1% less than in C and CB) The second term of leaves of rosette collection –Vo R UAE (20.3 and 21.1% less than in C and CB, for both extracts) – To F UAE (20.3 and 21.1% less than in C and CB) Roots –Vo R UAE (20.2% less than in C and 3.2% more than in CB) – To F UAE (17.5% less than in C and 6.8% more than in CB) – Sg L MH (11.5% less than in C and 14.5% more than in CB)
Macroelements	↑	N	Heads after harvest –Sg L UAE (12.3 and 0.2% more than in C and CB) – To L MH (12.3 and 0.2% more than in C and CB)	Leaves of rosette –Vo R UAE (4.8 and 0.4% more than in C and CB) – Vo R MH (3.2% more than in C and 1.2% less than in CB) Roots –Hp H MH (4.8 and 1.8% more than in C and CB) – Vo R UAE (4.6 and 1.7% more than in C and CB) – Tp F MH (4.4 and 1.5% more than in C and CB)	Leaves of rosette –Ur L UAE (2.7% less than in C and 2.9% more than in CB) – Tp F UAE (3.4% less than in C and 2.1% more than in CB) Roots –Hp H UAE (5.7 and 4.2% more than in C and CB) – Vo R MH (4.6 and 3.0% more than in C and CB)
		P	Heads after harvest –Ur L MH (20.9% less than in C and 3.1% more than in CB) – Tp F MH (20.9% less than in C and 3.1% more than in CB)	Leaves of rosette –Ur L MH (6.9% less than in C and 26.8% more than in CB) Roots –Tp F MH (27.2 and 27.2% more than in C and CB) – Sg L UAE (23.1 and 23.1% more than in C and CB)	Leaves of rosette –To F MH (25.8 and 13.0% more than in C and CB) – Vo R MH (25.8 and 13.0% more than in C and CB) Roots –Ur L UAE (20.4 and 42.7% more than in C and CB) – Sg L UAE (19.7 and 41.9% more than in C and CB) – To F UAE (19.0 and 41.1% more than in C and CB)
		K	Heads after harvest –Sg L UAE (1.1% less than in C and 0.7% more than in CB)	Leaves of rosette –Vo R UAE (7.8% less than in C and 36.5% more than in CB) – Vo R MH (15.5% less than in C and 25.0% more than in CB) – Ur L MH (16.1% less than in C and 24.1% more than in CB) Roots –Sg L UAE (26.6 and 50.1% more than in C and CB) – Ur L UAE (20.6 and 43.0% more than in C and CB)	Leaves of rosette –Ur L UAE (29.7 and 8.8% more than in C and CB) – Vo R MH (27.2 and 6.7% more than in C and CB) Roots –Vo R MH (6.8 and 11.5% more than in C and CB) – To L UAE (4.8 and 9.4% more than in C and CB)
		Ca	Heads after harvest –To L UAE (36.1 and 27% higher than in C and CB) – Tp F MH (36.1 and 27% higher than in C and CB) – Sg L MH (28.6 and 20% higher than in C and CB) – To L MH (27.1 and 18.7% higher than in C and CB) – Vo R MH (26.6 and 18.2% higher than in C and CB)	Leaves of rosette –To F UAE (40.9 and 287% more than in C and CB) – Sg L MH (40.4 and 286% more than in C and CB) – Ur L MH (28.3 and 252% more than in C and CB) – Vo R MH (23.7 and 240% more than in C and CB) Roots –Ur L UAE (8.7 and 16.3% more than in C and CB)	Leaves of rosette –To L MH (2.5% less than in C and 3.4 more than in CB) – Sg L MH (3.2% less than in C and 2.7% more than in CB) Roots –Vo R MH (25.4 and 56.7% more than in C and CB) – Ur L MH (8.0 and 34.9% more than in C and CB) – Tp F MH (4.8 and 30.9% more than in C and CB)
		Mg	Heads after harvest –Tp F MH (17.9 and 9.8% higher than in C and CB) – Ur L UAE (13.7 and 5.9% higher than in C and CB) – To L MH (12.6 and 4.9% higher than in C and CB) – Sg L MH (12.6 and 4.9% higher than in C and CB) – To L UAE (11.6 and 3.9% higher than in C and CB)	Leaves of rosette –Vo R UAE (4.7 and 2.0% more than in C and CB) Roots –Sg L UAE (33.3 and 39.7% more than in C and CB) – Tp F UAE (31.3 and 37.6% more than in C and CB)	Leaves of rosette –Sg L UAE (3.1% more than in C and 1.5% less than in CB) – To L UAE (2.3% more than in C and 2.2% less than in CB) – Vo R MH (1.5% more than in C and 3.0% less than in CB) Roots –Vo R MH (75.1 and 96.3% more than in C and CB)
		S	Heads after harvest –Sg L MH (12.7 and 2.7% higher than in C and CB) – Hp H MH (10.9 and 1.0% higher than in C and CB) – To L MH (11.2 and 1.3% higher than in C and CB)	Leaves of rosette –To L MH (49.5 and 82.7% more than in C and CB) – Tp F MH (34.7 and 64.5% more than in C and CB) Roots –To L MH (36.4 and 76.0% more than in C and CB) – Hp H MH (36.4 and 76.0% more than in C and CB)	Leaves of rosette –To L MH (18.4 and 6.6% more than in C and CB) – Vo R UAE (13.2 and 2.0% more than in C and CB) Roots –Hp H UAE (0.4 and 14.2% more than in C and CB) – Sg L UAE (0.1% less than in C and 13.5% more than in CB) – To L UAE (0.3% less than in C and 13.4% more than in CB)
	↓	N	Heads after harvest –To L UAE (4.4 and 14.7% lower than in C and CB) – Sg L MH (3.4 and 13.7% lower than in C and CB) – Tp F MH (2.9 and 13.4% lower than in C and CB)	Leaves of rosette –To L MH (11.7 and 15.4% less than in C and CB) Roots –Ur L MH (7.1 and 9.7% less than in C and CB)	Leaves of rosette –Sg L UAE (8.1 and 2.9% less than in C and CB) – Vo R UAE (6.8 and 1.4% less than in C and CB) Roots –Sg L UAE (1.9 and 3.4% less than in C and CB) – Vo R UAE (0.0 and 1.5% less than in C and CB)
		P	Heads after harvest –Sg L MH (49.4 and 34.0% lower than in C and CB) – Vo R MH (45.5 and 28.9% lower than in C and CB)	Leaves of rosette –Hp H UAE (48.4 and 29.7% less than in C and CB) Roots –Vo R MH (1.8 and 1.8% less than in C and CB) – Vo R UAE (1.3 and 1.3% less than in C and CB)	Leaves of rosette –To L MH (34.0 and 40.7% less than in C and CB) – Hp H MH (3.1 and 13.0% less than in C and CB) Roots –Ur L MH (53.3 and 44.6% less than in C and CB) – Tp F MH (51.7 and 42.7% less than in C and CB)
		K	Heads after harvest –Sg L MH (14.6 and 13.1% lower than in C and CB) – Ur L MH (10.9 and 9.3% lower than in C and CB)	Leaves of rosette –To L MH (40.8 and 12.4% less than in C and CB) – Tp F MH (38.9 and 9.6% less than in C and CB) Roots –Hp H UAE (6.3% less than in C and 11.1% more than in CB) – To F MH (6.1% less than in C and 11.3% more than in CB)	Leaves of rosette –To F MH (4.6% more than in C and 12.2% less than in CB) Roots –Ur L MH (57.2 and 55.4% less than in C and CB) – Tp F MH (53.7 and 51.7% less than in C and CB)
		Ca	Heads after harvest –Ur L MH (4.8% higher than in C and 2.2% lower than in CB) – Vo R UAE (6.8% higher than in C and 0.3% lower than in CB)	Leaves of rosette –Ur L UAE (67.3 and 10.2% less than in C and CB) – Vo R UAE (65.9 and 6.4% less than in C and CB) Roots –To F UAE (3.9% less than in C and 2.8% more than in CB) – Vo R MH (2.9% less than in C and 3.8% more than in CB)	Leaves of rosette –Tp F UAE (19.0 and 14.0% less than in C and CB) Roots –Hp H UAE (21.6 and 2.1% less than in C and CB) – Sg L MH (21.2 and 1.6% less than in C and CB) – To L UAE (20.8 and 1.0% less than in C and CB)
		Mg	Heads after harvest –Hp H MH (1.1% higher than in C and 5.9% lower than in CB) – Vo R UAE (2.1% higher than in C and 4.9% lower than in CB) – Ur L MH (3.2% higher than in C and 3.9% lower than in CB) – To F UAE (4.2% higher than in C and 2.9% lower than in CB)	Leaves of rosette –Hp H UAE (17.7 and 19.8% less than in C and CB) Roots –To F MH (2.5% less than in C and 2.1% more than in CB) – Vo R MH (1.0% less than in C and 3.7% more than in CB) – To F UAE (0.5 and 5.3% more than in C and CB)	Leaves of rosette –Vo R UAE (9.7 and 13.7% less than in C and CB) Roots –Sg L MH (14.6 and 4.2% less than in C and CB) – Hp H UAE (13.6 and 3.2% less than in C and CB) – To L UAE (13.1 and 2.6% less than in C and CB)
		S	Heads after harvest –Vo R UAE (0.8% higher than in C and 8.2% lower than in CB) – Vo R MH (1.5% higher than in C and 7.5% lower than in CB) – Ur L MH (2.2% higher than in C and 6.9% lower than in CB)	Leaves of rosette –Vo R UAE (13.4% less than in C and 5.9% more than in CB) Roots –Tp F MH (10.9% less than in C and 15.0% more than in CB)	Leaves of rosette –To F UAE (1.8 and 11.5% less than in C and CB) – Vo R MH (1.4 and 11.2% less than in C and CB) Roots –Sg L MH (10.2% less than in C and 2.1% more than in CB)
Microelements and toxic elements	↑	Fe	Heads after harvest –Vo R MH (14.3 and 0.8% higher than in C and CB) – To L MH (12.2% higher than in C and 1.1% lower than in CB) – Sg L UAE (7.8% higher than in C and 5.0% lower than in CB)	Leaves of rosette –To F MH (2.4 times higher and 31.2% more than in C and CB) Roots –To L UAE (1.5 times and 70.7% more than in C and CB)	Leaves of rosette –To L UAE (24.3 and 32.3% more than in C and CB) – Hp H UAE (8.5%and 15.5% more than in C and CB) Roots –Vo R MH (27.9 and 43.3% more than in C and CB) – To L UAE (27.3 and 42.6% more than in C and CB) – Vo R UAE (26.9 and 42.2% more than in C and CB)
		Cu	Heads after harvest –To F UAE (1.5% higher than in C and 6.5% lower than in CB)	Leaves of rosette –Vo R UAE (9.8 and 9.5% more than in C and CB) Roots –To F UAE (3.2 and 30.5% more than in C and CB) – Hp H MH (0.3 and 26.7% more than in C and CB)	Leaves of rosette –To L UAE (4.4% more than in C and 5.3% less than in CB) – Vo R UAE (0.9% more than in C and 8.5% less than in CB) Roots –Vo R MH (45.5 and 58.9% more than in C and CB) – To F UAE (22.7 and 34.0% more than in C and CB)
		Zn	Heads after harvest –Sg L UAE (5.3% higher than in C and 3.6% lower than in CB)	Leaves of rosette –Vo R UAE (23.3 and 9.1% more than in C and CB) – Vo R MH (19.4 and 5.6% more than in C and CB) Roots –Sg L UAE (30.9 and 60.0% more than in C and CB)	Leaves of rosette –Sg L UAE (24.8 and 14.1% more than in C and CB) – Hp H UAE (17.2 and 7.1% more than in C and CB) Roots –To F UAE (3.2 and 7.5% more than in C and CB) – Vo R MH (2.1 and 6.4% more than in C and CB)
		Mn	Heads after harvest –Hp H MH (the same content as in C and CB)	Leaves of rosette –To L MH (61.4 and 30.3% more than in C and CB) – Tp F MH (54.8 and 25.0% more than in C and CB) Roots –Tp F UAE (33.5 and 34.4% more than in C and CB) – To L UAE (32.4 and 33.3% more than in C and CB)	Leaves of rosette –To L MH (3.8 and 6.5% more than in C and CB) – To L UAE (1.9 and 4.5% more than in C and CB) Roots –Hp H MH (30.7 and 9.8% more than in C and CB) – Vo R MH (29.3 and 8.6% more than in C and CB)
		Ni	Heads after harvest –To F MH (43.5% higher than in C and 16.9% lower than in CB) – To L MH (40.6% higher than in C and 18.6% lower than in CB)	Leaves of rosette –Sg L MH (3 times and 36.8% more than in C and CB) – Vo R MH (3 times and 36.8% more than in C and CB) Roots –Ur L MH (2 and 2.5 times more than in C and CB)	Leaves of rosette –Sg L UAE (6.4 and 26.8% more than in C and CB) – To F MH (0.4% less than in C and 18.7% more than in CB) – To F UAE (1.9% less than in C and 16.9% more than in CB) Roots –To F MH (107.1 and 96.1% more than in C and CB)
		Cd	Heads after harvest –Tp F UAE (77.8 and 77.8% higher than in C and CB) – Hp H UAE (66.7 and 66.7% higher than in C and CB) – Sg L MH (66.7 and 66.7% higher than in C and CB) – To F UAE (66.7 and 66.7% higher than in C and CB) – To L UAE (66.7 and 66.7% higher than in C and CB)	Leaves of rosette –To L MH (69.0 and 96.0% more than in C and CB) – Hp H UAE (58.6 and 84.0% more than in C and CB) Roots –Sg L UAE (40.7 and 123.5% more than in C and CB) – To L UAE (20.4 and 91.2% more than in C and CB) –Hp H UAE (18.5 and 88.2% more than in C and CB)	Leaves of rosette –Hp H MH (1.2 and 4.3% more than in C and CB) –Sg L MH (1.2 and 4.3% more than in C and CB) –Tp F MH (1.2 and 4.3% more than in C and CB) Roots –Vo R UAE (7.6 and 42.0% more than in C and CB) –To F UAE (3.0 and 36.0% more than in C and CB) –Tp F UAE (3.0 and 36.0% more than in C and CB)
		Pb	Heads after harvest –To L UAE (30.5 and 29.0% higher than in C and CB) –Hp H UAE (28.4 and 27.0% higher than in C and CB) –Sg L UAE (24.0 and 22.6% higher than in C and CB)	Leaves of rosette –Sg L UAE (26.2 and 131.1% more than in C and CB) – Vo R UAE (19.1 and 118.1% more than in C and CB) –To L UAE (17.3 and 114.7% more than in C and CB) Roots –To L UAE (13.9 and 190.3% more than in C and CB) – Vo R UAE (5.9 and 169.9% more than in C and CB)	Leaves of rosette –Hp H UAE (6.1 and 11.6% more than in C and CB) –To L UAE (3.7 and 9.1% more than in C and CB) Roots –Vo R UAE (2.0 and 55.1% more than in C and CB)
	↓	Fe	Heads after harvest –Tp F UAE (20.0 and 29.5% lower than in C and CB) –Tp F MH (18.5 and 28.1% lower than in C and CB)	Leaves of rosette –Vo R MH (15.2% more than in C and 36.5% less than in CB) Roots –Vo R MH (9.7% less than in C and 1.5% more than in CB)	Leaves of rosette –Sg L MH (26.4 and 21.6% less than in C and CB) – Ur L MH (23.1 and 18.1% less than in C and CB) –To L MH (22.7 and 17.7% less than in C and CB) Roots –Sg L MH (26.4 and 7.6% less than in C and CB)
		Cu	Heads after harvest –Tp F MH (19.1 and 25.5% lower than in C and CB) –Sg L MH (16.6 and 23.2% lower than in C and CB)	Leaves of rosette –Sg L MH (5.3 and 5.5% less than in C and CB) –Sg L UAE (4.7 and 5.0% less than in C and CB) Roots –Vo R UAE (21.6 and 1.0% less than in C and CB)	Leaves of rosette –Vo R MH (23.4 and 30.5% less than in C and CB) –Hp H UAE (21.5 and 28.8% less than in C and CB) Roots –Tp F UAE (10.4 and 2.1% less than in C and CB) –To L UAE (5.8% less than in C and 2.8% more than in CB)
		Zn	Heads after harvest –Tp F UAE (13.0 and 20.3% lower than in C and CB) –Hp H UAE (9.7 and 17.3% lower than in C and CB) –To L UAE (9.6 and 17.2% lower than in C and CB)	Leaves of rosette –Sg L MH (3.6 and 14.8% less than in C and CB) Roots –Sg L MH (12.6% less than in C and 6.9% more than in CB)	Leaves of rosette –Ur L MH (0.6% more than in C and 8.1% less than in CB) –To F MH (0.7% more than in C and 8.0% less than in CB) Roots –Sg L MH (11.8 and 8.1% less than in C and CB) –Tp F MH (9.9 and 6.1% less than in C and CB)
		Mn	Heads after harvest –Tp F MH (18.0 and 18.0% lower than in C and CB) –Hp H UAE (16.6 and 16.6% lower than in C and CB)	Leaves of rosette –Ur L MH (1.3 and 20.3% less than in C and CB) Roots –Hp H MH (9.2 and 8.5% less than in C and CB)	Leaves of rosette –Ur L MH (14.7 and 12.5% less than in C and CB) – Ur L UAE (12.5 and 10.2% less than in C and CB) Roots –Sg L UAE (13.2 and 27.0% less than in C and CB) –Hp H UAE (9.3 and 23.8% less than in C and CB)
		Ni	Heads after harvest –Vo R UAE (0.0% higher than in C and 42.1% lower than in CB) –Tp F UAE (4.2% higher than in C and 39.7% lower than in CB)	Leaves of rosette –Ur L UAE (15.9 and 62.2% less than in C and CB) Roots –Sg L MH (41.3 and 26.7% less than in C and CB)	Leaves of rosette –Tp F MH (23.1 and 8.3% less than in C and CB) – Vo R MH (22.5 and 7.6% less than in C and CB) Roots –Sg L MH (6.2 and 11.2% less than in C and CB) –Sg L UAE (5.6 and 10.7% less than in C and CB)
		Cd	Heads after harvest –To F MH (the same content as in C and CB) – Vo R UAE (the same content as in C and CB)	Leaves of rosette –Ur L MH (31.0 and 20.0% less than in C and CB) –To F UAE (3.4% less than in C and 12.0% more than in CB) Roots –Tp F MH (39.6% less than in C and 11.8% more than in CB) –To L MH (25.9% less than in C and 17.6% more than in CB) – Ur L MH (24.1% less than in C and 20.6% more than in CB)	Leaves of rosette –Sg L UAE (7.2 and 4.3% less than in C and CB) –To L UAE (7.2 and 4.3% less than in C and CB) –To F UAE (6.6 and 3.7% less than in C and CB) Roots –Sg L MH (19.7% less than in C and 6.0% more than in CB) – Ur L MH (18.2% less than in C and 8.0% more than in CB) –Tp F MH (16.7% less than in C and 10.0% more than in CB)
		Pb	Heads after harvest –Ur L MH (0.9 and 2.0% lower than in C and CB) –Hp H MH (2.3 and 1.2% higher than in C and CB)	Leaves of rosette –Tp F MH (60.8 and 28.2% less than in C and CB) – Ur L MH (59.9 and 26.6% less than in C and CB) Roots –Hp H MH (65.3 and 11.5% less than in C and CB) –To F MH (62.5 and 4.4% less than in C and CB)	Leaves of rosette –Ur L MH (14.3 and 9.8% less than in C and CB) Roots –To F MH (33.0% less than in C and 1.9% more than in CB) –Sg L MH (31.0% less than in C and 5.0% more than in CB)
Steam volatile compounds	↑		Heads after harvest e.g., 2-undecanone: –Sg L UAE (6.7 and 15.4% more than in C and CB) –To L UAE (6.0 and 14.6% more than in C and CB) e.g., trisulfide (dimethyl-): –To F UAE (11.6 and 11.1% more than in C and CB) – Vo R MH (10.5 and 10.0% more than in C and CB) –Tp F UAE (9.5 and 9.0% more than in C and CB) e.g., tetrasulfide (dimethyl-): –Hp H MH (28.3 and 16.0% more than in C and CB)	Leaves of rosette• e.g., β-myrcene: –Sg L MH (57.7 and 31.0% more than in C and CB) e.g., limonene: –Ur L UAE (15.3 and 11.8% more than in C and CB)	Leaves of rosette• e.g., β -myrcene: –Vo R UAE (7.4 and 25.9% more than in C and CB) –To L UAE (2.3 and 19.9% more than in C and CB) –Hp H UAE (1.1 and 18.5% more than in C and CB) e.g., limonene: –Vo R UAE (11.3 and 9.5% more than in C and CB) –To L UAE (4.8 and 3.1% more than in C and CB) –Hp H UAE (3.2 and 1.5% more than in C and CB)
	↓		Heads after harvest• e.g., 2-undecanone: –To F UAE (25.1 and 19.0% less than in C and CB) –Hp H MH (24.5 and 18.4% less than in C and CB) e.g., trisulfide (dimethyl-): –Sg L UAE (15.1 and 15.5% less than in C and CB) –Tp F MH (13.7 and 14.1% less than in C and CB) –To L UAE (12.0 and 12.4% less than in C and CB) e.g., tetrasulfide (dimethyl-): —-Tp F MH (24.9 and 32.1% less than in C and CB)	Leaves of rosette• e.g., β -myrcene: –Ur L UAE (8.2 and 23.8% less than in C and CB) e.g., limonene: –Tp F UAE (18.1 and 20.5% less than in C and CB)	Leaves of rosette• e.g., β -myrcene: –Tp F MH (33.4 and 21.9% less than in C and CB) e.g., limonene: –Tp F MH (25.4 and 26.6% less than in C and CB)
Fattyacids	↑		Heads after harvest• e.g., hexadecanoic acid: –To L MH (34.7 and 11.3% more than in C and CB) –To F MH (27.9 and 5.7% more than in C and CB) –Tp F MH (22.7 and 1.3% more than in C and CB) e.g., linolenic acid: –Vo R UAE (66.0 and 90.2% more than in C and CB) –To L UAE (55.3 and 78.0% more than in C and CB) –Sg L UAE (39.2 and 59.5% more than in C and CB) e.g., linoleic acid: –Vo R UAE (14.1 and 22.3% more than in C and CB) –Sg L UAE (13.5 and 21.7% more than in C and CB) – Ur L UAE (10.9 and 18.8% more than in C and CB) e.g., 9Z-9-octadecenoic acid (ethyl ester): –To L MH (43.6 and 50.2% more than in C and CB) – Vo R MH (39.5 and 46.0% more than in C and CB) e.g., octadecanoic acid: –To F MH (14.0 and 54.8% more than in C and CB) –Hp H UAE (7.1 and 45.4% more than in C and CB)	Roots• e.g., 9,12-hexadecadienoic acid (methyl ester): –Hp H MH (21.6% more than in C and 2.0% less than in CB) –Tp F MH (21.6% more than in C and 2.0% less than in CB) e.g., hexadecanoic acid, methyl ester: –To F UAE (9.4 and 0.2% less than in C and CB)	Roots• e.g., linolenic acid (methyl ester): –Ur L UAE (14.7 and 19.3% more than in C and CB) –Sg L MH (14.3 and 18.8% more than in C and CB) e.g., hexadecanoic acid (methyl ester): –Sg L MH (13.2% less than in C and 9.0% more than in CB) e.g., 9,12-hexadecadienoic acid (methyl ester): –Sg L MH (6.4 and 23.7% more than in C and CB)
	↓		Heads after harvest• e.g., hexadecanoic acid: –Vo R UAE (21.4 and 35.1% less than in C and CB) –Sg L UAE (12.9 and 28.0% less than in C and CB) e.g., linolenic acid: –To L MH (49.8 and 42.5% less than in C and CB) – Vo R MH (31.3 and 21.3% less than in C and CB) e.g., linoleic acid: –To L MH (32.3 and 27.4% less than in C and CB) – Vo R MH (22.7 and 17.2% less than in C and CB) e.g., 9Z-9-octadecenoic acid (ethyl ester): –Vo R UAE (40.8 and 38.0% less than in C and CB) –To L UAE (31.0 and 27.8% less than in C and CB) e.g., octadecanoic acid: –Vo R UAE (65.0 and 52.5% less than in C and CB) –To L UAE (60.2 and 45.9% less than in C and CB)	Roots• e.g., 9,12-hexadecadienoic acid (methyl ester): –To L MH (13.0% more than in C and 8.9% less than in CB) e.g., hexadecanoic acid, methyl ester: –Vo R UAE (35.7 and 29.2% less than in C and CB)	Roots• e.g., linolenic acid (methyl ester): –Ur L MH (27.2 and 24.3% less than in C and CB) –Tp F MH (24.8 and 21.8% less than in C and CB) e.g., hexadecanoic acid (methyl ester): –To F MH (34.7 and 18.0% less than in C and CB) e.g., 9,12-hexadecadienoic acid (methyl ester): –Ur L MH (38.7 and 28.7% less than in C and CB)
Sterols	↑		Heads after harvest• campesterol: –Vo R MH (10.1 and 11.1% more than in C and CB) –To F UAE (9.9 and 10.9% more than in C and CB) β-sitosterol: –Tp F MH (0.3 and 0.5% less than in C and CB) –Sg L UAE (0.3 and 0.5% less than in C and CB) – Hp H UAE (0.4 and 0.6% less than in C and CB)	n/a	Roots• campesterol (TMS derivative): –To L MH (15.7 and 14.5% more than in C and CB) β-sitosterol (TMS derivative): –Tp F UAE (1.9 and 2.5% more than in C and CB) –Sg L UAE (0.1 and 0.7% more than in C and CB).
	↓		Heads after harvest• campesterol: –Tp F MH (1.2 and 2.2% more than in C and CB) –Sg L UAE (1.4 and 2.3% more than in C and CB) β-sitosterol: –Vo R MH (2.3 and 2.5% less than in C and CB) –To F UAE (2.3 and 2.5% less than in C and CB)	n/a	Roots• campesterol (TMS derivative): –Tp F UAE (3.3 and 4.3% less than in C and CB) –Sg L UAE (0.2 and 1.3% less than in C and CB) β-sitosterol (TMS derivative): –To L MH (8.9 and 8.4% less than in C and CB)

*UAE, ultrasound-assisted extraction; MH, mechanical homogenisation; na, not analysed; C, control; CB, commercial biostimulant; Hp H, *Hypericum perforatum* L. (St. John's wort, herb); Sg L, *Solidago gigantea* Ait. (giant goldenrod, leaf); To F, To L, *Taraxacum officinale* (L.) Weber ex F. H. Wigg (common dandelion, flower, leaf); Tp F, *Trifolium pratense* L. (red clover, flower); Ur L, *Urtica dioica* L. (nettle, leaf); Vo R, *Valeriana officinalis* L. (valerian, root)*.

The application of plant-derived biostimulants has continued to rise by virtue of their favorable impact on the growth, yield, vigor, quality, and shelf life of some horticultural plants (e.g., due to the content of bioactive peptides that activate signaling pathways involving phytohormone biosynthesis) (Baghel et al., 2019; Caradonia et al., 2019; Jindo et al., 2020; Rouphael and Colla, 2020; Zulfiqar et al., 2020). Peptides are also engaged in the cell differentiation, induction of protease inhibitors, cell division, and pollen self-incompatibility responses (Kim et al., 2019). A higher content of proteins may be associated with an increase in the carbohydrate content in leaves. In turn, a greater amount of sugars generally accelerates nitrogen inclusions through the nitrate assimilation pathway. This increased growth can be also ascribed to sugars that serve as an energy source and stimulate nitrogen uptake. Carbohydrates provide the skeleton for the incorporation of reduced nitrate (ammonia) in amino acids and increased protein biosynthesis. The enhancement of sugar biosynthesis in plants is also linked to the higher content of chlorophyll, net photosynthesis, and quantum efficiency of photosystem II (Bulgari et al., 2015). Polysaccharides and a combination of diverse amino acids and short peptides (protein hydrolysates) exhibit a beneficial impact on plant growth and protection against abiotic and/or biotic stresses (Bulgari et al., 2019).

Biostimulants act in numerous stages of plant growth, from increasing the accessibility of nutrients in the soil to improving the postharvest quality of crops (even during stress conditions) (Zulfiqar et al., 2020). Their mechanisms, founded upon enhanced physiological, biochemical, and molecular processes, can be attributed to (a) the stimulation of essential enzymatic activities that correlate with the metabolism of nitrogen and the elicitation of target hormone-like activity (auxin and gibberellin; direct mechanism) and (b) the increase in the nutritional status of crops through the alteration of root development (biomass, density, and lateral root branching), which increases the absorption and translocation of macro and micro-nutrients (Caruso et al., 2019; Pylak et al., 2019; Zulfiqar et al., 2020). Higher nutrient availability can be affected by several factors, such as the presence of compounds in biostimulants (e.g., nutrients, amino acids, peptides, peptones, or proteins), improved microbial activity, or the content of chelating agents, thereby increasing nutrient solubility in soil. Biostimulants can also enhance nutrient uptake by upregulating the genes involved in their transport (Pylak et al., 2019), ameliorate the microbial and enzymatic activity of the soil, modify the solubility and transportability of micronutrients, increase soil cation exchange via nitrogen provision, and create more available complexes with insoluble elements (e.g., Fe) (Bulgari et al., 2019; Madende and Hayes, 2020). An improved photosynthesis process and carbon metabolism may also be observed (Zulfiqar et al., 2020). Studies showed that improved root growth is not necessarily associated with the presence of auxins due to their low concentrations but, instead, with organic compounds (e.g., amino acids, linear carboxylic acids, and aromatic carboxylic acids) that show auxin-like activity (Pylak et al., 2019).

The use of biostimulants can be particularly advantageous in poor soil conditions and in horticultural crops with low inputs (Bulgari et al., 2019). The inhibition of pathogen growth, antimicrobial activity, and defense mechanisms can be triggered by the presence of compounds that have phenolic structures, e.g., carvacrol, eugenol, or thymol (Pylak et al., 2019). Various antioxidants and elicitors, due to their synthesis in plants, can help deal with adverse growth conditions (e.g., temperature extremes, drought, and reduced nutrient absorption) (Paradiković et al., 2018). Biostimulants can alter the activity of enzymes and influence the antioxidant properties of compounds, such as lycopene, ascorbic acid, and phenolic compounds (Drobek et al., 2019).

The present study showed that extracts based on higher plants had a significant impact on the growth and physiological parameters of radish. The foliar application of the obtained formulations increased the yield of leaves of rosette (e.g., Sg L MH, Hp H MH), as well as the roots (e.g., To L MH, Sg L MH), dry weight (e.g., Ur L UAE), the content of photosynthetic pigments (e.g., Vo R UAE) and vitamin C (e.g., Vo R MH, Tp F MH). In the majority of cases, the tested extracts did not enhance the amount of total phenolic compounds (excluding, e.g., To F MH). The antioxidant activity (measured using the DPPH and ABTS assays) was mainly decreased in the leaves and increased in the roots after the application of the botanical extracts. The opposite trend was observed in the antioxidant activity measured using the FRAP assay. We also observed macronutrient enrichment in the leaves of rosette (e.g., Ur L UAE, Vo R MH, To L MH) and the roots (e.g., Hp H UAE, Vo R MH, Sg L UAE, To L UAE), alongside enrichment of micronutrients in the leaves of rosette (e.g., To L UAE, Hp H UAE, Vo R UAE, Sg L UAE) and the roots (e.g., Vo R MH, To F UAE). In most cases, the obtained bio-products lowered the content of heavy metals in the radish leaves and roots. The foliar application of the extracts exerted a varied impact on the composition of steam volatile compounds, fatty acids, sterols, and glucosinolates. The application of the bio-products resulted in higher content of nitrates in all parts of the model plant (e.g., Vo R MH, Ur L UAE).

The tested plant-based extracts are products that can be used in agriculture as potential biostimulants to reduce the cost of food production and achieve better quality yields. The raw materials (plants commonly found in the natural environment) constitute a rich source of biologically active compounds that exhibit a beneficial effect on the growth and chemical composition of crop plants. The final effect of their action depends on numerous factors (e.g., the raw material type, the extraction method, the application method, the plant variety, and the growth conditions). It is still necessary to conduct further research to gain a better understanding of their mechanisms. To understand the multiple effects of biostimulants, the development of appropriately designed experiments is crucial. Considering their positive effects on plants and various novel research possibilities, these biostimulants are attracting increasing attention every year among scientists, producers, and possible consumers. Appropriately prepared, characterized, and tested bio-products could be an integral component of sustainable agriculture in the foreseeable future.

## Data Availability Statement

The original contributions presented in the study are included in the article/Supplementary Material, further inquiries can be directed to the corresponding author.

## Author Contributions

KG designed and conducted all the research, analyzed the obtained data, and wrote the paper. PP participated in the field experiments, reviewed, and edited the paper. IM analyzed the obtained data and reviewed and edited the paper. AB supervised the work and reviewed and edited the paper. AS participated in the analyses of essential oils and fatty acids. NP participated in the analyses of essential oils and fatty acids. UP conducted the analyses of elements. All authors have read and agreed to the published version of the manuscript.

## Conflict of Interest

The authors declare that the research was conducted in the absence of any commercial or financial relationships that could be construed as a potential conflict of interest.

## Supplementary Material

Supplementary Figure 1 The weather conditions during the field experiments.

Supplementary Table 1 The effect of the foliar application of the botanical extracts on the profile of steam volatile compounds (the amount of a single component calculated as a percentage (%) of the whole GC-MS chromatogram area) (*N* = 3, mean ± SD) of radish leaves of rosette (after harvest). Statistically significant differences (*p* < 0.05) between the control group (C) and the botanical extracts. (b) Statistically significant differences (*p* < 0.05) between the formulation (CF) and the botanical extracts. (c) Statistically significant differences (*p* < 0.05) between commercial biostimulant (CB) and the botanical extracts. RT, retention time; RI, retention indices; RI_lit, retention indices according to NIST (The NIST Mass Spectral Search Program for the NIST/EPA/NIH EI and NIST Tandem Spectral Library, 2017), FFNSC (Mondello, 2015), Adams (Adams, 2017); RI_exp, retention indices based on experiments; UAE, ultrasound-assisted extraction; MH, mechanical homogenization; Hp H, *Hypericum perforatum* L. (St. John's wort, herb); Sg L, *Solidago gigantea* Ait. (giant goldenrod, leaf); To F, To L, *Taraxacum officinale* (L.) Weber ex F.H. Wigg (common dandelion, flower, leaf); Tp F, *Trifolium pratense* L. (red clover, flower); Ur L, *Urtica dioica* L. (nettle, leaf); Vo R, *Valeriana officinalis* L. (valerian, root).

Supplementary Table 2 The effect of the foliar application of the botanical extracts on the fatty acids composition [the amount of a single component calculated as a percentage (%) of the whole GC-MS chromatogram area] (*N* = 3, mean ± SD) of radish roots (after harvest). Statistically significant differences (*p* < 0.05) between the control group (C) and the botanical extracts. (b) Statistically significant differences (*p* < 0.05) between the formulation (CF) and the botanical extracts. (c) Statistically significant differences (*p* < 0.05) between commercial biostimulant (CB) and the botanical extracts. Abbreviations: RT, retention time; RI, retention indices; RI_lit, retention indices according to FFNSC (Mondello, 2015); RI_exp, retention indices based on experiments; UAE, ultrasound-assisted extraction; MH, mechanical homogenization; Hp H, *Hypericum perforatum* L. (St. John's wort, herb); Sg L, *Solidago gigantea* Ait. (giant goldenrod, leaf); To F, To L, *Taraxacum officinale* (L.) Weber ex F.H. Wigg (common dandelion, flower, leaf); Tp F, *Trifolium pratense* L. (red clover, flower); Ur L, *Urtica dioica* L. (nettle, leaf); Vo R, *Valeriana officinalis* L. (valerian, root).
