# Supplementary material for: Systematic Investigation of the Effects of Seven Plant Extracts on the Physiological Parameters, Yield, and Nutritional Quality of Radish (Raphanus sativus var. sativus)
Source: Front Plant Sci. 2021 Jun 17;12:651152. doi: 10.3389/fpls.2021.651152 (PMC8248541; doi:10.3389/fpls.2021.651152)
Supplement: Supplementary Figure 1 — The weather conditions during the field experiments. [file Data_Sheet_1.ZIP › SUPPLEMENTARY TABLES.docx]

**Table S1.** Effect of the foliar application of the botanical extracts on the profile of steam volatile compounds (the amount of a single component calculated as a percentage (%) of the whole GC-MS chromatogram area) (N = 3, mean ± SD) of radish leaves of rosette (after harvest).

| **Group** | **Hexanal** | **Heptane, 2,4-dimethyl-** | **Hex-(2*E*)-enal** | **Hex-(3*Z*)-enol** | **1-Hexanol** | **1-Nonene** | **Methional** | **α-Pinene** | **Benzaldehyde** | **β-Pinene** |
| --- | --- | --- | --- | --- | --- | --- | --- | --- | --- | --- |
| RT, min | 4.635 | 5.22 | 6.19 | 6.31 | 6.785 | 7.95 | 8.175 | 9.42 | 10.675 | 11.525 |
| RI_exp | 800 | 824 | 856 | 860 | 873 | 900 | 906 | 936 | 962 | 977 |
| RI_NIST | 800 | 821 | 854 | 857 | 868 | 892 | 907 | 937 | 962 | 979 |
| RI_FFNSC | 801 | 823 | 850 | 853 | 867 | 906 | 907 | 933 | 960 | 978 |
| RI_Adams | 801 | 821 | 855 | 856 | 870 | 904 | 906 | 939 | 960 | 979 |
| C | 1.26±0.02 b,c | 0.23±0.01 b,c | 1.72±0.02 b | 0.36±0.01 b,c | 0.42±0.02 | 0.79±0.02 | 0.21±0.02 b,c | 0.65±0.02 b | 0.51±0.02 c | 2.84±0.04 b |
| CF | 0.95±0.03 a | 0.73±0.04 a,c | 2.09±0.07 a,c | 0.71±0.04 a | 0.52±0.03 | 0.74±0.02 | 0.32±0.03 a,c | 0.53±0.03 a,c | 0.41±0.01 c | 2.56±0.06 a |
| CB | 0.91±0.03 a | 1.22±0.03 a,b | 1.87±0.03 b | 0.72±0.02 a | 0.44±0.03 | 0.72±0.03 | 0.60±0.01 a,b | 0.70±0.01 b | 0.64±0.02 a,b | 2.70±0.05 |
| Hp H UAE | 0.93±0.05 a | 0.36±0.05 b,c | 1.57±0.05 b,c | 0.44±0.03 b,c | 0.36±0.03 b | 0.52±0.02 a,b,c | 0.32±0.02 a,c | 0.57±0.02 c | 0.54±0.02 b | 2.58±0.09 a |
| Hp H MH | 0.92±0.02 a | 1.60±0.07 a,b,c | 0.79±0.03 a,b,c | 0.50±0.03 a,b,c | 0.37±0.05 b | 0.52±0.02 a,b,c | 0.15±0.01 b,c | 0.68±0.03 b | 0.59±0.04 b | 2.25±0.07 a,b,c |
| Sg L UAE | 0.94±0.03 a | 0.29±0.02 b,c | 1.34±0.02 a,b,c | 0.51±0.02 a,b,c | 0.35±0.03 b | 0.53±0.01 a,b,c | 0.20±0.01 b,c | 0.57±0.05 c | 0.51±0.04 c | 2.35±0.04 a,c |
| Sg L MH | 0.83±0.04 a | 1.91±0.05 a,b,c | 1.21±0.06 a,b,c | 0.68±0.03 a | 0.42±0.01 | 0.57±0.05 a,b,c | 0.32±0.02 a,c | 0.65±0.03 b | 0.65±0.03 a,b | 2.36±0.05 a,c |
| To F UAE | 0.55±0.03 a,b,c | 1.80±0.03 a,b,c | 0.92±0.02 a,b,c | 0.77±0.04 a | 0.42±0.03 b | 0.47±0.01 a,b,c | 0.36±0.03 a,c | 0.82±0.01 a,b,c | 0.86±0.02 a,b,c | 2.30±0.02 a,b,c |
| To F MH | 1.34±0.04 b,c | 1.32±0.04 a,b | 3.04±0.06 a,b,c | 1.08±0.01 a,b,c | 0.53±0.03 a | 0.62±0.03 a,b | 0.53±0.01 a,b,c | 0.66±0.02 b | 0.51±0.02 c | 2.47±0.07 a |
| To L UAE | 1.15±0.02 b,c | 0.94±0.05 a,b,c | 1.62±0.03 b,c | 1.07±0.09 a,b,c | 0.51±0.01 | 0.42±0.03 a,b,c | 0.61±0.02 a,b | 0.90±0.04 a,b,c | 0.41±0.01 c | 2.96±0.08 b,c |
| To L MH | 0.94±0.02 a | 1.52±0.03 a,b,c | 1.41±0.06 a,b,c | 0.41±0.03 b,c | 0.32±0.03 a,b,c | 0.74±0.03 | 0.12±0.01 a,b,c | 0.75±0.02 b | 0.55±0.04 b | 2.50±0.05 a |
| Tp F UAE | 1.06±0.05 a,c | 0.22±0.02 b,c | 2.37±0.06 a,b,c | 0.45±0.03 b,c | 0.29±0.01 a,b,c | 0.76±0.04 | 0.21±0.01 b,c | 0.52±0.05 a,c | 0.46±0.03 c | 2.44±0.04 a,c |
| Tp F MH | 1.06±0.05 a,c | 0.63±0.03 a,c | 2.39±0.04 a,b,c | 0.90±0.04 a,b,c | 0.42±0.02 b | 0.66±0.02 a | 0.19±0.01 b,c | 0.43±0.01 a,c | 0.55±0.03 b | 1.91±0.05 a,b,c |
| Ur L UAE | 1.12±0.02 a,b,c | 1.04±0.06 a,b,c | 1.25±0.03 a,b,c | 0.58±0.05 a,c | 0.45±0.03 | 0.64±0.02 a | 0.21±0.02 b,c | 0.53±0.03 a,c | 0.72±0.06 a,b | 2.25±0.07 a,b,c |
| Ur L MH | 1.24±0.02 b,c | 0.83±0.01 a,c | 2.12±0.04 a,c | 1.31±0.03 a,b,c | 0.49±0.02 | 0.83±0.03 | 0.37±0.02 a,c | 0.51±0.01 a,c | 0.92±0.02 a,b,c | 2.40±0.10 a,c |
| Vo R UAE | 0.86±0.02 a | 0.30±0.02 b,c | 2.08±0.02 a,c | 0.45±0.03 b,c | 0.37±0.03 b | 0.65±0.03 a | 0.28±0.01 a,c | 0.59±0.02 c | 0.33±0.02 a,c | 2.63±0.07 |
| Vo R MH | 1.06±0.06 a,c | 1.76±0.11 a,b,c | 1.11±0.05 a,b,c | 0.70±0.03 a | 0.43±0.02 | 0.43±0.04 a,b,c | 0.21±0.01 b,c | 0.73±0.04 b | 1.11±0.04 a,b,c | 2.21±0.05 a,b,c |

Statistically significant differences (p < 0.05) between the control group (C) and the botanical extracts. (b) Statistically significant differences (p < 0.05) between the formulation (CF) and the botanical extracts. (c) Statistically significant differences (p < 0.05) between commercial biostimulant (CB) and the botanical extracts. Abbreviations: RT, retention time; RI, retention indices; RI_lit, retention indices according to NIST (The NIST Mass Spectral Search Program for the NIST/EPA/NIH EI and NIST Tandem Spectral Library, 2017), FFNSC (Mondello, 2015), Adams (Adams, 2017); RI_exp, retention indices based on experiments; UAE, ultrasound-assisted extraction; MH, mechanical homogenisation; Hp H, *Hypericum perforatum* L. (St. John's wort, herb); Sg L, *Solidago gigantea* Ait. (giant goldenrod, leaf); To F, To L, *Taraxacum officinale* (L.) Weber ex F.H. Wigg (common dandelion, flower, leaf); Tp F, *Trifolium pratense* L. (red clover, flower); Ur L, *Urtica dioica* L. (nettle, leaf); Vo R, *Valeriana officinalis* L. (valerian, root).

**Table S1 – continuation.** Effect of the foliar application of the botanical extracts on the profile of steam volatile compounds (the amount of a single component calculated as a percentage (%) of the whole GC-MS chromatogram area) (N = 3, mean ± SD) of radish leaves of rosette (after harvest).

| **Group** | **5-Hepten-2-one, 6-methyl-** | **Myrcene** | **3-Octanol** | **α-Phellandrene** | **(2*E*,4*E*)-2,4-Heptadienal** | **p-Cymene** | **Limonene** | ***cis*-β-Ocimene** | **Benzeneacetaldehyde** | ***trans*-β-Ocimene** |
| --- | --- | --- | --- | --- | --- | --- | --- | --- | --- | --- |
| RT, min | 12.23 | 12.445 | 12.75 | 12.99 | 13.5 | 14.245 | 14.49 | 15.19 | 15.365 | 15.805 |
| RI_exp | 989 | 992 | 997 | 1002 | 1011 | 1025 | 1030 | 1042 | 1045 | 1052 |
| RI_NIST | 986 | 991 | 994 | 1005 | 1012 | 1025 | 1030 | 1038 | 1045 | 1049 |
| RI_FFNSC | 986 | 991 | 993 | 1000 | 1013 | 1025 | 1030 | 1035 | 1045 | 1049 |
| RI_Adams | 985 | 990 | 995 | 1002 | 1007 | 1024 | 1029 | 1037 | 1044 | 1050 |
| C | 0.25±0.02 b | 11.91±0.13 b,c | 0.41±0.02 b,c | 0.21±0.00 b,c | 1.11±0.02 c | 1.91±0.03 b,c | 51.14±0.11 b | 4.27±0.04 | 1.26±0.04 b | 0.36±0.02 |
| CF | 0.33±0.02 a,c | 11.48±0.08 a,c | 0.25±0.01 a | 0.35±0.03 a | 1.15±0.03 | 2.20±0.03 a,c | 53.14±0.23 a | 4.32±0.03 c | 1.44±0.04 a | 0.35±0.03 |
| CB | 0.24±0.01 b | 10.16±0.08 a,b | 0.27±0.02 a | 0.31±0.02 a | 1.29±0.03 a | 2.49±0.02 a,b | 51.97±0.31 | 3.78±0.06 b | 1.32±0.02 | 0.30±0.01 |
| Hp H UAE | 0.21±0.02 b | 12.04±0.09 b,c | 0.34±0.02 a,b,c | 0.14±0.02 b,c | 0.86±0.02 a,b,c | 1.68±0.04 a,b,c | 52.76±0.55 | 4.58±0.22 c | 1.34±0.08 | 0.25±0.01 a,b |
| Hp H MH | 0.31±0.02 c | 11.13±0.17 a,c | 0.15±0.01 a,b,c | 0.29±0.02 a | 1.05±0.04 c | 2.20±0.05 a,c | 47.79±0.12 a,b,c | 3.36±0.10 a,b | 0.42±0.02 a,b,c | 0.36±0.02 |
| Sg L UAE | 0.20±0.02 b | 11.05±0.10 a,b,c | 0.34±0.02 a,b,c | 0.10±0.01 a,b,c | 1.07±0.09 c | 1.38±0.04 a,b,c | 45.59±0.95 a,b,c | 3.50±0.06 a,b | 1.09±0.04 a,b,c | 0.28±0.01 |
| Sg L MH | 0.34±0.02 a,c | 9.54±0.06 a,b,c | 0.20±0.01 a | 0.70±0.01 a,b,c | 1.10±0.03 c | 4.11±0.09 a,b,c | 44.67±0.30 a,b,c | 3.75±0.45 a,b | 0.26±0.03 a,b,c | 0.23±0.01 a,b |
| To F UAE | 0.18±0.01 a,b | 9.42±0.03 a,b,c | 0.42±0.02 b,c | 0.83±0.02 a,b,c | 1.23±0.03 | 4.73±0.04 a,b,c | 43.24±0.37 a,b,c | 3.43±0.07 a,b | 0.12±0.01 a,b,c | 0.32±0.02 |
| To F MH | 0.23±0.02 b | 9.30±0.05 a,b,c | 0.29±0.01 a | 0.33±0.02 a | 1.06±0.08 c | 1.66±0.05 a,b,c | 45.45±0.39 a,b,c | 3.28±0.03 a,b,c | 1.03±0.03 a,b,c | 0.36±0.03 |
| To L UAE | 0.07±0.01 a,b,c | 12.18±0.11 b,c | 0.25±0.02 a | 0.13±0.01 a,b,c | 0.99±0.09 c | 2.46±0.07 a,b | 53.57±0.30 a | 4.07±0.07 | 1.12±0.05 b,c | 0.26±0.03 a,b |
| To L MH | 0.18±0.01 a,b | 10.07±0.13 a,c | 0.21±0.03 a | 0.44±0.03 a,b,c | 1.11±0.09 | 2.75±0.09 a,b,c | 44.96±0.71 a,b,c | 3.59±0.04 a,b | 0.33±0.02 a,b,c | 0.27±0.02 a,b |
| Tp F UAE | 0.21±0.01 b | 11.77±0.09 c | 0.30±0.01 a | 0.12±0.01 a,b,c | 1.13±0.02 | 1.65±0.05 a,b,c | 49.87±0.28 b,c | 3.86±0.04 | 1.34±0.07 | 0.35±0.03 |
| Tp F MH | 0.23±0.02 b | 7.93±0.08 a,b,c | 0.23±0.02 a | 0.37±0.01 a | 0.83±0.02 a,b,c | 1.22±0.02 a,b,c | 38.17±0.40 a,b,c | 2.93±0.06 a,b,c | 1.17±0.08 b | 0.17±0.01 a,b,c |
| Ur L UAE | 0.33±0.03 a,c | 11.25±0.06 a,c | 0.34±0.02 a,b,c | 0.35±0.02 a | 0.94±0.03 b,c | 2.50±0.06 a,b | 51.12±0.47 b | 3.83±0.09 | 0.51±0.03 a,b,c | 0.34±0.02 |
| Ur L MH | 0.53±0.01 a,b,c | 9.59±0.12 a,b,c | 0.19±0.01 a,c | 0.48±0.03 a,b,c | 1.20±0.04 | 1.49±0.03 a,b,c | 45.79±0.40 a,b,c | 3.19±0.04 a,b,c | 1.08±0.06 a,b,c | 0.27±0.02 a,b |
| Vo R UAE | 0.16±0.02 a,b,c | 12.79±0.05 a,b,c | 0.31±0.02 a | 0.17±0.02 b,c | 0.77±0.03 a,b,c | 1.79±0.07 b,c | 56.91±0.33 a,b,c | 4.41±0.07 c | 0.90±0.02 a,b,c | 0.44±0.04 a,b,c |
| Vo R MH | 0.27±0.02 | 9.64±0.09 a,b,c | 0.54±0.02 a,b,c | 0.61±0.02 a,b,c | 1.24±0.01 | 2.69±0.04 a,b | 45.28±0.44 a,b,c | 3.27±0.06 a,b,c | 0.11±0.02 a,b,c | 0.23±0.02 a,b |

Table S1 – continuation. Effect of the foliar application of the botanical extracts on the profile of steam volatile compounds (the amount of a single component calculated as a percentage (%) of the whole GC-MS chromatogram area) (N = 3, mean ± SD) of radish leaves of rosette (after harvest).

| **Group** | **γ-Terpinene** | **1-Octanol** | **Nonanal** | **2-Nonen-4-one** | ***trans*-Limonene oxide** | **2-Phenylpropenal** | ***cis*-Carveol** | ***trans*-Carveol** | **Carvone** | **Piperitone** |
| --- | --- | --- | --- | --- | --- | --- | --- | --- | --- | --- |
| RT, min | 16.355 | 17.305 | 19.34 | 20.705 | 21.355 | 22,545 | 26.2 | 26.78 | 28.33 | 28.995 |
| RI_exp | 1061 | 1075 | 1105 | 1128 | 1138 | 1157 | 1209 | 1219 | 1243 | 1253 |
| RI_NIST | 1060 | 1071 | 1104 | 1124 | 1138 | 1150 | 1208 | 1217 | 1246 | 1253 |
| RI_FFNSC | 1060 | 1076 | 1107 | 1124 | 1138 | 1151 | 1214 | 1223 | 1245 | 1253 |
| RI_Adams | 1059 | 1068 | 1100 | 1122 | 1142 | 1150 | 1214 | 1216 | 1243 | 1252 |
| C | 0.83±0.01 | 0.43±0.02 b,c | 1.85±0.05 b,c | 0.22±0.01 | 0.33±0.02 b,c | 0.55±0.02 b,c | 0.43±0.01 b,c | 0.81±0.02 b,c | 0.17±0.01 c | 0.10±0.00 c |
| CF | 0.93±0.03 | 0.19±0.01 a | 2.07±0.04 a,c | 0.19±0.01 | 0.19±0.01 a | 0.23±0.02 a,c | 0.24±0.01 a | 0.63±0.04 a | 0.13±0.01 | 0.09±0.00 c |
| CB | 0.88±0.05 | 0.25±0.01 a | 2.57±0.06 a,b | 0.18±0.01 | 0.24±0.01 a | 0.10±0.01 a,b | 0.20±0.01 a | 0.60±0.01 a | 0.11±0.02 a | 0.05±0.01 a,b |
| Hp H UAE | 0.82±0.03 | 0.36±0.05 b,c | 1.84±0.07 b,c | 0.21±0.02 | 0.30±0.02 b | 0.52±0.02 b,c | 0.23±0.02 a | 0.60±0.04 a | 0.11±0.01 a | 0.11±0.01 c |
| Hp H MH | 0.60±0.01 a,b,c | 0.14±0.00 a,c | 1.66±0.05 a,b,c | 0.14±0.02 a | 0.50±0.01 a,b,c | 0.37±0.04 a,b,c | 0.36±0.04 b,c | 0.51±0.06 a | 0.25±0.03 a,b,c | 0.08±0.00 |
| Sg L UAE | 0.66±0.03 a,b,c | 0.10±0.01 a,b,c | 1.62±0.02 a,b,c | 0.22±0.02 | 0.24±0.02 a | 0.10±0.02 a,b | 0.26±0.03 a | 0.65±0.06 a | 0.08±0.01 a,b | 0.08±0.01 |
| Sg L MH | 0.83±0.03 | 0.31±0.02 a,b | 1.73±0.03 b,c | 0.07±0.01 a,b,c | 0.80±0.02 a,b,c | 0.16±0.01 a,b | 0.31±0.02 a,c | 0.58±0.03 a | 0.08±0.01 a,b | 0.03±0.01 a,b |
| To F UAE | 0.81±0.05 b | 0.62±0.02 a,b,c | 2.13±0.02 a,c | 0.26±0.02 b,c | 0.13±0.01 a,c | 0.16±0.01 a,b | 0.45±0.03 b,c | 0.69±0.05 | 0.02±0.00 a,b,c | 0.63±0.02 a,b,c |
| To F MH | 0.86±0.03 | 0.22±0.01 a | 2.19±0.04 a,c | 0.37±0.01 a,b,c | 0.22±0.03 a | 0.16±0.01 a,b | 0.39±0.03 b,c | 0.71±0.01 | 0.05±0.00 a,b,c | 0.28±0.01 a,b,c |
| To L UAE | 0.96±0.02 a | 0.36±0.02 b,c | 1.99±0.03 c | 0.13±0.01 a | 0.26±0.03 | 0.45±0.02 a,b,c | 0.21±0.02 a | 0.49±0.02 a,b | 0.09±0.01 a | 0.11±0.01 c |
| To L MH | 0.66±0.02 a,b,c | 0.13±0.01 a,c | 1.83±0.08 b,c | 0.12±0.00 a,b | 0.46±0.02 a,b,c | 0.35±0.02 a,b,c | 0.29±0.03 a,c | 0.43±0.03 a,b,c | 0.15±0.01 | 0.05±0.00 a,b |
| Tp F UAE | 0.79±0.02 b | 0.32±0.02 a,b,c | 1.76±0.08 b,c | 0.24±0.03 | 0.26±0.02 | 0.46±0.02 a,b,c | 0.31±0.01 a,c | 0.84±0.02 b,c | 0.10±0.01 a | 0.09±0.01 c |
| Tp F MH | 0.65±0.02 a,b,c | 0.20±0.01 a | 1.85±0.06 b,c | 0.10±0.01 a,b,c | 0.21±0.01 a | 0.22±0.02 a,c | 0.42±0.03 b,c | 0.46±0.03 a,b,c | 0.11±0.01 a | 0.19±0.03 a,b,c |
| Ur L UAE | 0.98±0.03 a | 0.43±0.02 b,c | 1.82±0.03 b,c | 0.13±0.02 a | 0.24±0.02 a | 0.16±0.01 a,b | 0.34±0.02 a,b,c | 0.63±0.03 a | 0.03±0.01 a,b,c | 0.08±0.01 |
| Ur L MH | 0.64±0.02 a,b,c | 0.36±0.03 a,b,c | 2.47±0.03 a,b | 0.22±0.03 | 0.42±0.02 a,b,c | 0.17±0.01 a,c | 0.43±0.03 b,c | 0.76±0.03 c | 0.08±0.01 a,b | 0.06±0.01 |
| Vo R UAE | 0.90±0.02 | 0.31±0.02 a,b | 1.69±0.05 b,c | 0.20±0.00 | 0.34±0.02 b,c | 0.39±0.02 a,b,c | 0.30±0.01 a,c | 0.61±0.05 a | 0.11±0.00 a | 0.07±0.00 |
| Vo R MH | 0.90±0.02 | 0.10±0.01 a,b,c | 1.79±0.04 b,c | 0.48±0.02 a,b,c | 0.24±0.03 a | 0.05±0.01 a,b | 0.34±0.04 a,b,c | 0.64±0.02 a | 0.10±0.01 a | 0.17±0.01 a,b,c |

Table S1 – continuation. Effect of the foliar application of the botanical extracts on the profile of steam volatile compounds (the amount of a single component calculated as a percentage (%) of the whole GC-MS chromatogram area) (N = 3, mean ± SD) of radish leaves of rosette (after harvest).

| **Group** | ***trans*-2-Decenal** | **Verdoracine** | ***cis*-Geranylacetone** | **Ionone epoxide** | **Pentadecanal** | **Hexahydrofarnesyl acetone** | **Farnesyl acetone** | **Phytol** |
| --- | --- | --- | --- | --- | --- | --- | --- | --- |
| RT, min | 29.67 | 35.545 | 41.865 | 43.74 | 54.99 | 57.02 | 57.835 | 59.47 |
| RI_exp | 1263 | 1353 | 1454 | 1484 | 1716 | 1848 | 1920 | 2114 |
| RI_NIST | 1263 | 1354 | 1449 | 1483 | 1715 | 1844 | 1919 | 2114 |
| RI_FFNSC | 1265 | 1357 | 1451 | 1483 | 1715 | 1844 | 1920 | 2113 |
| RI_Adams | 1263 | 1355 | 1450 | 1485 | 1715 | 1845 | 1913 | 2115 |
| C | 0.11±0.01 b,c | 0.81±0.01 b,c | 0.73±0.02 c | 2.35±0.03 | 0.19±0.01 b | 1.40±0.03 | 0.54±0.02 | 6.32±0.03 b,c |
| CF | 0.04±0.01 a,c | 1.10±0.03 a,c | 0.78±0.05 c | 2.20±0.07 c | 0.34±0.02 a,c | 1.26±0.03 c | 0.60±0.03 | 4.26±0.19 a |
| CB | 0.19±0.01 a,b | 1.67±0.03 a,b | 0.93±0.03 a,b | 2.41±0.11 b | 0.20±0.01 b | 1.49±0.05 b | 0.53±0.04 | 4.77±0.26 a |
| Hp H UAE | 0.09±0.01 b,c | 1.23±0.05 a,c | 0.71±0.02 c | 2.33±0.05 | 0.10±0.00 a,b,c | 1.71±0.05 a,b,c | 0.50±0.03 | 7.13±0.14 b,c |
| Hp H MH | 0.09±0.00 b,c | 1.34±0.04 a,b,c | 0.75±0.04 c | 2.16±0.02 c | 0.29±0.02 a,c | 1.60±0.02 a,b | 0.81±0.02 a,b,c | 13.00±0.14 a,b,c |
| Sg L UAE | 0.10±0.01 b,c | 1.04±0.06 a,c | 0.73±0.04 c | 1.96±0.04 a,b,c | 0.21±0.02 b | 1.80±0.07 a,b,c | 0.61±0.02 | 17.37±0.47 a,b,c |
| Sg L MH | 0.02±0.01 a,c | 1.65±0.02 a,b | 0.51±0.02 a,b,c | 1.98±0.03 a,b,c | 0.29±0.01 a,c | 1.74±0.09 a,b,c | 0.54±0.05 | 13.91±0.15 a,b,c |
| To F UAE | 0.17±0.01 a,b | 1.48±0.02 a,b,c | 0.83±0.05 | 2.46±0.07 b | 0.32±0.01 a,c | 1.59±0.02 a,b | 0.85±0.05 a,b,c | 13.25±0.20 a,b,c |
| To F MH | 0.13±0.00 b,c | 1.62±0.06 a,b | 0.69±0.02 c | 2.17±0.05 c | 0.17±0.02 b | 1.34±0.06 | 0.53±0.03 | 12.82±0.37 a,b,c |
| To L UAE | 0.15±0.01 b,c | 3.23±0.07 a,b,c | 0.71±0.03 c | 1.51±0.05 a,b,c | 0.10±0.01 a,b,c | 0.73±0.03 a,b,c | 0.32±0.02 a,b,c | 2.72±0.17 a,b,c |
| To L MH | 0.05±0.01 a,c | 1.70±0.05 a,b | 0.82±0.04 | 2.28±0.04 | 0.40±0.03 a,c | 1.81±0.07 a,b,c | 0.82±0.05 a,b,c | 14.65±0.43 a,b,c |
| Tp F UAE | 0.10±0.01 b,c | 1.08±0.05 a,c | 0.71±0.04 c | 2.54±0.05 b | 0.20±0.01 b | 2.40±0.05 a,b,c | 0.69±0.04 a,c | 7.71±0.23 a,b,c |
| Tp F MH | 0.03±0.01 a,c | 1.02±0.05 a,c | 0.64±0.03 b,c | 1.70±0.06 a,b,c | 0.45±0.05 a,b,c | 1.58±0.07 b | 0.65±0.03 | 27.12±0.20 a,b,c |
| Ur L UAE | 0.12±0.01 b,c | 0.87±0.02 b,c | 1.06±0.05 a,b,c | 2.37±0.05 | 0.38±0.02 a,c | 1.36±0.03 | 0.68±0.02 a,c | 8.05±0.17 a,b,c |
| Ur L MH | 0.10±0.00 b,c | 1.11±0.05 a,c | 0.96±0.03 a,b | 2.70±0.04 a,b,c | 0.39±0.01 a,c | 2.08±0.05 a,b,c | 0.82±0.03 a,b,c | 11.41±0.32 a,b,c |
| Vo R UAE | 0.10±0.01 b,c | 1.22±0.06 a,c | 0.72±0.02 c | 2.18±0.06 c | 0.14±0.02 b | 0.81±0.04 a,b,c | 0.49±0.02 | 2.25±0.07 a,b,c |
| Vo R MH | 0.08±0.01 b,c | 1.70±0.02 a,b | 1.02±0.04 a,b | 2.34±0.04 | 0.46±0.02 a,b,c | 2.12±0.04 a,b,c | 1.03±0.02 a,b,c | 12.84±0.20 a,b,c |

Table S2. Effect of the foliar application of the botanical extracts on the fatty acids composition (the amount of a single component calculated as a percentage (%) of the whole GC-MS chromatogram area) (N = 3, mean ± SD) of radish roots (after harvest).

| **Group** | **Dodecanoic acid, methyl ester** | **Tridecanoic acid, 12-methyl-, methyl ester** | **Tetradecanoic acid, methyl ester** | **Tetradecanoic acid, ethyl ester** | **Tetradecanoic acid, 12-methyl-, methyl ester,** | **Pentadecanoic acid, methyl ester** | ***Z*-6-Octadecenoic acid, methyl ester,** | **Pentadecanoic acid, 14-methyl-, methyl ester** | **Hexadecanoic acid, methyl ester** | ***Z*-9-Hexadecenoic acid, methyl ester,** | **Hexadecanoic acid, 15-methyl-, methyl ester** | **Hexadecanoic acid, 14-methyl-, methyl ester** |
| --- | --- | --- | --- | --- | --- | --- | --- | --- | --- | --- | --- | --- |
| RT, min | 18.780 | 22.980 | 24.265 | 25.660 | 26.100 | 26.890 | 27.390 | 28.240 | 29.515 | 30.440 | 30.715 | 31.135 |
| RI_exp | 1200 | 1352 | 1400 | 1463 | 1470 | 1500 | 1517 | 1552 | 1600 | 1635 | 1651 | 1687 |
| RI_lit | 1203 | 1353 | 1402 | 1471 | 1474 | 1503 | 1523 | 1556 | 1599 | 1632 | 1656 | 1693 |
| C | 0.2±0.01 b,c | 0.59±0.02 b,c | 0.88±0.02 b,c | 3.09±0.05 b,c | 3.32±0.07 b,c | 0.36±0.01 b,c | 0.32±0.02 b,c | 3.45±0.04 b,c | 22.97±0.20 b,c | 2.93±0.06 b,c | 1.97±0.00 b,c | 1.86±0.02 b,c |
| CF | 0.08±0.00 a | 0.35±0.01 a | 0.32±0.00 a,c | 1.26±0.02 a,c | 1.72±0.01 a,c | 0.24±0.00 a | 0.22±0.01 a | 1.60±0.03 a,c | 16.44±0.08 a,c | 1.59±0.01 a,c | 1.00±0.01 a,c | 0.79±0.01 a |
| CB | 0.09±0.00 a | 0.25±0.01 a | 0.44±0.01 a,b | 0.95±0.02 a,b | 1.30±0.03 a,b | 0.23±0.02 a | 0.22±0.00 a | 1.28±0.02 a,b | 18.11±0.15 a,b | 2.34±0.04 a,b | 0.84±0.05 a,b | 0.70±0.03 a |
| Hp H UAE | 0.63±0.02 a,b,c | 0.41±0.03 a,c | 0.50±0.03 a,b | 1.78±0.04 a,b,c | 2.24±0.03 a,b,c | 0.21±0.01 a | 0.24±0.04 | 2.16±0.03 a,b,c | 18.30±0.15 a,b | 1.76±0.03 a,c | 1.15±0.03 a,c | 1.20±0.02 a,b,c |
| Hp H MH | 1.89±0.03 a,b,c | 0.40±0.07 a | 0.31±0.02 a,c | 1.22±0.02 a,c | 1.36±0.03 a,b | 0.22±0.05 a | 0.35±0.05 b,c | 1.66±0.03 a,c | 17.21±0.33 a,b,c | 1.48±0.03 a,c | 1.11±0.08 a,c | 0.91±0.03 a,b,c |
| Sg L UAE | 0.35±0.04 a,b,c | 0.60±0.03 b,c | 0.46±0.01 a,b | 2.95±0.06 b,c | 3.37±0.05 b,c | 0.24±0.00 a | 0.22±0.03 a | 3.30±0.05 b,c | 16.89±0.12 a,c | 1.64±0.04 a,c | 1.75±0.04 a,b,c | 1.75±0.03 b,c |
| Sg L MH | 0.78± 0.07 a,b,c | 0.40 ±0.06 a | 0.46 ±0.04 a,b | 1.46 ±0.06 a,b,c | 1.75± 0.04 a,c | 0.26± 0.01 a | 0.29 ±0.05 | 1.88± 0.08 a,b,c | 19.95 ±0.17 a,b,c | 2.55 ±0.07 a,b,c | 1.14± 0.05 a,c | 0.99± 0.02 a,b,c |
| To F UAE | 0.30±0.01 b,c | 0.57±0.02 b,c | 0.46±0.02 a,b | 3.09±0.08 b,c | 3.60±0.05 a,b,c | 0.30±0.02 c | 0.19±0.02 a | 3.59±0.10 b,c | 18.94±0.08 a,b,c | 1.70±0.08 a,c | 2.01±0.04 b,c | 2.00±0.05 a,b,c |
| To F MH | 0.12±0.01 | 0.29±0.03 a | 0.38±0.02 a | 1.54±0.06 a,b,c | 1.92±0.07 a,b,c | 0.25±0.02 a | 0.19±0.01 a | 1.70±0.07 a,c | 15.02±0.26 a,b,c | 1.68±0.07 a,c | 1.18±0.05 a,b,c | 1.03±0.03 a,b,c |
| To L UAE | 0.55±0.03 a,b,c | 0.54±0.03 b,c | 0.45±0.01 a,b | 2.63±0.06 a,b,c | 3.15±0.05 b,c | 0.22±0.02 a | 0.20±0.02 a | 3.17±0.08 a,b,c | 17.18±0.08 a,b,c | 1.11±0.03 a,b,c | 1.59±0.02 a,b,c | 1.67±0.04 a,b,c |
| To L MH | 0.51±0.03 a,b,c | 0.33±0.06 a | 0.43±0.02 a,b | 1.37±0.05 a,c | 1.68±0.07 a,c | 0.24±0.01 a | 0.23±0.02 | 1.69±0.04 a,c | 18.99±0.21 a,b,c | 2.40±0.04 a,b | 1.15±0.05 a,c | 0.88±0.04 a,c |
| Tp F UAE | 0.27±0.03 b,c | 0.45±0.08 c | 0.58±0.02 a,b,c | 2.92±0.06 a,b,c | 3.33±0.07 b,c | 0.25±0.01 a | 0.10±0.02 a,b,c | 2.89±0.21 a,b,c | 17.04±0.10 a,c | 3.03±0.08 b,c | 1.84±0.04 b,c | 1.76±0.04 b,c |
| Tp F MH | 0.15±0.01 | 0.26±0.02 a | 0.60±0.01 a,b,c | 1.08±0.01 a,b | 1.56±0.03 a,c | 0.27±0.01 a | 0.15±0.02 a | 1.27±0.01 a,b | 15.31±0.10 a,b,c | 1.93±0.02 a,b,c | 0.89±0.02 a | 0.73±0.03 a |
| Ur L UAE | 0.34±0.01 a,b,c | 0.25±0.02 a | 0.63±0.02 a,b,c | 1.23±0.04 a,c | 1.59±0.04 a,c | 0.27±0.01 a | 0.12±0.02 a,b,c | 1.43±0.05 a | 18.76±0.08 a,b,c | 1.63±0.04 a,c | 0.81±0.03 a,b | 0.85±0.03 a,c |
| Ur L MH | 0.10±0.01 | 0.27±0.01 a | 0.39±0.00 a | 1.19±0.01 a,c | 1.74±0.03 a,c | 0.24±0.00 a | 0.15±0.00 a | 1.59±0.02 a,c | 15.35±0.16 a,b,c | 1.72±0.01 a,c | 0.97±0.02 a | 0.97±0.01 a,b,c |
| Vo R UAE | 1.36±0.02 a,b,c | 0.58±0.04 b,c | 0.72±0.02 a,b,c | 2.82±0.02 a,b,c | 2.86±0.09 a,b,c | 0.24±0.02 a | 0.22±0.02 a | 2.98±0.01 a,b,c | 17.57±0.15 a,b | 1.93±0.04 a,b,c | 1.73±0.07 a,b,c | 1.41±0.05 a,b,c |
| Vo R MH | 0.41±0.02 a,b,c | 0.31±0.05 a | 0.40±0.01 a,b | 1.75±0.03 a,b,c | 2.01±0.05 a,b,c | 0.24±0.01 a | 0.22±0.03 a | 2.16±0.05 a,b,c | 18.92±0.12 a,b,c | 2.22±0.06 a,b | 1.27±0.04 a,b,c | 1.24±0.02 a,b,c |

Statistically significant differences (p < 0.05) between the control group (C) and the botanical extracts. (b) Statistically significant differences (p < 0.05) between the formulation (CF) and the botanical extracts. (c) Statistically significant differences (p < 0.05) between commercial biostimulant (CB) and the botanical extracts. Abbreviations: RT, retention time; RI, retention indices; RI_lit, retention indices according to FFNSC (Mondello, 2015); RI_exp, retention indices based on experiments; UAE, ultrasound-assisted extraction; MH, mechanical homogenisation; Hp H, *Hypericum perforatum* L. (St. John's wort, herb); Sg L, *Solidago gigantea* Ait. (giant goldenrod, leaf); To F, To L, *Taraxacum officinale* (L.) Weber ex F.H. Wigg (common dandelion, flower, leaf); Tp F, *Trifolium pratense* L. (red clover, flower); Ur L, *Urtica dioica* L. (nettle, leaf); Vo R, *Valeriana officinalis* L. (valerian, root).

**Table S2 – continuation.** Effect of the foliar application of the botanical extracts on the fatty acids composition (the amount of a single component calculated as a percentage (%) of the whole GC-MS chromatogram area) (N = 3, mean ± SD) of radish roots (after harvest).

| **Group** | **Heptadecanoic acid, methyl ester** | **Heptadecanoic acid, 16-methyl-, methyl ester** | **Octadecanoic acid, methyl ester** | **9*Z*-9-Octadecenoic acid, ethyl ester** | **11-Octadecenoic acid, methyl ester** | **9,12-Hexadecadienoic acid, methyl ester** | **7,10,13-Hexadecatrienoic acid, methyl ester** | **Linolenic acid, methyl ester** | **Eicosanoic acid, methyl ester** | ***cis*-Methyl 11-eicosenoate** | **Docosanoic acid, methyl ester** | **Tetracosanoic acid, methyl ester** |
| --- | --- | --- | --- | --- | --- | --- | --- | --- | --- | --- | --- | --- |
| RT, min | 31.865 | 33.105 | 34.260 | 34.955 | 35.140 | 36.315 | 37.325 | 37.775 | 38.095 | 40.690 | 41.830 | 42.660 |
| RI_exp | 1703 | 1752 | 1800 | 1836 | 1843 | 1907 | 1960 | 1987 | 2000 | 2041 | 2200 | 2401 |
| RI_lit | 1700 | 1753 | 1800 | 1827 | 1836 | 1901 | 1963 | 1992 | 2000 | 2042 | 2200 | 2400 |
| C | 0.90±0.02 b,c | 0.19±0.00 b,c | 7.05±0.04 b,c | 9.04±0.24 b,c | 6.30±0.22 b,c | 8.32±0.04 b,c | 0.43±0.05 b | 21.48±0.63 | 1.29±0.07 b,c | 1.33±0.02 b,c | 0.87±0.03 b,c | 0.84±0.05 b,c |
| CF | 30.36±0.29 a,c | 0.06±0.01 a | 3.25±0.05 a,c | 5.15±0.35 a,c | 5.00±0.21 a | 6.97±0.23 a | 0.28±0.02 a,c | 20.97±0.36 | 0.80±0.03 a | 0.65±0.02 a | 0.29±0.02 a | 0.61±0.01 a |
| CB | 25.92±0.16 a,b | 0.07±0.00 a | 3.89±0.03 a,b | 7.58±0.19 a,b | 5.23±0.13 a | 7.08±0.24 a | 0.40±0.01 b | 20.46±0.39 | 0.90±0.03 a | 0.73±0.02 a | 0.31±0.03 a | 0.68±0.04 a |
| Hp H UAE | 19.99±0.15 a,b,c | 0.09±0.00 a | 3.81±0.18 a,b | 5.61±0.09 a,c | 5.40±0.30 a | 7.21±0.26 a | 0.36±0.01 | 23.86±0.35 a,b,c | 0.99±0.05 a,b | 0.85±0.03 a,b | 0.38±0.06 a | 0.84±0.03 b,c |
| Hp H MH | 25.76±0.16 a,b | 0.16±0.07 b,c | 3.46±0.13 a | 5.85±0.10 a,c | 5.01±0.03 a | 6.96±0.24 a | 0.55±0.03 a,b,c | 21.00±0.51 | 1.11±0.08 a,b,c | 0.70±0.04 a | 0.43±0.08 a | 0.91±0.02 b,c |
| Sg L UAE | 25.20±0.36 a,b | 0.12±0.01 | 3.79±0.07 a,b | 4.47±0.07 a,c | 4.52±0.19 a,c | 5.92±0.06 a,b,c | 0.32±0.04 | 19.66±0.21 | 0.98±0.04 a,b | 0.68±0.09 a | 0.13±0.01 a,c | 0.70±0.07 |
| Sg L MH | 14.88±0.15 a,b,c | 0.06± 0.00 a | 3.68± 0.09 a | 6.24± 0.12 a,b,c | 6.24± 0.05 b,c | 8.86± 0.20 b,c | 0.47± 0.02 b | 24.56±0.90 a,b,c | 0.99± 0.03 a,b | 0.77± 0.03 a | 0.53± 0.07 a,b,c | 0.81± 0.04 b |
| To F UAE | 14.35±0.05 a,b,c | 0.18±0.02 b,c | 4.28±0.12 a,b | 5.52±0.11 a,c | 5.81±0.18 b | 7.83±0.15 b | 0.34±0.02 | 21.82±0.56 | 1.01±0.04 a,b | 0.98±0.03 a,b,c | 0.19±0.01 a | 0.94±0.03 b,c |
| To F MH | 37.31±0.68 a,b,c | 0.08±0.02 a | 3.29±0.19 a,c | 4.25±0.48 a,b,c | 4.37±0.25 a,c | 5.65±0.26 a,b,c | 0.25±0.04 a,c | 17.32±0.26 a,b,c | 0.74±0.00 a | 0.70±0.05 a | 0.14±0.03 a | 0.60±0.04 a |
| To L UAE | 22.72±0.30 a,b,c | 0.14±0.00 b,c | 3.84±0.11 a,b | 5.06±0.06 a,c | 4.97±0.20 a | 6.96±0.24 a | 0.26±0.01 a,c | 20.74±0.46 | 0.93±0.03 a | 0.90±0.03 a,b,c | 0.20±0.01 a | 0.80±0.04 b |
| To L MH | 18.91±0.15 a,b,c | 0.08±0.01 a | 3.35±0.24 a,c | 5.89±0.40 a,c | 5.86±0.18 b | 8.39±0.10 b,c | 0.44±0.02 b | 24.19±0.56 a,b,c | 1.03±0.08 a,b | 0.66±0.04 a | 0.48±0.07 a,b,c | 0.81±0.08 b |
| Tp F UAE | 18.04±0.07 a,b,c | 0.13±0.01 | 3.51±0.09 a | 5.59±0.09 a,c | 5.37±0.23 a | 7.32±0.13 a | 0.32±0.02 | 22.65±0.94 c | 0.86±0.02 a | 0.85±0.02 a,b | 0.19±0.00 a | 0.72±0.02 |
| Tp F MH | 37.84±0.20 a,b,c | 0.07±0.00 a | 3.97±0.03 a,b | 4.86±0.29 a,c | 4.32±0.29 a,c | 5.88±0.32 a,b,c | 0.32±0.02 | 16.19±0.23 a,b,c | 0.76±0.02 a | 0.70±0.00 a | 0.31±0.02 a | 0.61±0.01 a |
| Ur L UAE | 20.34±0.06 a,b,c | 0.06±0.01 a | 3.77±0.23 a | 6.37±0.09 a,b,c | 5.96±0.02 b,c | 7.75±0.32 | 0.37±0.02 | 24.70±0.59 a,b,c | 0.90±0.01 a | 0.88±0.02 a,b,c | 0.19±0.02 a | 0.80±0.02 b |
| Ur L MH | 39.03±0.13 a,b,c | 0.09±0.00 a | 3.59±0.24 a | 4.87±0.01 a,c | 4.09±0.24 a,b,c | 5.10±0.12 a,b,c | 0.26±0.02 a,c | 15.65±0.03 a,b,c | 0.77±0.02 a | 0.79±0.03 a | 0.46±0.06 a | 0.63±0.02 a |
| Vo R UAE | 19.12±0.27 a,b,c | 0.11±0.02 | 3.47±0.06 a | 4.64±0.13 a,c | 5.23±0.10 a | 6.23±0.24 a,c | 0.49±0.06 b | 23.71±0.13 a,b,c | 0.89±0.07 a | 0.64±0.02 a | 0.41±0.04 a | 0.65±0.03 a |
| Vo R MH | 15.22±0.02 a,b,c | 0.12±0.01 | 4.14±0.12 a,b | 6.19±0.32 a,b,c | 6.22±0.02 b,c | 7.98±0.13 b,c | 0.43±0.03 b | 23.48±0.63 a,b,c | 1.37±0.04 b,c | 1.60±0.06 a,b,c | 0.68±0.05 a,b,c | 1.42±0.05 a,b,c |
