# Supplementary figures and images for: Systematic Investigation of the Effects of Seven Plant Extracts on the Physiological Parameters, Yield, and Nutritional Quality of Radish (Raphanus sativus var. sativus)
Source: Front Plant Sci. 2021 Jun 17;12:651152. doi: 10.3389/fpls.2021.651152 (PMC8248541; doi:10.3389/fpls.2021.651152)

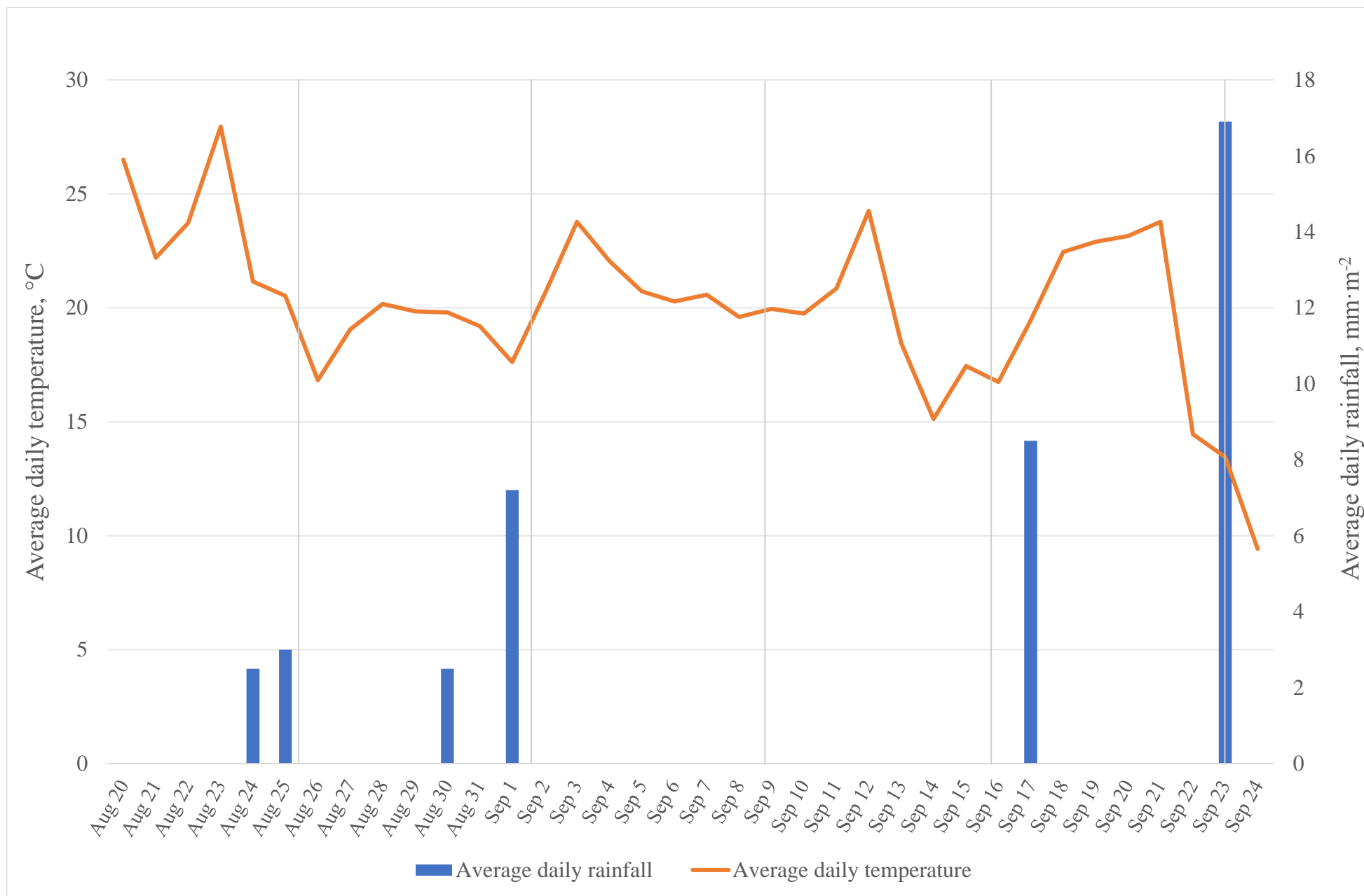

Supplement: Supplementary Figure 1 — The weather conditions during the field experiments. [file Data_Sheet_1.ZIP › Figure S1. The weather conditions during the field experiments..pdf]
